# Childhood sunburn and risk of melanoma and non-melanoma skin cancer: a Mendelian randomization study

**DOI:** 10.1007/s11356-023-30535-3

**Published:** 2023-11-14

**Authors:** Yajia Li, Jianhuang Wu, Ziqin Cao

**Affiliations:** 1 Department of Dermatology, Xiangya Hospital, Central South University, Changsha, Hunan China; 2 National Clinical Research Center for Geriatric Disorders, Xiangya Hospital, Central South University, Changsha, China; 3 Department of Spine Surgery and Orthopaedics, Xiangya Hospital, Central South University, Changsha, China

**Keywords:** Childhood, Sunburn, Skin cancer, Genome-wide association study, Mendelian randomization, Clinical pathology, Genetic background

## Abstract

Previous evidence has suggested that childhood sunburn could be a risk factor for cutaneous malignant melanoma (MM) and non-melanoma skin cancer (NMSC). However, existing observational studies could not reveal the causal associations genetically. This study aimed to investigate whether there was a genetic causal relationship between childhood sunburn and skin cancers. Univariable Mendelian randomization (MR) and Causal Analysis Using Summary Effect analysis was carried out for causal estimates and evaluation for the horizontal pleiotropy. Multivariable MR and the mediation effects analysis were used to test whether the causal associations were mediated by potential confounders. A suggestively significant causal association between childhood sunburn and MM was indicated (OR = 4.74; 95% CI: 1.31–17.19; *p* = 1.79E-02). Genetically predicted childhood sunburn was significantly associated with increased risk of overall melanoma *in situ* (MIS) (OR = 4.02; 95% CI: 2.00–8.08; *p* = 9.40E-05), MIS of face (OR = 18.28; 95% CI: 5.28–63.35; *p* = 4.59E-06), and MIS of trunk (OR = 7.05; 95% CI: 2.06–24.13; *p* = 1.88E-03). Similar trends were found for childhood sunburn and NMSC (OR = 8.16; 95% CI: 6.07–10.99; *p* = 1.53E-20), including both basal cell carcinoma (BCC) (OR = 3.76; 95% CI:2.96–4.77; *p* = 2.19E-08) and squamous cell carcinoma (SCC) (OR = 7.44; 95% CI: 5.09–10.87; *p* = 2.19E-08). After adjustment for hair and skin color, facial ageing, vitamin D levels, body mass index, alcohol consumption, and smoking status, childhood sunburn showed an independent association with MIS, MIS of face, MIS of trunk, as well as NMSC, including both BCC and SCC. Mediation analysis showed no significant mediation effect. This study demonstrated a causal relationship between childhood sunburn and the risk of both MM and NMSC, which suggested that enhanced screening and prevention for childhood sunburn could contribute to the early detection and decreased risk of MM and NMSC.

**Supplementary Information:**

The online version contains supplementary material available at 10.1007/s11356-023-30535-3.

## Introduction

Skin cancers, including cutaneous melanoma and keratinocyte carcinomas, such as basal cell carcinoma (BCC) and squamous cell carcinoma (SCC), are considered as the most common cancer in human, especially common among fair-skinned populations (Sung et al. 2021; Perez et al. 2022). Melanoma is the most invasive cutaneous cancer and the most prone to metastasis, and it accounts for only 2% of skin cancer diagnoses but 80% of related deaths (Geller et al. 2007; Bibbins-Domingo et al. 2016; Wang et al. 2023). The rapid increases in BCC and SCC, which respectively affect over 2.8 million and 1.5 million US citizens, are responsible for a high economic burden on the health care system (Perez et al. 2022). Identification of skin cancer risk factors would facilitate an understanding of the pathogenesis and indicate directions for disease prevention and treatment (Lagacé et al. 2023).

Avoiding exposure to ultraviolet radiation (UV) in daily life is considered effective for reducing the risks of malignant melanoma (MM) and non-melanoma skin cancers (NMSCs) (Berwick et al. 2016; Kricker et al. 2017). Sunburn, resulting from overexposure to UV, is widely accepted as one of the most common potential clinical risk factors for melanoma, BCC, and SCC (Dennis et al. 2008; Khalesi et al. 2013). Sunburn, occurring most often in the early stages of life (mostly before the age of 20), is associated with a higher risk of development of melanoma in a lifetime (Dennis et al. 2008; Green et al. 2011). Several polygenic traits have been correlated with skin cancer risk (Soura and Stratigos 2019; Farré et al. 2023), but to date, any causal role of childhood sunburn remains poorly understood.

Recognition of the causal associations of childhood sunburn with MM and NMSCs may facilitate a deeper understanding of its etiology in cutaneous cancers. However, the limited ability of observational designs to explore the causality was caused by the reverse causality and potential confounders (Arsenault 2022). Mendelian randomization (MR), as an epidemiological investigation tool, could offer a new approach to infer causality based on observational designs (Skrivankova et al. 2021). Genetic variants (usually single nucleotide polymorphisms (SNPs)) independent of confounders or reverse causality, which were strongly associated with exposure were used as instrument variables (IVs) and applied to explore a causal association between exposure and outcome (Yavorska and Burgess 2017). Therefore, the purpose of this study is to determine the genetic relationships between childhood sunburn and skin cancers by conducting an MR analysis.

## Materials and methods

### Data sources and instrumental variables selection

The data deployed in this research were publicly available, summary-level large-scale genome-wide association studies (GWAS) datasets validated by the IEU openGWAS and GWAS catalog databases, thus, negating the need for additional ethical approval.

Exposure to childhood sunburn (including 346,955 participants, released in 2018) was obtained from the UK Biobank (UKB) database (UK-biobank ( n.d.)), as well as the datasets of skin color (456,692 participants), hair color (360,270 participants), facial ageing (423,999 participants), BMI (461,460 participants), and alcohol consumption (462346 participants) while smoking status (249,752 participants, released in 2019) (Liu et al. 2019), and vitamin D levels (496,946 participants, released in 2020) (Revez et al. 2020) were obtained from other two large GWAS studies. According to the UKB database, childhood sunburn is defined as counting data and classified through the questionnaire “Before the age of 15, how many times did you suffer sunburn that was painful for at least 2 days or caused blistering?” Similarly, facial ageing, skin color, and hair color were defined as counting data and collected through questionnaires, while BMI, alcohol consumption smoking status, and smoking status were collected as quantitative data. Variants associated with the genetic risk of ICD9/10-coded malignant melanoma (MM) and NMSC (including BCC and SCC) were obtained from the FinnGen database (FINNGEN ( n.d.)). In addition, the FinnGen GWAS dataset on melanoma *in situ* (MIS) and MIS stratified by site, including the face, trunk, lower limb, and upper limb were included. Control GWAS data of cancer-free participants were included for MM, NMSC, MIS, and MIS stratified by site. The information of all the outcomes was presented in the Supplementary Table (1).

Only the populations of Europeans were included to minimize confounding by ancestry. An overview flowchart of the schematic design giving details of GWAS data is shown in Supplementary Figure (1).

Summary statistics of childhood sunburn-related SNPs were designated as alternate IVs (genome-wide significance: *p* < 5 × 10^−8^; clumping algorithm: *r*^2^ = 0.001 and kb = 10000) (Ference et al. 2015). F statistics of ≥10 demonstrated a low risk of weak instrumental bias (Bowden et al. 2016b; Sanderson and Windmeijer 2016).

### Statistical analysis

The following six methods were used in the univariable Mendelian randomization (UVMR) analysis. The inverse-variance weighted (IVW) method, combining Wald estimates of causality for each IV with the assumption of invalid genetic instruments, was the primary method of MR analysis, and other methods were used in a complementary manner due to wider confidence intervals (CIs) (Burgess et al. 2019; Slob and Burgess 2020). MR-Egger regression analysis (Bowden et al. 2015) quantifies pleiotropy across IVs using the slope and intercept of MR-Egger regression and offers an adjusted, robust estimate independent of IV validity. MR pleiotropy residual sum and outlier (MR-PRESSO) (Verbanck et al. 2018) method identifies and adjusts for distorted outliers that contribute to significant pleiotropy and heterogeneity, thereby providing a corrected causal effect estimate. Weighted-median (Bowden et al. 2016a) method yields consistent valid inferences, even with over 50% valid instrumental variables. Bayesian weighted Mendelian randomization (BWMR) (Zhao et al. 2020) obtains reliable causal inferences by correcting for pleiotropy violations and polygenic weak effect uncertainties within a Bayesian weighting framework. MR-Robust Adjusted Profile Score (MRAPS) (Zhao et al. 2018) increases statistical power and offers robust estimates when weak instrumental bias and horizontal pleiotropy are significant.

Multivariable MR (MVMR) analysis (Burgess and Thompson 2015; Rees et al. 2017) was used to supplement UVMR and to jointly detect the causal effects of multiple risk factors. Skin color, hair color, facial ageing, vitamin D levels, (Revez et al. 2020) BMI, alcohol consumption, and smoking status (Liu et al. 2019) were all taken into consideration, and the MVMR was used to evaluate the independent effects of childhood sunburn. A two-step mediation MR analysis was used for exposures and mediators significantly associated with outcome risk in the MVMR, where a mediating effect was found, and the proportion was calculated (Carter et al. 2021).

A *p*-value of statistical significance after Bonferroni correction was 0.0083 (*α* = 0.05/6), and *p*-values between 0.05 and 0.0083 were considered to be suggestive of significance for UVMR results. Predicted genetic associations of childhood sunburn with skin cancer risk are reported per one SD unit increase, and the effect size is presented as odds ratios (OR) with their corresponding 95% confidence intervals (CI). All analyses were performed using TwoSampleMR (version 0.5.6) (Hemani et al. 2018), MR-PRESSO (version 1.0) (Verbanck et al. 2018), and Mendelian randomization (version 0.5.0) (Yavorska and Burgess 2017) packages in R software (version 4.1.2, R Foundation for Statistical Computing, Vienna, Austria).

### Sensitivity analysis

Heterogeneity due to the invalidity of IVs was measured by Cochran’s Q-statistic. A *p*-value of the Q-statistic < 0.05 was considered to indicate significant heterogeneity (Bowden et al. 2018), and then a random-effect IVW model was applied. The MR-Egger and MR-PRESSO methods were deployed to test the violation of the second IV assumption, prompted by directional pleiotropy. To identify unstable SNPs that individually exerted a disproportionately large influence on the results under the Bonferroni corrected threshold, a leave-one-out analysis was conducted. These SNPs would be omitted, and the results would be reassessed accordingly (Burgess and Thompson 2017). Additionally, the MR method of Causal Analysis Using Summary Effect (CAUSE) was applied to verify the stability of the results. Those associations not paralleling CAUSE were likely to have a false-positive association due to incoherent pleiotropy (Morrison et al. 2020).

## Results

Data regarding SNPs relating to childhood sunburn occasions exposure are given in Supplemental Table (2-9). With all the F statistics >10, there indicated no potential weak IVs. Details of sensitivity analysis and outliers are shown in Tables 1 and 2.

**Table 1:** Sensitivity analysis

Exposure	Outcome	nIVs	Heterogeneity test		MR-Egger pleiotropy test		MR-PRESSO global test		MR-PRESSO distorted outlier test		F statistics
			Q (*P*-value)	Adjusted Q (*P*-value)	Intercept (*P*-value)	Adjusted intercept (*P*-value)	RSSobs (*P*-value)	Adjusted RSSobs (*P*-value)	Outlying SNPs	Heterogeneous SNPs	
Childhood sunburn	Malignant melanoma of skin	77	76.7442 (0.4545)	NA	0.0040 (0.8956)	NA	79.2439 (0.4360)	NA	None	None	147.169167
	Non-melanoma skin cancer	63	352.5470 (0.0000)	108.1224 (0.0003)	−0.0169 (0.0156)	−0.0145 (0.0016)	377.9934 (0.0005)	96.7515 (0.0120)	rs111391498, rs117132860, rs12203592, rs1326798, rs139414522, rs17232484, rs6059655, rs9328259	rs11242899, rs117462393, rs1267038, rs12913832, rs1805007, rs1805008, rs9832130	102.2740
	Squamous cell carcinomas of the skin	62	171.3001 (0.0000)	71.8153 (0.1620)	−0.0133 (0.1945)	−0.0069 (0.4327)	182.1107 (0.0005)	74.0937 (0.1815)	rs117462393, rs1437635, rs1805007, rs4840542, rs6882046, rs9832130	rs12203592, rs12913832	147.1692
	Basal cell carcinomas of the skin	61	450.4076 (0.0000)	130.1305 (0.0000)	−0.0076 (0.2352)	−0.0012 (0.8129)	475.5638 (0.0005)	116.5299 (0.0003)	rs11070811, rs111391498, rs11648436, rs1326798, rs17232484, rs4335021	rs10896139, rs11242899, rs117462393, rs12203592, rs12350739, rs12913832, rs1805007, rs1805008, rs6059655, rs9832130	140.3404
	Melanoma in situ	77	88.8928 (0.1480)	NA	−0.0113 (0.4952)	NA	91.1111 (0.1410)	NA	None	None	98.46187758
	Melanoma in situ of face	77	85.2945 (0.2181)	NA	−0.0080 (0.7864)	NA	87.9316 (0.2250)	NA	None	None	147.1691665
	Melanoma in situ of trunk	77	72.8929 (0.5797)	NA	0.0083 (0.7741)	NA	74.2666 (0.6270)	NA	None	None	147.1691665
	Melanoma in situ of lower limb	77	94.4363 (0.0746)	NA	−0.0076 (0.8298)	NA	87.6576 (0.2355)	NA	None	None	207.9723169
	Melanoma in situ of upper limb	77	94.4363 (0.0746)	NA	−0.0076 (0.8298)	NA	97.0187 (0.0730)	NA	None	None	207.9723169

**Table 2:** Sensitivity analysis (controls excluding all cancers)

Exposure	Outcome	nIVs	Heterogeneity test		MR-Egger pleiotropy test		MR-PRESSO global test		MR-PRESSO distorted outlier test		F statistics
			Q (*P*-value)	Adjusted Q (*P*-value)	Intercept (*P*-value)	Adjusted intercept (*P*-value)	RSSobs (*P*-value)	Adjusted RSSobs (*P*-value)	Outlying, SNPs	Heterogeneous SNPs	
Childhood sunburn	Malignant melanoma of skin (controls excluding all cancers)	77	77.2718 (0.4378)	NA	0.0027 (0.9294)	NA	79.8204 (0.4190)	NA	None	None	117.0993
	Non-melanoma skin cancer (controls excluding all cancers)	63	335.7629 (0.0000)	99.9346 (0.0016)	−0.0158 (0.0235)	−0.0123 (0.0127)	358.7217 (0.0005)	104.1980 (0.0030)	rs111391498, rs11242899, rs117132860, rs12203592, rs1267038, rs1326798, rs139414522, rs6059655	rs117462393, rs12913832, rs1805007, rs1805008, rs9832130	59.0080
	Melanoma in situ (controls excluding all cancers)	77	93.3131 (0.0864)	NA	−0.0123 (0.4689)	NA	95.7565 (0.1030)	NA	None	None	121.7496
	Melanoma in situ of face (controls excluding all cancers)	77	84.6292 (0.2331)	NA	−0.0095 (0.7467)	NA	87.1957 (0.2520)	NA	None	None	121.6272
	Melanoma in situ of trunk (controls excluding all cancers)	77	75.3681 (0.4989)	NA	0.0069 (0.8142)	NA	76.8453 (0.5160)	NA	None	None	121.6198
	Melanoma in situ of lower limb (controls excluding all cancers)	77	85.6601 (0.2101)	NA	0.0225 (0.4829)	NA	87.2606 (0.2355)	NA	None	None	167.3362
	Melanoma in situ of upper limb (controls excluding all cancers)	77	95.3887 (0.0657)	NA	−0.0090 (0.8012)	NA	98.1262 (0.0620)	NA	None	None	167.3362

### Results of univariable Mendelian randomization

A causal relationship showing suggestive of significance with no pleiotropy or heterogeneity was shown between childhood sunburn and MM (IVW-OR = 4.74; 95 % CI: 1.31–17.19; *p* = 1.79E-02) and MM with all other cancers excluded (IVW-OR = 5.38; 95% CI: 1.47–19.74; *p* = 1.12E-02). A significant association was also found between genetically determined childhood sunburn and MIS (IVW-OR = 4.02; 95% CI: 2.00–8.08; *p* = 9.40E-05), MIS with all other cancers excluded (IVW-OR = 4.64; 95% CI: 2.26–9.52; *p* = 2.87E-05), MIS of face (IVW-OR = 18.28; 95% CI: 5.28–63.35; *p* = 4.59E-06), MIS of face with all other cancers excluded (IVW-OR = 21.51; 95% CI: 6.19–74.77; *p* = 1.39E-06), MIS of trunk (IVW-OR = 7.05; 95% CI: 2.06–24.13; *p* = 1.88E-03), and MIS of trunk with all other cancers excluded (IVW-OR = 8.15; 95% CI: 2.35–28.29; *p* = 9.46E-04). No genetic association was found between childhood sunburn and MIS of the upper or lower limbs. NMSCs with significant pleiotropy (*p* = 1.61E-03) and heterogeneity (*p* = 2.61E-04) showed a significant causal relationship with childhood sunburn (MR-Egger OR = 8.16; 95% CI: 6.07–10.99; *p* = 1.53E-20), and similar trends were found for NMSC with all other cancers excluded (*p*_for heterogeneity_ = 1.62E-03; *p*_for pleiotropy_ = 1.27E-02; MR-Egger OR = 5.87; 95% CI:3.86–8.93; *p* = 1.42E-11). A higher probability of genetically predicted childhood sunburn was associated with a higher risk of SCC (IVW-OR = 7.44; 95% CI: 5.09–10.87; *p* = 3.07E-25) and BCC (*p*_for heterogeneity_ = 4.27E-07; IVW-_random effect_ OR = 3.76; 95% CI: 2.96–4.77; *p* = 2.19E-08).

The UVMR forest plots of the causal estimates of childhood sunburn on skin carcinoma are presented in Figs. 1 and 2. Overall, the consistency of effect sizes across different methods indicates that confidence may be put in the results of each method. The corresponding scatter plots for the UVMR analysis are shown in Supplementary Figure (2-17). The leave-one-out stability tests (Supplementary Figures 18-33) demonstrate no potentially influential SNPs affecting the causal associations.

**Fig. 1:**
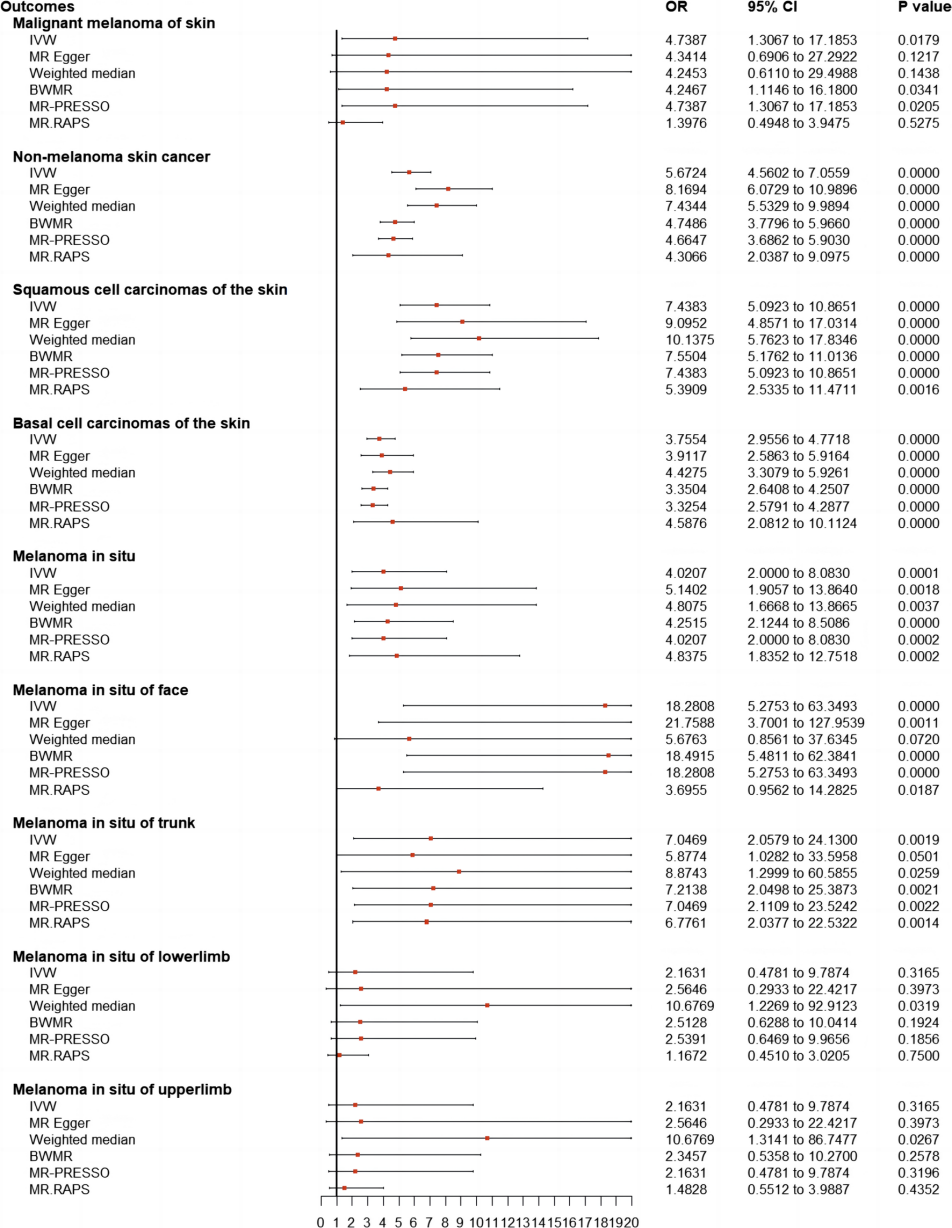
Forest plots to visualize causal effects of childhood sunburn on the skin carcinoma risk. Presented odds ratios (ORs) and confidence intervals (CIs) correspond to the effects of childhood sunburn on malignant melanoma and non-malignant skin cancer. The results of univariable Mendelian randomization (MR) analyses using various analysis methods (IVW, MR-RAPS, MR-Egger, weighed-median estimator, BWMR, MRAPS, MR-PRESSO) are presented for comparison. Total single nucleotide polymorphism (SNP) indicates the number of genetic variants used as instruments for MR analysis. IVW, inverse-variance weighted; BWMR, Bayesian weighted Mendelian randomization; MRAPS, MR-Robust Adjusted Profile Score MR, Mendelian randomization; OR, odds ratios; CI; confidence intervals; SNP, single nucleotide polymorphism

**Fig. 2:**
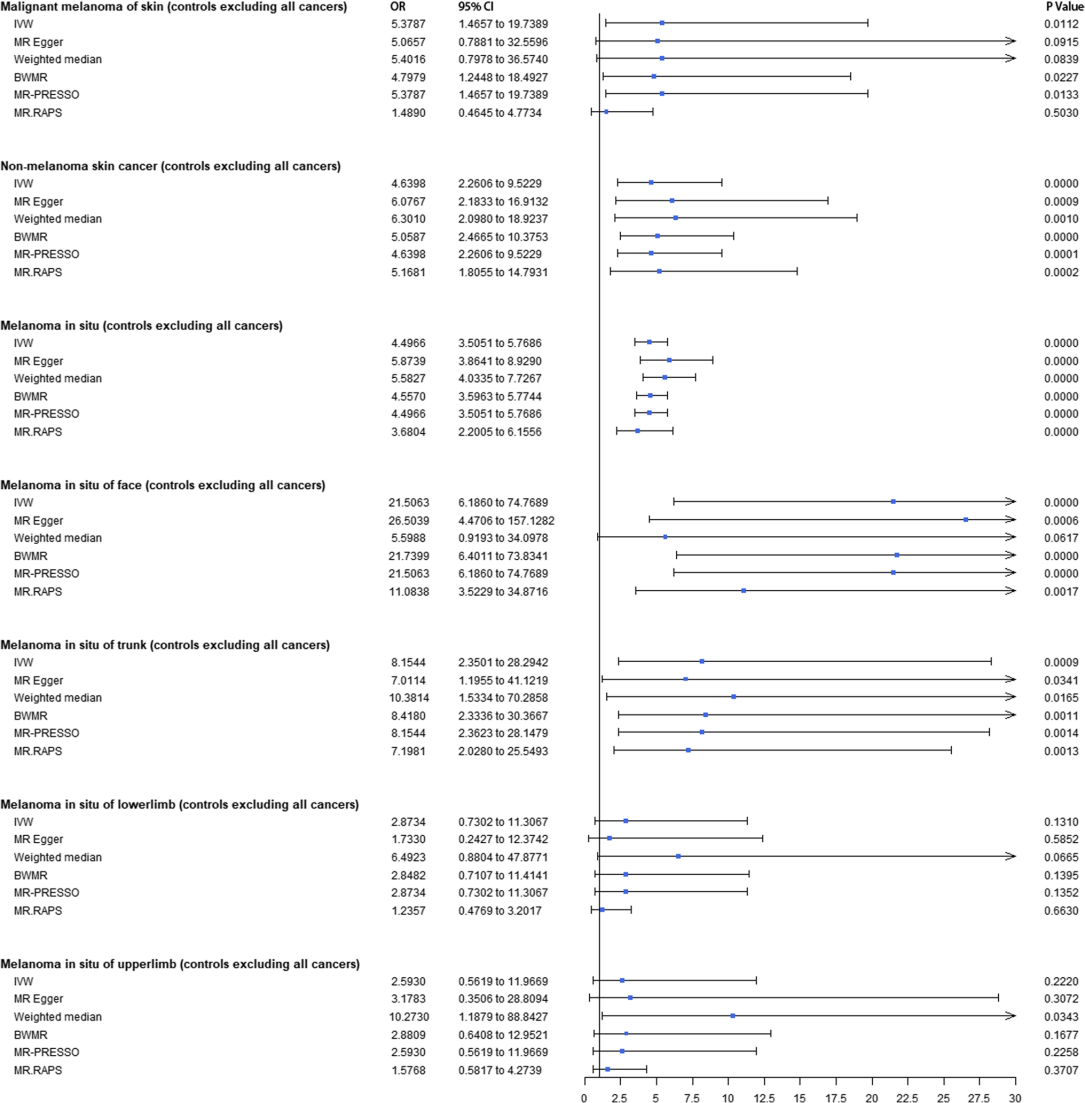
Forest plots to visualize causal effects of childhood sunburn on the skin carcinoma (controls excluding all cancers) risk. Presented odds ratios (ORs) and confidence intervals (CIs) correspond to the effects of childhood sunburn on malignant melanoma and non-malignant skin cancer. The results of univariable Mendelian randomization (MR) analyses using various analysis methods (IVW, MR-RAPS, MR-Egger, weighed-median estimator, BWMR, MRAPS, MR-PRESSO) are presented for comparison. Total single nucleotide polymorphism (SNP) indicates the number of genetic variants used as instruments for MR analysis. IVW, inverse-variance weighted; BWMR, Bayesian weighted Mendelian randomization; MRAPS, MR-Robust Adjusted Profile Score MR, Mendelian randomization; OR, odds ratios; CI; confidence intervals; SNP, single nucleotide polymorphism

CAUSE results identified the associations of childhood sunburn with MM (OR_CAUSE_ = 5.16; 95% CI: 3.71–7.17; *p* = 2.80E-04), MM with all other cancers excluded (OR_CAUSE_ = 5.58; 95% CI: 4.01–7.77; *p* = 3.90E-04), MIS (OR_CAUSE_ = 4.10; 95% CI: 3.46–4.90; *p* = 3.70E-04), MIS with all other cancers excluded (OR_CAUSE_ = 4.85; 95% CI: 4.10–5.81; *p* = 3.50E-04), MIS of face (OR_CAUSE_ =2.75; 95% CI: 2.03-3.71; p =1.10E-02), MIS of face with all other cancers excluded (OR_CAUSE_ =3.06; 95% CI: 2.29–4.10; *p* =5.90E-04), MIS of trunk (OR_CAUSE_ = 7.54; 95% CI: 5.81–9.78; *p* = 1.70E-10), and MIS of trunk with all other cancers excluded (OR_CAUSE_ = 8.85; 95% CI: 6.82–11.59; *p* =4.30E-12). For CAUSE results of NMSCs, similar trends were shown between childhood sunburn and NMSC (OR_CAUSE_ = 1.77; 95% CI: 1.63–1.93; *p* = 1.20E-04), NMSC with all other cancers excluded (OR_CAUSE_ = 1.86; 95% CI: 1.70–2.05; *p* =4.60E-03), as well as BCC (OR_CAUSE_ = 2.72; 95% CI: 2.27–3.29; *p* = 1.30E-02) and SCC (OR_CAUSE_ = 3.46; 95% CI: 2.77–4.35; *p* = 3.70E-02) (Supplementary Figure 34-35).

### Results from multivariable and mediation Mendelian randomization

After adjustment for the skin and hair color, facial ageing, vitamin D levels, BMI, alcohol consumption, and smoking status, a higher probability of genetically predicted childhood sunburn showed an independent association with NMSC (MREgger-OR_MVMR_ = 7.69; 95% CI: 4.64–12.75; *p* = .000), SCC (MREgger-OR_MVMR_ = 13.49; 95% CI: 5.68–32.02; *p* = .000), BCC (MREgger-OR_MVMR_ = 6.55; 95% CI: 4.05–10.62; *p* = .000), NMSC with all other cancers excluded (MREgger-OR_MVMR_ = 6.96; 95% CI: 4.13–11.72; *p* = .000), and MIS with all other cancers excluded (IVW-OR_MVMR_ = 6.43; 95% CI: 1.59 to 26.01; *p* = 9.00E-03).

**Fig. 3:**
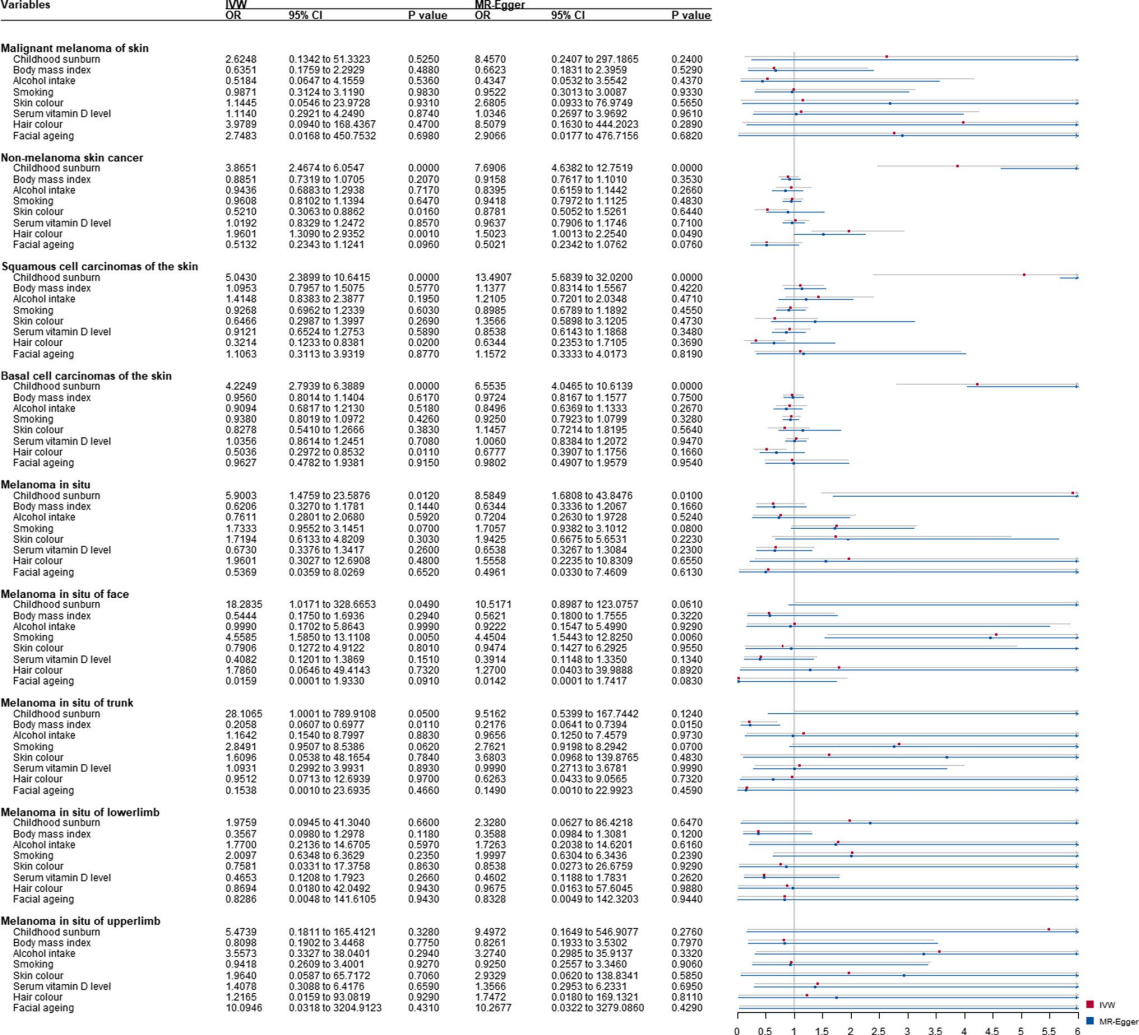
Multivariable Mendelian randomization (MVMR) analysis of childhood sunburn with skin carcinoma risk-adjusted for confounding traits (skin color, hair color, facial ageing, serum vitamin D levels, body mass index, smoking, and alcohol intake). Presented odds ratios (ORs) and confidence intervals (CIs) correspond to the effects of childhood sunburn with skin carcinoma risk. MVMR, multivariable mendelian randomization; OR, odds ratios; CI; confidence intervals

**Fig. 4:**
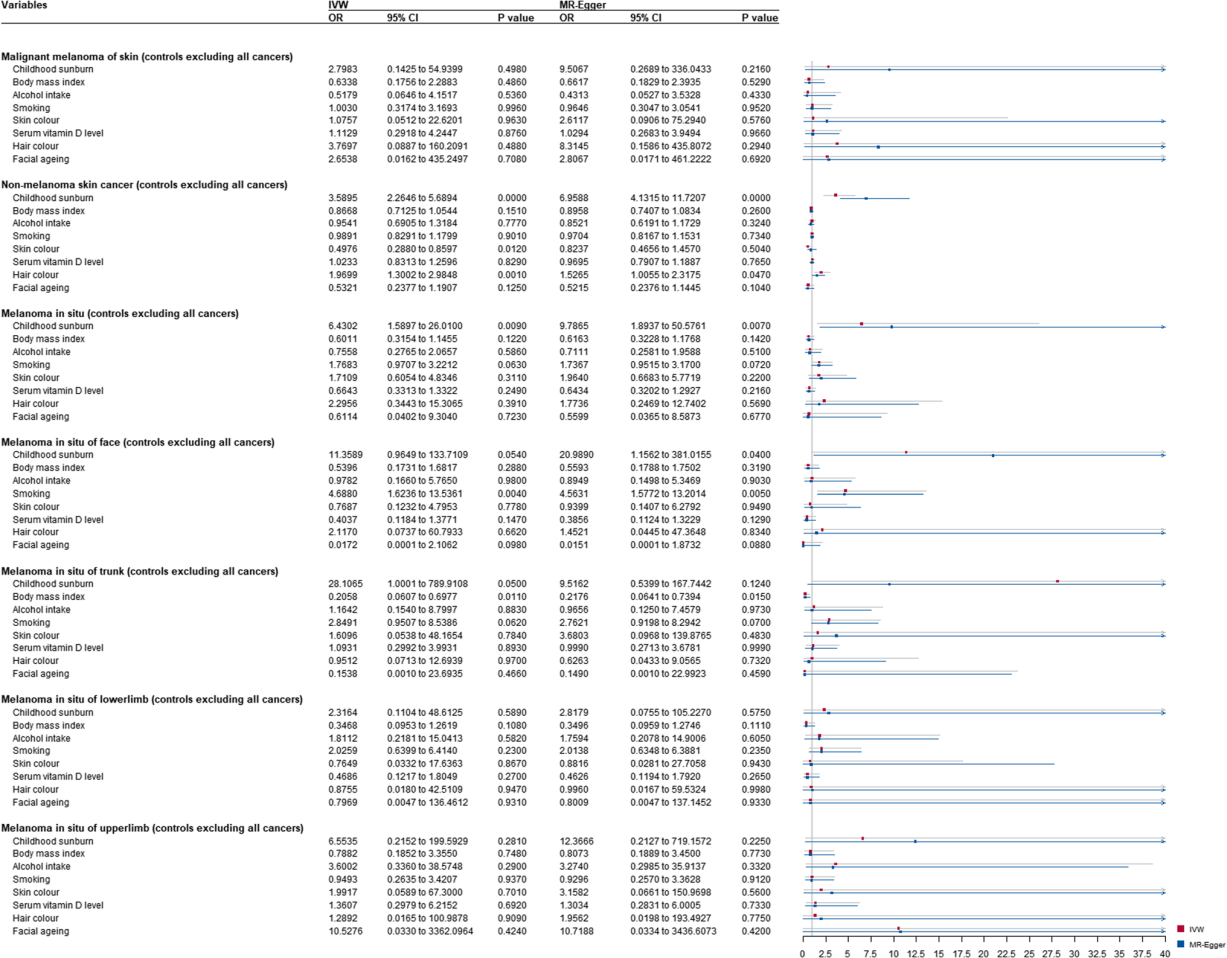
Multivariable mendelian randomization (MVMR) analysis of childhood sunburn with skin carcinoma risk (controls excluding all cancers) adjusted for confounding traits (skin color, hair color, facial ageing, serum vitamin D levels, body mass index, smoking, and alcohol intake). Presented odds ratios (ORs) and confidence intervals (CIs) correspond to the effects of childhood sunburn with skin carcinoma risk (controls excluding all cancers). MVMR, multivariable mendelian randomization; OR, odds ratios; CI; confidence intervals

Significant associations were found in childhood sunburn on MIS (IVW-OR_MVMR_ = 5.90; 95% CI: 1.48 to 23.59; *p* = 1.20E-02), MIS of face (IVW-OR_MVMR_ = 18.28 95% CI: 1.02–328.67; *p* = 4.90E-02), MIS of trunk (IVW-OR_MVMR_ = 28.11; 95% CI: 1.00–789.91; *p* = 5.00E-02), MIS of trunk with all other cancers excluded (IVW-OR_MVMR_ = 28.11; 95% CI: 1.00–789.91; *p* = 5.00E-02) (Figs. 3 and 4). Mediation analysis showed no significant mediation effect (Supplementary Figure 36-37).

## Discussion

A large-scale comprehensive MR analysis was performed to estimate potential causal associations of childhood sunburn with MM and NMSC, and significant causal associations of childhood sunburn with risk of MM, MIS, MIS of face and trunk, as well as NMSC, BCC, and SCC were indicated in univariable analysis. After the adjustment for the skin and hair color, facial ageing, vitamin D levels, BMI, alcohol consumption, and smoking status, there were independent causal associations of childhood sunburn with MIS (overall, trunk, and face), and NMSC (overall, BCC, and SCC).

Some studies investigating childhood sunburns have found increased skin cancer risks with similar effect estimates (range: melanoma, 1.63–3.20; SCC,1.55–2.32) (Kennedy et al. 2003; Gandini et al. 2005; Dennis et al. 2008; de Vries et al. 2012; Savoye et al. 2018). Much heterogeneity has also been observed during previous observational studies, making an investigation of a possible causal association between childhood sunburn and skin cancer necessary (Ghiasvand et al. 2019; Olsen et al. 2020). Sunburn was identified as associated with melanoma risk at all sites with ORs for sunburn in childhood of 1.5 (95% CI: 1.3–1.7) for melanoma of the trunk, 1.5 (95% CI: 1.3–1.7) for the limbs, and 1.4 (95% CI: 1.1–1.7) for the head and neck (Chang et al. 2009). A previous case-control study concluded that sunburn produced a 0.96-fold increase in BCC risk and a 1.02-fold increase in SCC risk (Iannacone et al. 2012). However, Kennedy et al. (2003) found sunburn at ages 0–19 years associated with higher BCC risk but not risk of SCC or melanoma. However, these studies may not have adequately controlled for confounders even when a multivariate regression model was employed. The current findings strengthen the causal genetic association and indicate the possible spectrum of the effects of childhood sunburn, spanning MM, MIS, and NMSCs.

Our results also showed that both skin and non-skin cancers were more commonly seen in severe sunburn patients. Findings in our studies supported that childhood is a susceptible phase for harm from overexposure to the sun and might be a driving factor for MM and NMSC (including BCC and SCC) risk in the present analysis. Besides, the current site-specific analysis of MIS produced an apparent causal association of childhood sunburn with MIS of the face and trunk. The “two pathways to melanoma” hypothesis (Ghiasvand et al. 2019) indicated that more continuous sun exposure might predispose to the development of melanoma on the head and neck, whereas intermittent exposure might predispose to melanoma on the trunk and limbs. Sunburn involves inflammatory reactions, often in response to acute intermittent skin exposure to intense solar radiation (Gandini et al. 2005), which may account for site-specific associations. Further GWAS data relating to specific sites, such as the head and neck, is required to allow a more definite association of childhood sunburn with the risk of site-specific melanoma.

While current evidence supports UV exposure as an important risk factor for cutaneous MM, the exact role of sunburn in the induction of MM has not been fully understood. The development of skin cancer was considered to be directly related to overexposure to UV radiation (Vienneau et al. 2017). Previous evidence showed that epidermal cells might activate phosphoinositide-3-kinase/protein kinase B (PI3K/Akt) and mitogen-activated protein kinase (MAPK) signaling pathways with stimulation of UV, which phosphorylates downstream substrated and could ultimately cause skin carcinoma (Chaiprasongsuk and Panich 2022). The possible etiologic connection of melanoma to solar UV exposure has been contested. It was widely accepted that the mechanisms underlying UV-mediated skin cancer are thought to be most likely related to DNA damage to cutaneous cells. Other biological effects of UV irradiation may contribute to the development of skin cancer through effects on such defenses as pigmentation and the immune response (Wei et al. 2003; Hodis et al. 2012; de Semir et al. 2021). Ultraviolet-radiation-induced inflammation was also identified to promote angiotropism and metastasis in melanoma (Bald et al. 2014). Furthermore, by establishing animal models that mimicked mild sunburn in humans, Viros et al. (2014) found that mutant Trp53, an accepted UVR target in human non-melanoma skin cancer, could accelerate BRAF (V600E)-driven melanomagenesis and that TP53 mutations are linked to evidence of UVR-induced DNA damage in human melanoma, which provided a possible molecular insight into how UVR accelerates melanomagenesis. CD1d, a major histocompatibility complex class 1-like molecule that regulates the function and development of natural killer T (NKT) cells, in promoting UVB-induced cutaneous tissue injury and inflammation was also identified, which suggested that sunburn and NMSC etiologies are immunologically linked (Ryser et al. 2014).

The genetic association of childhood sunburns with skin cancers was identified, and the findings provide supporting evidence that avoiding sunburns, in particular in childhood, is crucial for MM and NMSC prevention. Moreover, taking the potential confounding factors (such as the skin and hair color, facial ageing, vitamin D levels, BMI, alcohol consumption, and smoking status) into consideration, the genetic predisposition to childhood sunburn was still an independent risk for MIS (overall, trunk, and face) and NMSC (overall, BCC, and SCC). These findings appear to have important preventive implications. In light of current results, childhood was found susceptible phase with regard to sunburns and subsequent risk of these skin cancers, supporting evidence that avoiding sunburns throughout childhood, is crucial. Since it seems reasonable that the skin of the unprotected child is more susceptible to UV radiation than the skin of an adult, more attention should be paid to some sun exposure events, such as sunbathing vacations, as well as starting sun protection early in life. Childhood sunburn history could also be taken into consideration for clinical MM and NMSC detection and diagnosis. There were several notable strengths in this study. Firstly, to the best of our knowledge, the current is the first evaluation of causal relationships between childhood sunburn and skin cancer. Secondly, the comprehensive MR approach was less likely to be affected by the potential confounders and reverse causality compared to traditional observational designs, and the findings were confirmed in various sensitivity analyses. Thirdly, the causal relationships between childhood sunburn and specific body sites of MIS were investigated in this analysis, which could contribute to identifying the potential site sensitivity more precisely for MIS prevention guidance. However, we acknowledge some limitations. The only MIS diagnoses divided by body site that are currently available are those from the FinnGen database. Differences between site-specific skin cancers are an essential component of analyses, such as the present one, since distinct mechanisms may be operating at different sites. Besides, further GWAS data relating to specific sites, such as the eyes and ears, is required to allow a more definite association of childhood sunburn with the risk of site-specific MM. Secondly, possible effects of other unmeasured confounders cannot be completely ruled out. For example, since some of the exposure and covariates used in this study were collected through questionnaires, measurement bias may affect the results to some extent. Due to the lack of raw data, we were unable to adjust for possible measurement bias. We will further probe the influence of measure bias on our findings using eligible individual-level data in the future if it is available. Thirdly, GWAS data was limited to patients of European descent, making extrapolation to other populations difficult.

## Conclusion

In conclusion, this MR analysis demonstrated a causal relationship between childhood sunburn and the risk of both MM and NMSC, including BCC and SCC. A genetically predicted higher susceptibility to childhood sunburn contributed to MIS risk, especially that of the face and trunk. The current findings emphasize the importance of avoiding childhood sunburn and ensuring sun protection early in life.

### Supplementary information

ESM 1 (PDF 5970 kb)

ESM 2 (DOCX 217 kb)

## Data Availability

All analyses were conducted using publicly available data. The data that support this study are openly available in UK Biobank at https://www.ukbiobank.ac.uk/, and FinnGen, at https://www.finngen.fi/en. Code Availability: The analysis code in R is available on request.

## Abbreviations

*BCC*: basal cell carcinoma
*BMI*: body mass index
*BWMR*: Bayesian weighted Mendelian randomization
*CAUSE*: Causal Analysis Using Summary Effect
*CIs*: confidence intervals
*GWAS*: Genome-wide association study
*IVW*: inverse-variance weighted
*MAPK*: mitogen-activated protein kinase
*MIS*: melanoma in situ
*MM*: malignant melanoma
*MR*: Mendelian randomization
*MRAPS*: MR-robust adjusted profile score
*MR-PRESSO*: MR pleiotropy residual sum and outlier
*MVMR*: multivariable MR
*NMSC*: non-melanoma skin cancer
*NKT*: natural killer T
*ORs*: odds ratios
*PI3K/Akt*: phosphoinositide-3-kinase/protein kinase B
*SCC*: squamous cell carcinoma
*SNPs*: single nucleotide polymorphisms
*UV*: ultraviolet radiation

## References

[CR1] Arsenault BJ (2022). From the garden to the clinic: how Mendelian randomization is shaping up atherosclerotic cardiovascular disease prevention strategies. Eur Heart J.

[CR2] Bald T, Quast T, Landsberg J, Rogava M, Glodde N, Lopez-Ramos D (2014). Ultraviolet-radiation-induced inflammation promotes angiotropism and metastasis in melanoma. Nature.

[CR3] Berwick M, Buller DB, Cust A, Gallagher R, Lee TK, Meyskens F (2016). Melanoma epidemiology and prevention. Cancer Treat Res.

[CR4] Bibbins-Domingo K, Grossman DC, Curry SJ, Davidson KW, Ebell M, Epling JW (2016). Screening for skin cancer: US preventive services task force recommendation statement. Jama.

[CR5] Bowden J, Davey Smith G, Burgess S (2015). Mendelian randomization with invalid instruments: effect estimation and bias detection through Egger regression. Int J Epidemiol.

[CR6] Bowden J, Davey Smith G, Haycock PC, Burgess S (2016). Consistent estimation in Mendelian randomization with some invalid instruments using a weighted median estimator. Genet Epidemiol.

[CR7] Bowden J, Del Greco MF, Minelli C, Davey Smith G, Sheehan NA, Thompson JR (2016). Assessing the suitability of summary data for two-sample Mendelian randomization analyses using MR-Egger regression: the role of the I2 statistic. Int J Epidemiol.

[CR8] Bowden J, Hemani G, Davey Smith G (2018). Invited commentary: detecting individual and global horizontal pleiotropy in Mendelian randomization-a job for the humble heterogeneity statistic?. Am J Epidemiol.

[CR9] Burgess S, Davey Smith G, Davies NM, Dudbridge F, Gill D, Glymour MM (2019). Guidelines for performing Mendelian randomization investigations. Wellcome Open Res.

[CR10] Burgess S, Thompson SG (2015). Multivariable Mendelian randomization: the use of pleiotropic genetic variants to estimate causal effects. Am J Epidemiol.

[CR11] Burgess S, Thompson SG (2017). Interpreting findings from Mendelian randomization using the MR-Egger method. Eur J Epidemiol.

[CR12] Carter AR, Sanderson E, Hammerton G, Richmond RC, Davey Smith G, Heron J (2021). Mendelian randomisation for mediation analysis: current methods and challenges for implementation. Eur J Epidemiol.

[CR13] Chaiprasongsuk A, Panich U (2022). Role of phytochemicals in skin photoprotection via regulation of Nrf2. Front Pharmacol.

[CR14] Chang YM, Barrett JH, Bishop DT, Armstrong BK, Bataille V, Bergman W (2009). Sun exposure and melanoma risk at different latitudes: a pooled analysis of 5700 cases and 7216 controls. Int J Epidemiol.

[CR15] de Semir D, Bezrookove V, Nosrati M, Dar AA, Miller JR, Leong SP (2021). Nuclear receptor coactivator NCOA3 regulates UV radiation-induced DNA damage and melanoma susceptibility. Cancer Res.

[CR16] de Vries E, Trakatelli M, Kalabalikis D, Ferrandiz L, Ruiz-de-Casas A, Moreno-Ramirez D (2012). Known and potential new risk factors for skin cancer in European populations: a multicentre case-control study. Br J Dermatol.

[CR17] Dennis LK, Vanbeek MJ, Beane Freeman LE, Smith BJ, Dawson DV, Coughlin JA (2008). Sunburns and risk of cutaneous melanoma: does age matter? A comprehensive meta-analysis. Ann Epidemiol.

[CR18] Farré X, Blay N, Cortés B, Carreras A, Iraola-Guzmán S, de Cid R (2023) Skin phototype and disease: a comprehensive genetic approach to pigmentary traits pleiotropy using PRS in the GCAT cohort. Genes (Basel) 14(1). 10.3390/genes14010149 10.3390/genes14010149 PMC9859115 36672889

[CR19] Ference BA, Majeed F, Penumetcha R, Flack JM, Brook RD (2015). Effect of naturally random allocation to lower low-density lipoprotein cholesterol on the risk of coronary heart disease mediated by polymorphisms in NPC1L1, HMGCR, or both: a 2 × 2 factorial Mendelian randomization study. J Am Coll Cardiol.

[CR20] FINNGEN (n.d.). Available: https://finngen.gitbook.io/documentation/ [Accessed].

[CR21] Gandini S, Sera F, Cattaruzza MS, Pasquini P, Picconi O, Boyle P (2005). Meta-analysis of risk factors for cutaneous melanoma: II Sun exposure. Eur J Cancer.

[CR22] Geller AC, Swetter SM, Brooks K, Demierre MF, Yaroch AL (2007). Screening, early detection, and trends for melanoma: current status (2000-2006) and future directions. J Am Acad Dermatol.

[CR23] Ghiasvand R, Robsahm TE, Green AC, Rueegg CS, Weiderpass E, Lund E (2019). Association of phenotypic characteristics and UV radiation exposure with risk of melanoma on different body sites. JAMA Dermatol.

[CR24] Green AC, Wallingford SC, McBride P (2011). Childhood exposure to ultraviolet radiation and harmful skin effects: epidemiological evidence. Prog Biophys Mol Biol.

[CR25] Hemani G, Zheng J, Elsworth B, Wade KH, Haberland V, Baird D et al (2018) The MR-base platform supports systematic causal inference across the human phenome. Elife 7. 10.7554/eLife.34408 10.7554/eLife.34408 PMC5976434 29846171

[CR26] Hodis E, Watson IR, Kryukov GV, Arold ST, Imielinski M, Theurillat JP (2012). A landscape of driver mutations in melanoma. Cell.

[CR27] Iannacone MR, Wang W, Stockwell HG, O'Rourke K, Giuliano AR, Sondak VK (2012). Patterns and timing of sunlight exposure and risk of basal cell and squamous cell carcinomas of the skin--a case-control study. BMC Cancer.

[CR28] Kennedy C, Bajdik CD, Willemze R, De Gruijl FR, Bouwes Bavinck JN (2003). The influence of painful sunburns and lifetime sun exposure on the risk of actinic keratoses, seborrheic warts, melanocytic nevi, atypical nevi, and skin cancer. J Invest Dermatol.

[CR29] Khalesi M, Whiteman DC, Tran B, Kimlin MG, Olsen CM, Neale RE (2013). A meta-analysis of pigmentary characteristics, sun sensitivity, freckling and melanocytic nevi and risk of basal cell carcinoma of the skin. Cancer Epidemiol.

[CR30] Kricker A, Weber M, Sitas F, Banks E, Rahman B, Goumas C (2017). Early life UV and risk of basal and squamous cell carcinoma in New South Wales, Australia. Photochem Photobiol.

[CR31] Lagacé F, Noorah BN, Conte S, Mija LA, Chang J, Cattelan L et al (2023) Assessing skin cancer risk factors, sun safety behaviors and melanoma concern in Atlantic Canada: a comprehensive survey study. Cancers (Basel) 15(15). 10.3390/cancers15153753 10.3390/cancers15153753 PMC10417242 37568569

[CR32] Liu M, Jiang Y, Wedow R, Li Y, Brazel DM, Chen F (2019). Association studies of up to 1.2 million individuals yield new insights into the genetic etiology of tobacco and alcohol use. Nat Genet.

[CR33] Morrison J, Knoblauch N, Marcus JH, Stephens M, He X (2020). Mendelian randomization accounting for correlated and uncorrelated pleiotropic effects using genome-wide summary statistics. Nat Genet.

[CR34] Olsen CM, Pandeya N, Law MH, MacGregor S, Iles MM, Thompson BS (2020). Does polygenic risk influence associations between sun exposure and melanoma? A prospective cohort analysis. Br J Dermatol.

[CR35] Perez M, Abisaad JA, Rojas KD, Marchetti MA, Jaimes N (2022). Skin cancer: primary, secondary, and tertiary prevention. Part I. J Am Acad Dermatol.

[CR36] Rees JMB, Wood AM, Burgess S (2017). Extending the MR-Egger method for multivariable Mendelian randomization to correct for both measured and unmeasured pleiotropy. Stat Med.

[CR37] Revez JA, Lin T, Qiao Z, Xue A, Holtz Y, Zhu Z (2020). Genome-wide association study identifies 143 loci associated with 25 hydroxyvitamin D concentration. Nat Commun.

[CR38] Ryser S, Schuppli M, Gauthier B, Hernandez DR, Roye O, Hohl D (2014). UVB-induced skin inflammation and cutaneous tissue injury is dependent on the MHC class I-like protein, CD1d. J Invest Dermatol.

[CR39] Sanderson E, Windmeijer F (2016). A weak instrument [formula: see text]-test in linear IV models with multiple endogenous variables. J Econom.

[CR40] Savoye I, Olsen CM, Whiteman DC, Bijon A, Wald L, Dartois L (2018). Patterns of ultraviolet radiation exposure and skin cancer risk: the E3N-SunExp study. J Epidemiol.

[CR41] Skrivankova VW, Richmond RC, Woolf BAR, Yarmolinsky J, Davies NM, Swanson SA (2021). Strengthening the reporting of observational studies in epidemiology using mendelian randomization: the STROBE-MR statement. Jama.

[CR42] Slob EAW, Burgess S (2020). A comparison of robust Mendelian randomization methods using summary data. Genet Epidemiol.

[CR43] Soura E, Stratigos A (2019). Implementing polygenic risk scores in skin cancer: a step towards personalized risk prediction. Br J Dermatol.

[CR44] Sung H, Ferlay J, Siegel RL, Laversanne M, Soerjomataram I, Jemal A (2021). Global cancer statistics 2020: GLOBOCAN estimates of incidence and mortality worldwide for 36 cancers in 185 countries. CA Cancer J Clin.

[CR45] UK-biobank. (n.d.) Available: https://www.ukbiobank.ac.uk/ [Accessed]

[CR46] Verbanck M, Chen CY, Neale B, Do R (2018). Detection of widespread horizontal pleiotropy in causal relationships inferred from Mendelian randomization between complex traits and diseases. Nat Genet.

[CR47] Vienneau D, de Hoogh K, Hauri D, Vicedo-Cabrera AM, Schindler C, Huss A (2017). Effects of radon and UV exposure on skin cancer mortality in Switzerland. Environ Health Perspect.

[CR48] Viros A, Sanchez-Laorden B, Pedersen M, Furney SJ, Rae J, Hogan K (2014). Ultraviolet radiation accelerates BRAF-driven melanomagenesis by targeting TP53. Nature.

[CR49] Wang X, Ren Z, Xu Y, Gao X, Huang H, Zhu F (2023). KCNQ1OT1 sponges miR-34a to promote malignant progression of malignant melanoma via upregulation of the STAT3/PD-L1 axis. Environ Toxicol.

[CR50] Wei Q, Lee JE, Gershenwald JE, Ross MI, Mansfield PF, Strom SS (2003). Repair of UV light-induced DNA damage and risk of cutaneous malignant melanoma. J Natl Cancer Inst.

[CR51] Yavorska OO, Burgess S (2017). Mendelian randomization: an R package for performing Mendelian randomization analyses using summarized data. Int J Epidemiol.

[CR52] Zhao J, Ming J, Hu X, Chen G, Liu J, Yang C (2020). Bayesian weighted Mendelian randomization for causal inference based on summary statistics. Bioinformatics.

[CR53] Zhao Q, Wang J, Hemani G, Bowden J, Small DS (2018) Statistical inference in two-sample summary-data Mendelian randomization using robust adjusted profile score. Ann Stat 48(3). 10.48550/arXiv.1801.09652

