# Supplementary material for: Childhood sunburn and risk of melanoma and non-melanoma skin cancer: a Mendelian randomization study
Source: Environ Sci Pollut Res Int. 2023 Nov 14;30(58):122011–23. doi: 10.1007/s11356-023-30535-3 (PMC10724097; doi:10.1007/s11356-023-30535-3)
Supplement: Supplementary file 1 — (PDF 5970 kb) [file 11356_2023_30535_MOESM1_ESM.pdf]

Supplementary Figure 1. (A) Diagram for key assumptions of MR analyses. Childhood sunburn SNPs were used as the genetic instruments to investigate the causal effect of childhood sunburn upon melanoma and non-melanoma Skin Cancer. Line with arrows indicate that the genetic instruments (SNPs) are associated with the exposure and could only affect the outcome via the exposure. Dashed lines indicate that the genetic instruments (SNPs) are independent of any confounding variables between the results. (B) Schematic design for the mendelian randomization analysis. MM, malignant melanoma; NMSC, non-melanoma skin cancer; MIS, melanoma *in situ*; BCC, basal cell carcinoma; SCC, squamous cell carcinoma.

Supplementary Figure 2. Scatter plots of the univariable mendelian randomization analysis of childhood sunburn and malignant melanoma of skin. The slope of each line corresponding to the estimated MR effect in different models, including the conventional IVW, MBE, WMM, MR-Egger, MR-RAPS and MR-PRESSO methods.

Supplementary Figure 3. Scatter plots of the univariable mendelian randomization analysis of childhood sunburn and non-melanoma skin cancer. The slope of each line corresponding to the estimated MR effect in different models, including the conventional IVW, MBE, WMM, MR-Egger, MR-RAPS and MR-PRESSO methods.

Supplementary Figure 4. Scatter plots of the univariable mendelian randomization analysis of childhood sunburn and squamous cell carcinomas of the skin. The slope of each line corresponding to the estimated MR effect in different models, including the conventional IVW, MBE, WMM, MR-Egger, MR-RAPS and MR-PRESSO methods.

Supplementary Figure 5. Scatter plots of the univariable mendelian randomization analysis of childhood sunburn and basal cell carcinomas of the skin. The slope of each line corresponding to the estimated MR effect in different models, including the conventional IVW, MBE, WMM, MR-Egger, MR-RAPS and MR-PRESSO methods.

Supplementary Figure 6. Scatter plots of the univariable mendelian randomization analysis of childhood sunburn and melanoma in situ. The slope of each line corresponding to the estimated MR effect in different models, including the conventional IVW, MBE, WMM, MR-Egger, MR-RAPS and MR-PRESSO methods.

Supplementary Figure 7. Scatter plots of the univariable mendelian randomization analysis of childhood sunburn and melanoma in situ of face. The slope of each line corresponding to the estimated

MR effect in different models, including the conventional IVW, MBE, WMM, MR-Egger, MR-RAPS and MR-PRESSO methods.

Supplementary Figure 8. Scatter plots of the univariable mendelian randomization analysis of childhood sunburn and childhood sunburn on melanoma in situ of trunk. The slope of each line corresponding to the estimated MR effect in different models, including the conventional IVW, MBE, WMM, MR-Egger, MR-RAPS and MR-PRESSO methods.

Supplementary Figure 9. Scatter plots of the univariable mendelian randomization analysis of childhood sunburn and childhood sunburn on melanoma in situ of lowerlimb. The slope of each line corresponding to the estimated MR effect in different models, including the conventional IVW, MBE, WMM, MR-Egger, MR-RAPS and MR-PRESSO methods.

Supplementary Figure 10. Scatter plots of the univariable mendelian randomization analysis of childhood sunburn and childhood sunburn on melanoma in situ of upperlimb. The slope of each line corresponding to the estimated MR effect in different models, including the conventional IVW, MBE, WMM, MR-Egger, MR-RAPS and MR-PRESSO methods.

Supplementary Figure 11. Scatter plots of the replicate univariable mendelian randomization analysis of childhood sunburn and malignant melanoma of skin (controls excluding all cancers). The slope of each line corresponding to the estimated MR effect in different models, including the conventional IVW, MBE, WMM, MR-Egger, MR-RAPS and MR-PRESSO methods.

Supplementary Figure 12. Scatter plots of the replicate univariable mendelian randomization analysis of childhood sunburn and non-melanoma skin cancer (controls excluding all cancers). The slope of each line corresponding to the estimated MR effect in different models, including the conventional IVW, MBE, WMM, MR-Egger, MR-RAPS and MR-PRESSO methods.

Supplementary Figure 13. Scatter plots of the replicate univariable mendelian randomization analysis of childhood sunburn and melanoma in situ (controls excluding all cancers). The slope of each line corresponding to the estimated MR effect in different models, including the conventional IVW, MBE, WMM, MR-Egger, MR-RAPS and MR-PRESSO methods.

Supplementary Figure 14. Scatter plots of the replicate univariable mendelian randomization analysis of childhood sunburn and melanoma in situ of face (controls excluding all cancers). The slope of each line corresponding to the estimated MR effect in different models, including the conventional IVW, MBE, WMM, MR-Egger, MR-RAPS and MR-PRESSO methods.

Supplementary Figure 15. Scatter plots of the replicate univariable mendelian randomization analysis of childhood sunburn and melanoma in situ of trunk (controls excluding all cancers). The slope of each line corresponding to the estimated MR effect in different models, including the conventional IVW, MBE, WMM, MR-Egger, MR-RAPS and MR-PRESSO methods.

Supplementary Figure 16. Scatter plots of the replicate univariable mendelian randomization analysis of childhood sunburn and melanoma in situ of lowerlimb (controls excluding all cancers). The slope of each line corresponding to the estimated MR effect in different models, including the conventional IVW, MBE, WMM, MR-Egger, MR-RAPS and MR-PRESSO methods.

Supplementary Figure 17. Scatter plots of the replicate univariable mendelian randomization analysis of childhood sunburn and melanoma in situ of upperlimb (controls excluding all cancers). The slope of each line corresponding to the estimated MR effect in different models, including the conventional IVW, MBE, WMM, MR-Egger, MR-RAPS and MR-PRESSO methods.

Supplementary Figure 18. Leave-one-out stability tests of the univariable mendelian randomization analysis of childhood sunburn and malignant melanoma of skin. Calculate the MR results of the remaining IVs after removing the IVs one by one.

Supplementary Figure 19. Leave-one-out stability tests of the univariable mendelian randomization analysis of childhood sunburn and non-melanoma skin cancer. Calculate the MR results of the remaining IVs after removing the IVs one by one.

Supplementary Figure 20. Leave-one-out stability tests of the univariable mendelian randomization analysis of childhood sunburn and squamous cell carcinomas of the skin. Calculate the MR results of the remaining IVs after removing the IVs one by one.

Supplementary Figure 21. Leave-one-out stability tests of the univariable mendelian randomization analysis of childhood sunburn and basal cell carcinomas of the skin. Calculate the MR results of the remaining IVs after removing the IVs one by one.

Supplementary Figure 22. Leave-one-out stability tests of the univariable mendelian randomization analysis of childhood sunburn and melanoma in situ. Calculate the MR results of the remaining IVs after removing the IVs one by one.

Supplementary Figure 23. Leave-one-out stability tests of the univariable mendelian randomization analysis of childhood sunburn and melanoma in situ of face. Calculate the MR results of the remaining IVs after removing the IVs one by one.

Supplementary Figure 24. Leave-one-out stability tests of the univariable mendelian randomization analysis of childhood sunburn and melanoma in situ of trunk. Calculate the MR results of the remaining IVs after removing the IVs one by one.

Supplementary Figure 25. Leave-one-out stability tests of the univariable mendelian randomization analysis of childhood sunburn and melanoma in situ of lowerlimb. Calculate the MR results of the remaining IVs after removing the IVs one by one.

Supplementary Figure 26. Leave-one-out stability tests of the univariable mendelian randomization analysis of childhood sunburn and melanoma in situ of upperlimb. Calculate the MR results of the remaining IVs after removing the IVs one by one.

Supplementary Figure 27. Leave-one-out stability tests of the replicate univariable mendelian randomization analysis of childhood sunburn and malignant melanoma of skin (controls excluding all cancers). Calculate the MR results of the remaining IVs after removing the IVs one by one.

Supplementary Figure 28. Leave-one-out stability tests of the replicate univariable mendelian randomization analysis of childhood sunburn and non-melanoma skin cancer (controls excluding all cancers). Calculate the MR results of the remaining IVs after removing the IVs one by one.

Supplementary Figure 29. Leave-one-out stability tests of the replicate univariable mendelian randomization analysis of childhood sunburn and melanoma in situ (controls excluding all cancers). Calculate the MR results of the remaining IVs after removing the IVs one by one.

Supplementary Figure 30. Leave-one-out stability tests of the replicate univariable mendelian randomization analysis of childhood sunburn and melanoma in situ of face (controls excluding all cancers). Calculate the MR results of the remaining IVs after removing the IVs one by one.

Supplementary Figure 31. Leave-one-out stability tests of the replicate univariable mendelian randomization analysis of childhood sunburn and melanoma in situ of trunk (controls excluding all cancers). Calculate the MR results of the remaining IVs after removing the IVs one by one.

Supplementary Figure 32. Leave-one-out stability tests of the replicate univariable mendelian randomization analysis of childhood sunburn and melanoma in situ of lowerlimb (controls excluding all cancers). Calculate the MR results of the remaining IVs after removing the IVs one by one.

Supplementary Figure 33. Leave-one-out stability tests of the replicate univariable mendelian randomization analysis of childhood sunburn and melanoma in situ of upperlimb (controls excluding all cancers). Calculate the MR results of the remaining IVs after removing the IVs one by one.

Supplementary Figure 34. The forest plot of the CAUSE method MR analysis. Causal estimates were given as beta and 95% confidence intervals (CIs).

Supplementary Figure 35. The forest plot of the replicate CAUSE method MR analysis (controls excluding all cancers). Causal estimates were given as beta and 95% confidence intervals (CIs).

Supplementary Figure 36. The forest plot of the mediation MR analyses. Causal estimates given as odds ratio (OR) and 95% confidence intervals (CIs) for the effect of childhood sunburn and skin carcinoma risk. Red:total effect; Green: effect of skin colour; Purple: effect of serum vitamin D levels; Blue: effect of body mass index; Orange: effect of alcohol intake; Black: effect of smoking; Brown: effect of hair colour; Grey: effect of facial ageing.

Supplementary Figure 37. The forest plot of the replicate mediation MR analyses (controls excluding all cancers). Causal estimates given as odds ratio (OR) and 95% confidence intervals (CIs) for the effect of childhood sunburn and skin carcinoma risk. Red:total effect; Green: effect of skin colour; Purple: effect of serum vitamin D levels; Blue: effect of body mass index; Orange: effect of alcohol intake; Black: effect of smoking; Brown: effect of hair colour; Grey: effect of facial ageing.

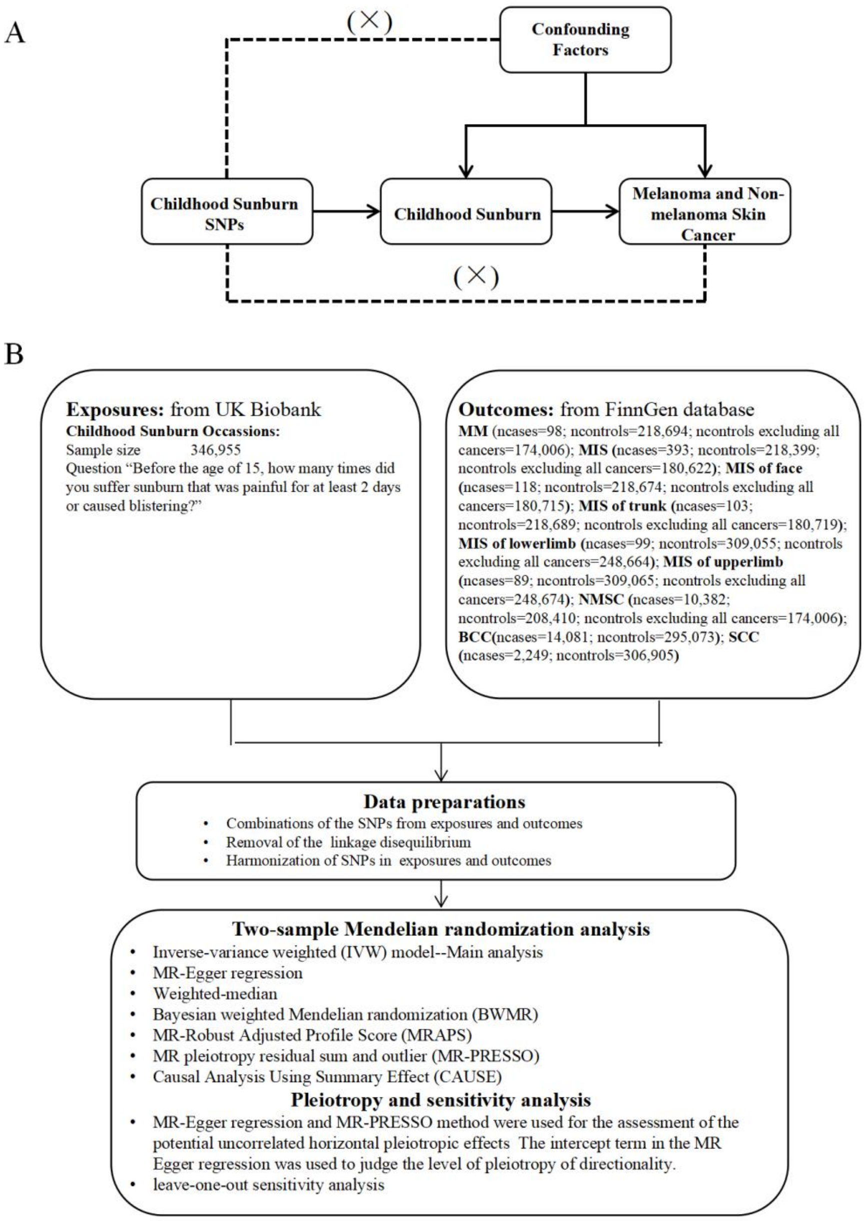

Supplementary Figure 1. (A) Diagram for key assumptions of MR analyses. Childhood sunburn SNPs were used as the genetic instruments to investigate the causal effect of childhood sunburn upon melanoma and non-melanoma Skin Cancer. Line with arrows indicate that the genetic instruments (SNPs) are associated with the exposure and could only affect the outcome via the exposure. Dashed lines indicate that the genetic instruments (SNPs) are independent of any confounding variables between the results. (B) Schematic design for the mendelian randomization analysis. MM, malignant melanoma; NMSC, non-melanoma skin cancer; MIS, melanoma *in situ*; BCC, basal cell carcinoma; SCC, squamous cell carcinoma.

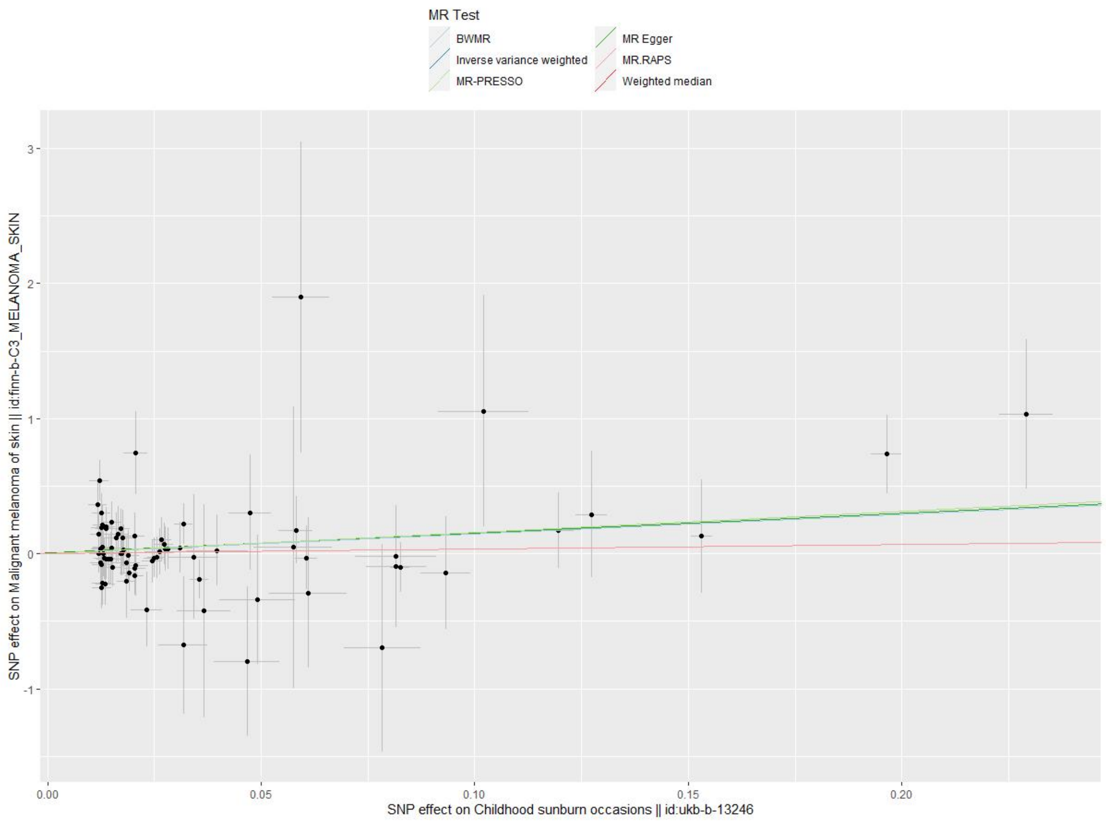

Supplementary Figure 2. Scatter plots of the univariable mendelian randomization analysis of childhood sunburn and malignant melanoma of skin. The slope of each line corresponding to the estimated MR effect in different models, including the conventional IVW, MBE, WMM, MR-Egger, MR-RAPS and MR-PRESSO methods.

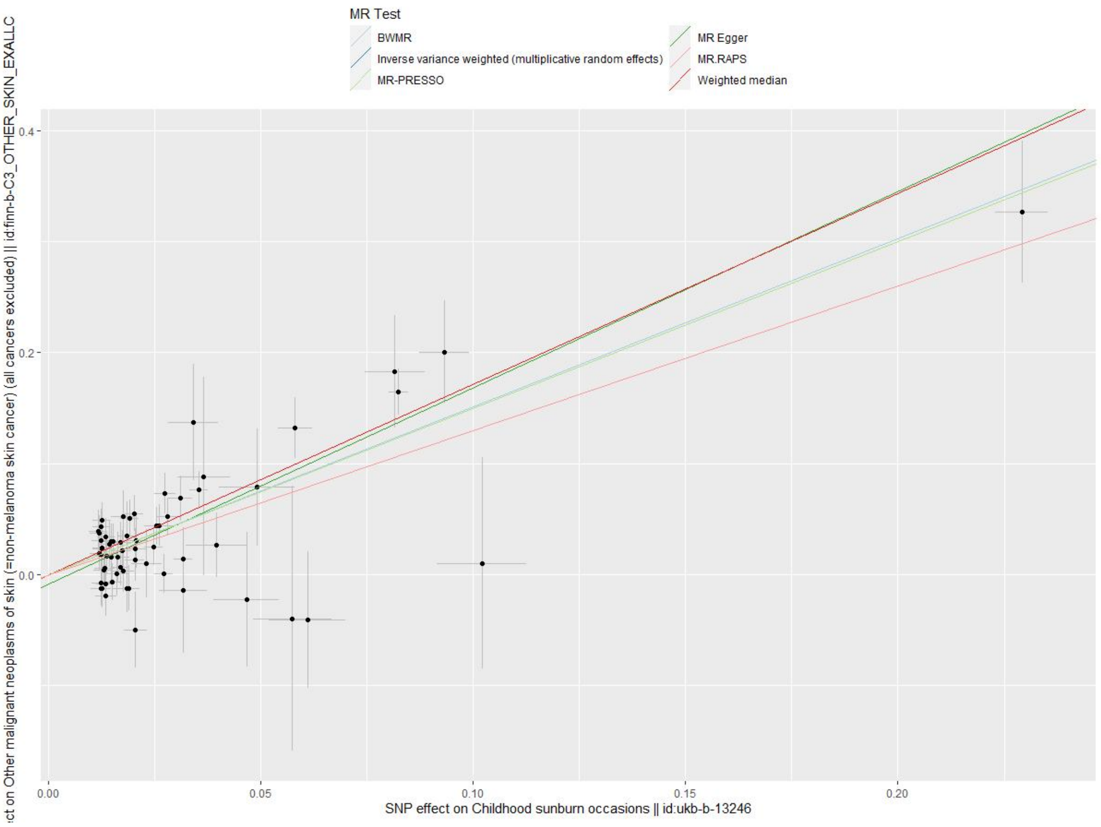

Supplementary Figure 3. Scatter plots of the univariable mendelian randomization analysis of childhood sunburn and non-melanoma skin cancer. The slope of each line corresponding to the estimated MR effect in different models, including the conventional IVW, MBE, WMM, MR-Egger, MR-RAPS and MR-PRESSO methods.

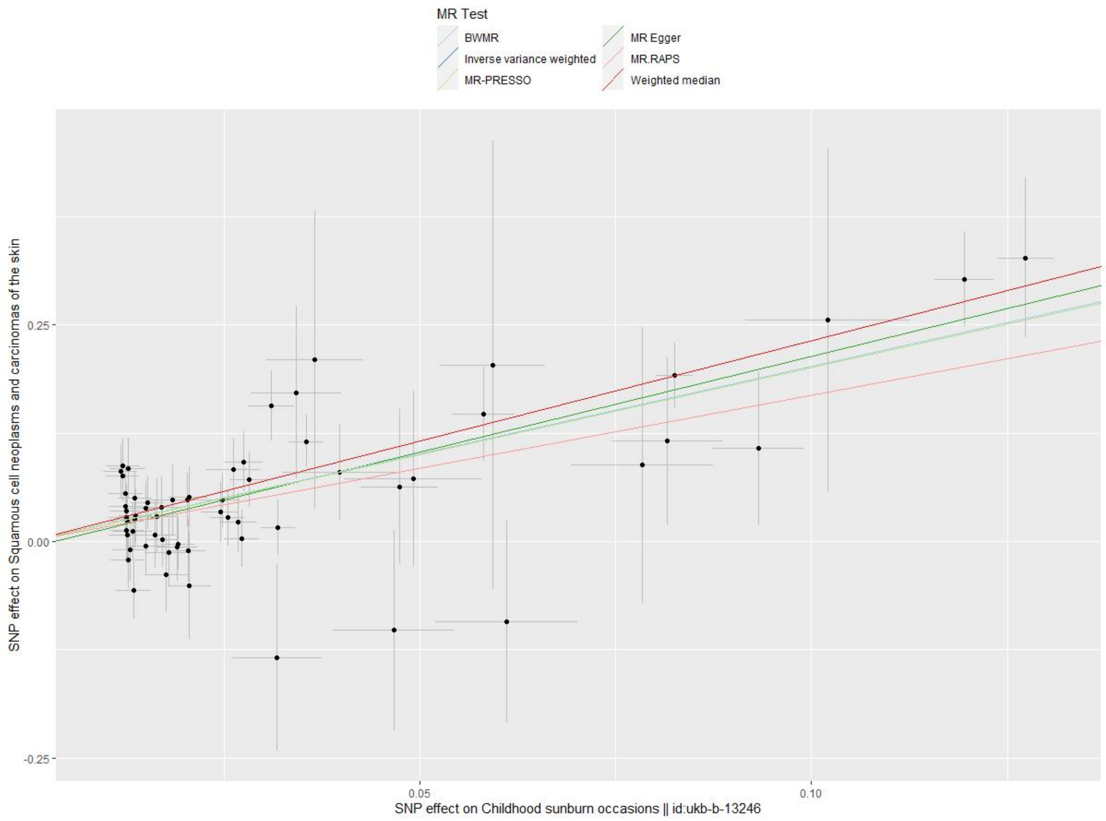

Supplementary Figure 4. Scatter plots of the univariable mendelian randomization analysis of childhood sunburn and squamous cell carcinomas of the skin. The slope of each line corresponding to the estimated MR effect in different models, including the conventional IVW, MBE, WMM, MR-Egger, MR-RAPS and MR-PRESSO methods.

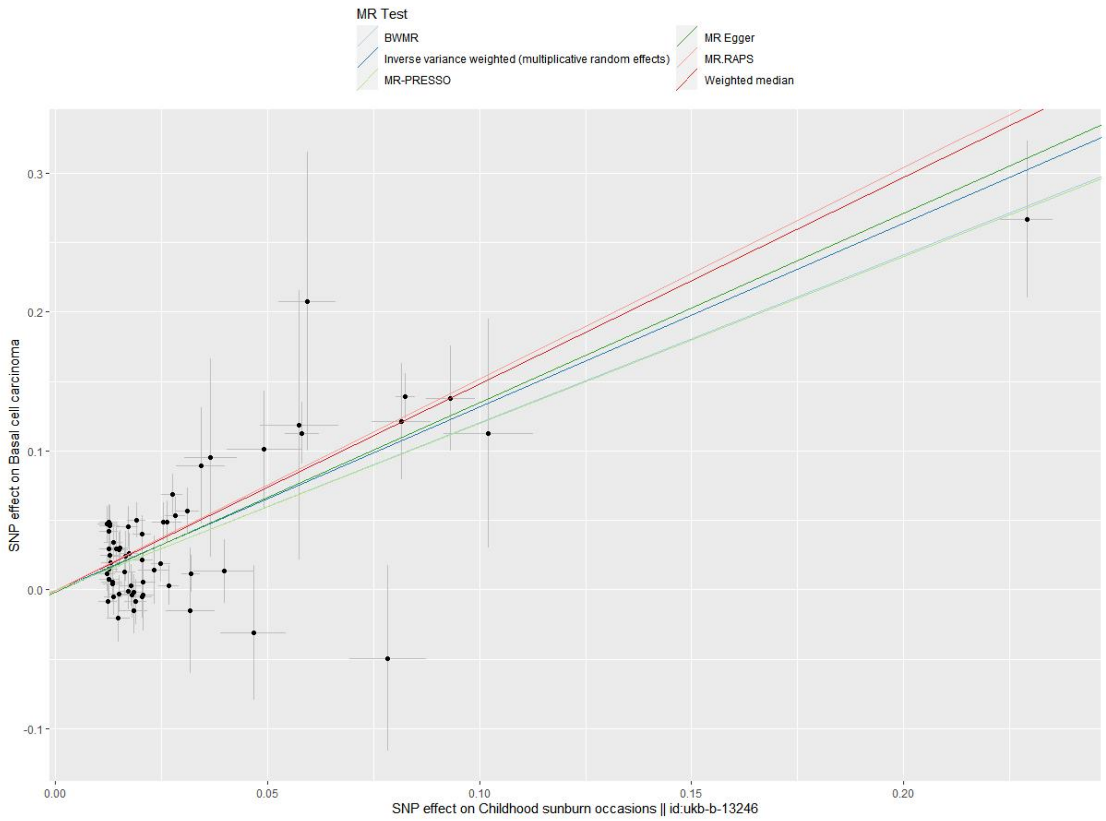

Supplementary Figure 5. Scatter plots of the univariable mendelian randomization analysis of childhood sunburn and basal cell carcinomas of the skin. The slope of each line corresponding to the estimated MR effect in different models, including the conventional IVW, MBE, WMM, MR-Egger, MR-RAPS and MR-PRESSO methods.

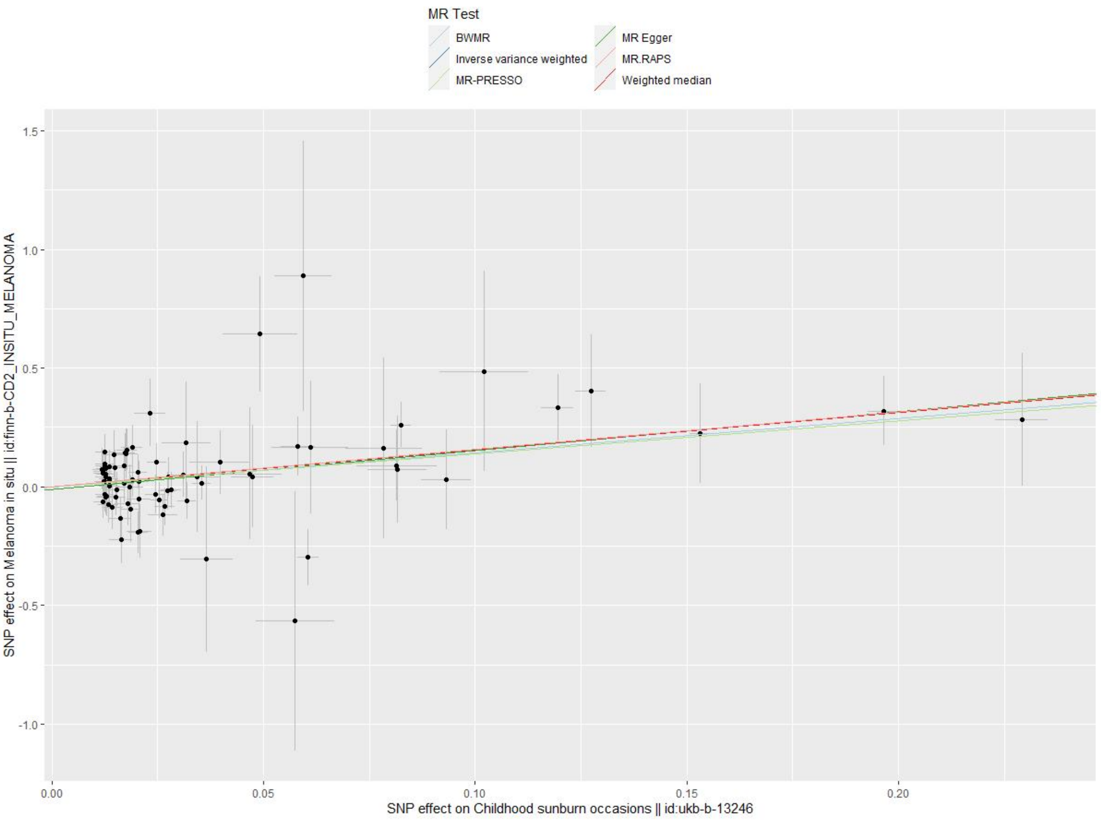

Supplementary Figure 6. Scatter plots of the univariable mendelian randomization analysis of childhood sunburn and melanoma in situ. The slope of each line corresponding to the estimated MR effect in different models, including the conventional IVW, MBE, WMM, MR-Egger, MR-RAPS and MR-PRESSO methods.

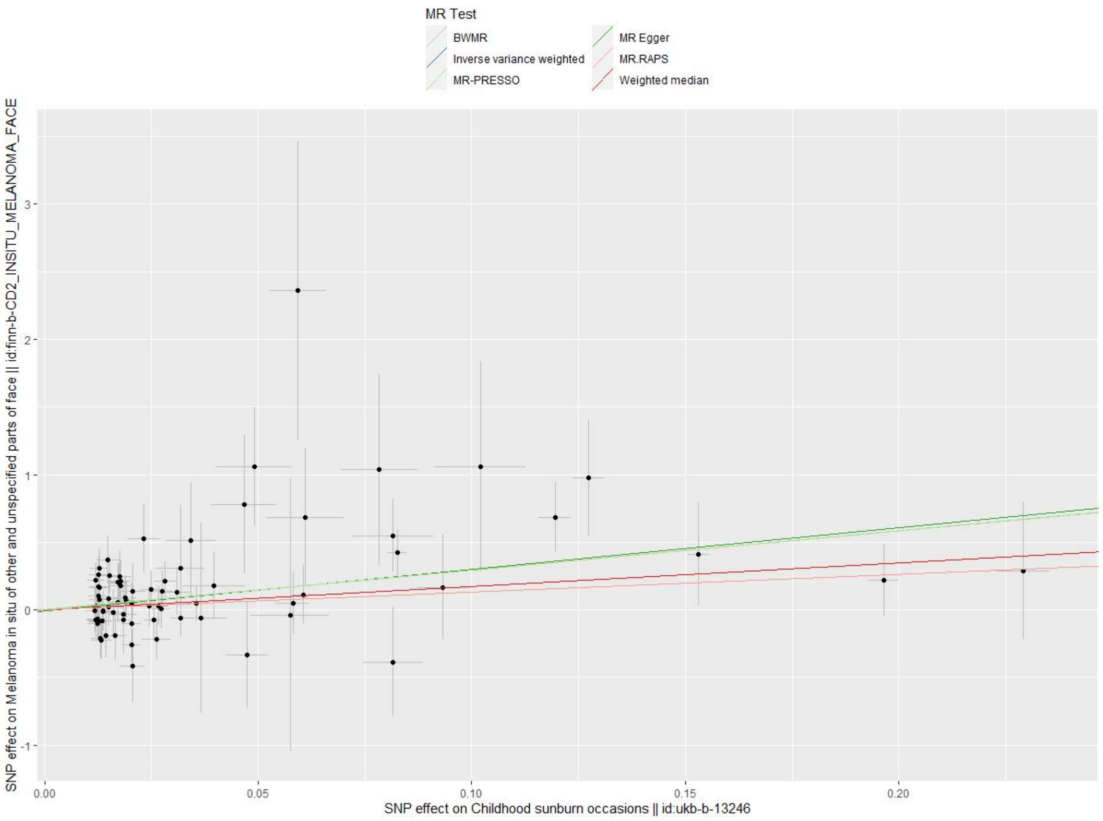

Supplementary Figure 7. Scatter plots of the univariable mendelian randomization analysis of childhood sunburn and melanoma in situ of face. The slope of each line corresponding to the estimated MR effect in different models, including the conventional IVW, MBE, WMM, MR-Egger, MR-RAPS and MR-PRESSO methods.

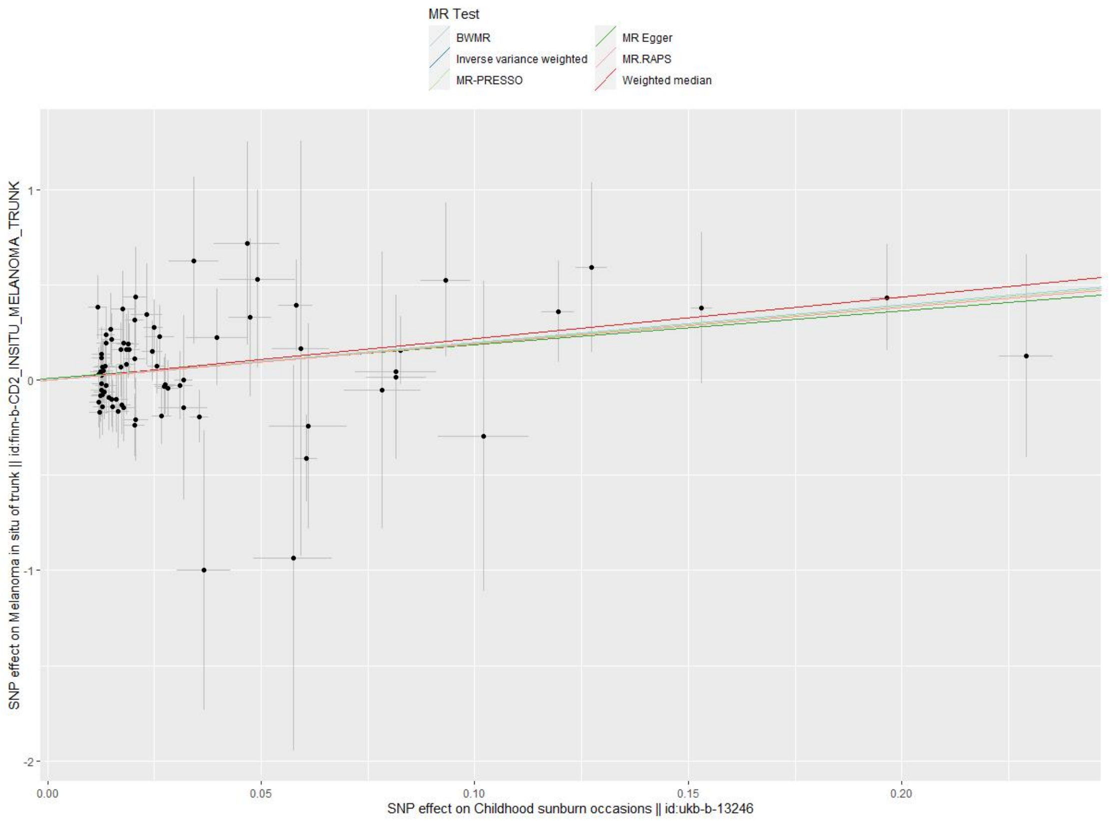

Supplementary Figure 8. Scatter plots of the univariable mendelian randomization analysis of childhood sunburn and childhood sunburn on melanoma in situ of trunk. The slope of each line corresponding to the estimated MR effect in different models, including the conventional IVW, MBE, WMM, MR-Egger, MR-RAPS and MR-PRESSO methods.

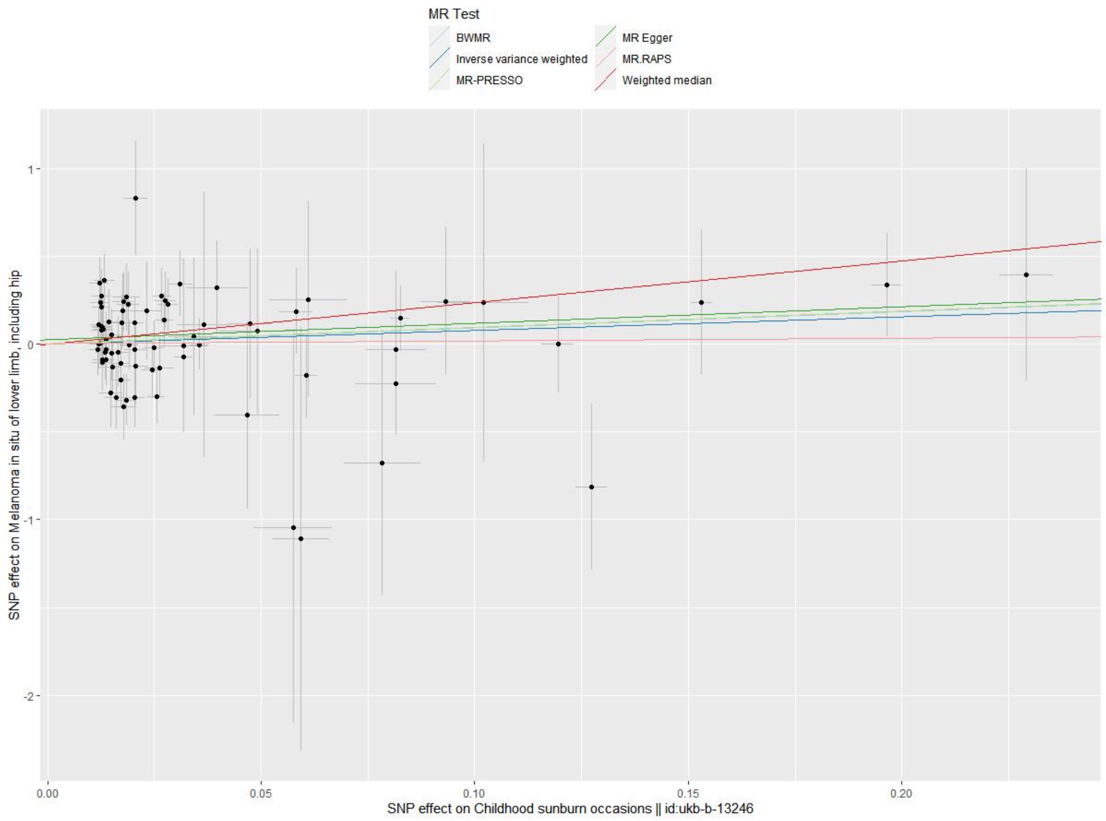

Supplementary Figure 9. Scatter plots of the univariable mendelian randomization analysis of childhood sunburn and childhood sunburn on melanoma in situ of lowerlimb. The slope of each line corresponding to the estimated MR effect in different models, including the conventional IVW, MBE, WMM, MR-Egger, MR-RAPS and MR-PRESSO methods.

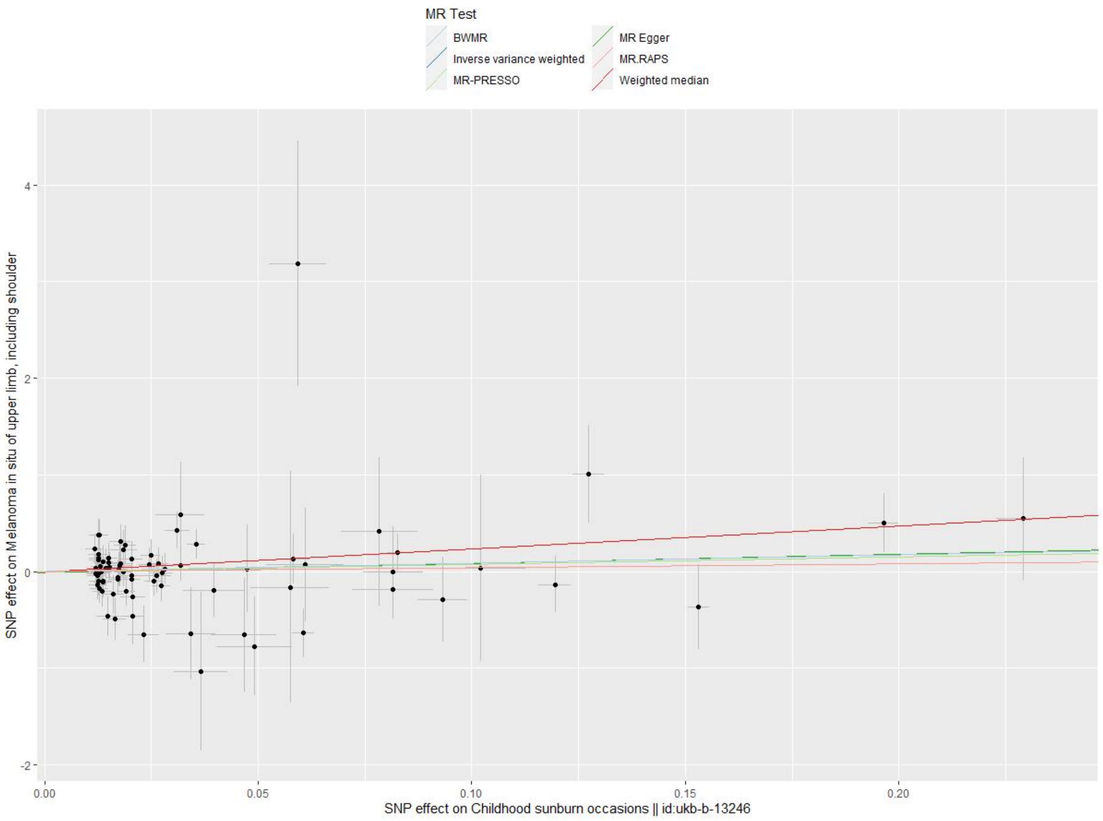

Supplementary Figure 10. Scatter plots of the univariable mendelian randomization analysis of childhood sunburn and childhood sunburn on melanoma in situ of upperlimb. The slope of each line corresponding to the estimated MR effect in different models, including the conventional IVW, MBE, WMM, MR-Egger, MR-RAPS and MR-PRESSO methods.

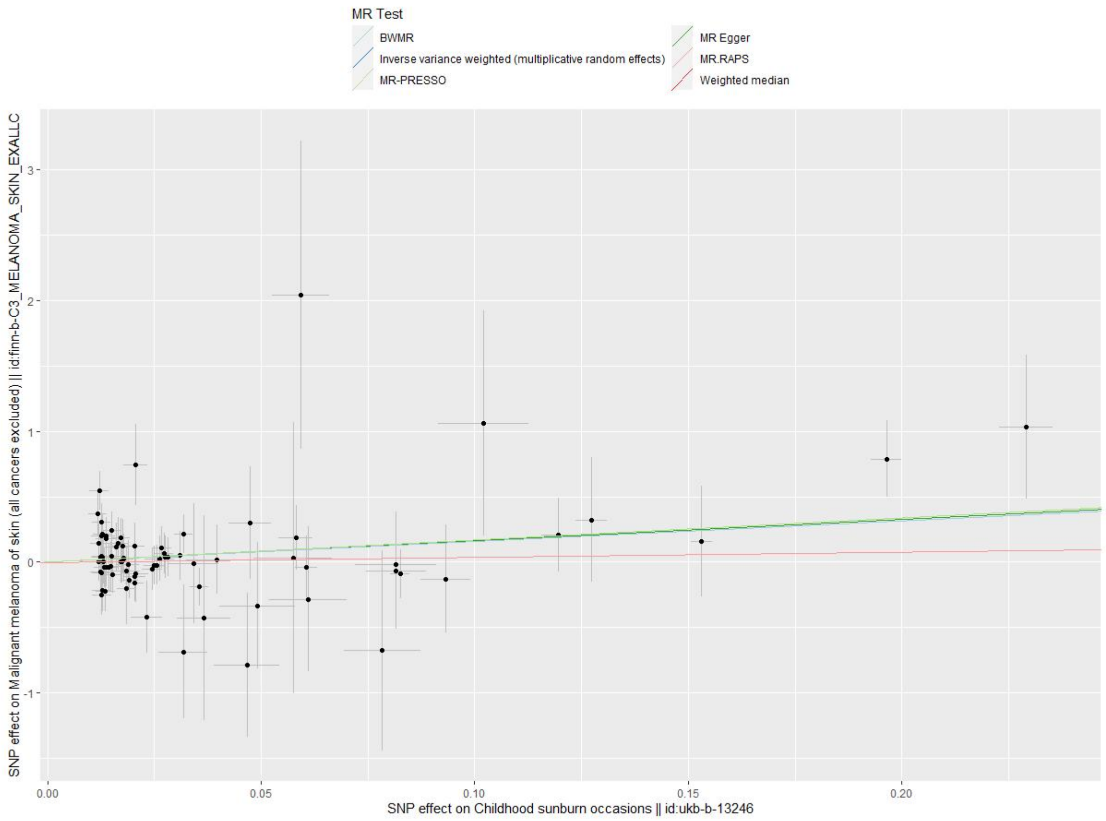

Supplementary Figure 11. Scatter plots of the replicate univariable mendelian randomization analysis of childhood sunburn and malignant melanoma of skin (controls excluding all cancers). The slope of each line corresponding to the estimated MR effect in different models, including the conventional IVW, MBE, WMM, MR-Egger, MR-RAPS and MR-PRESSO methods.

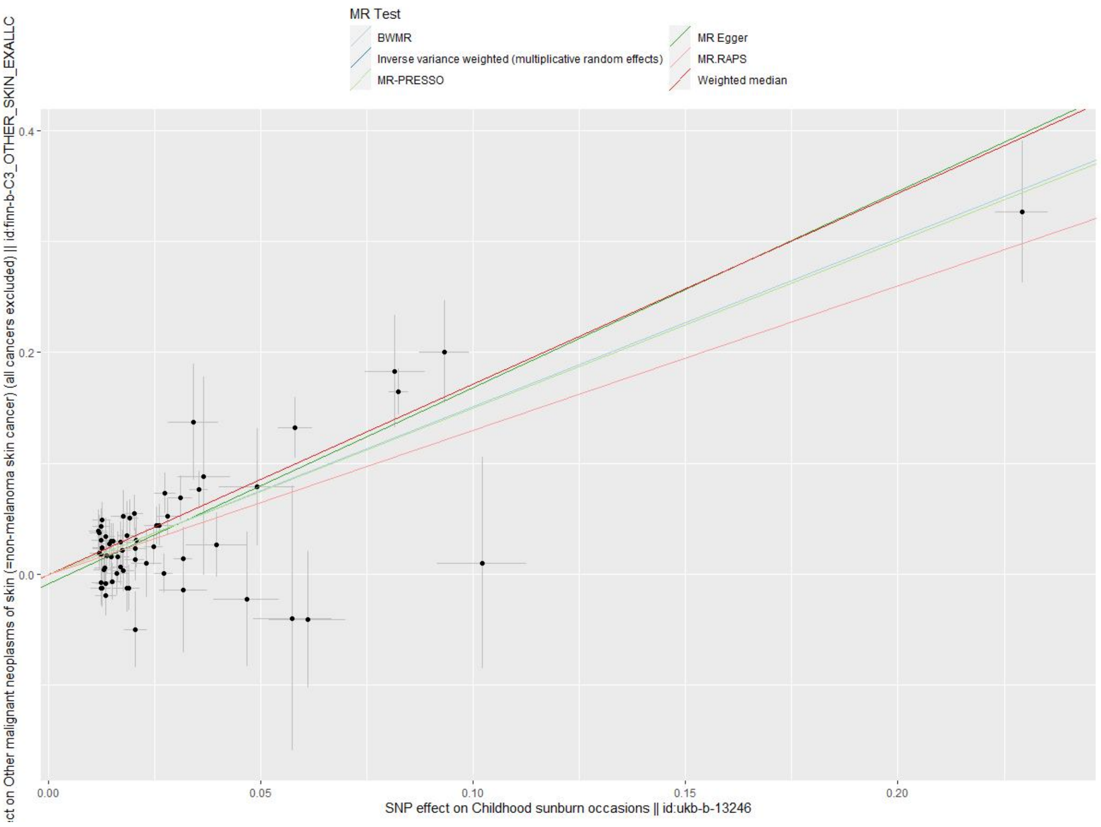

Supplementary Figure 12. Scatter plots of the replicate univariable mendelian randomization analysis of childhood sunburn and non-melanoma skin cancer (controls excluding all cancers). The slope of each line corresponding to the estimated MR effect in different models, including the conventional IVW, MBE, WMM, MR-Egger, MR-RAPS and MR-PRESSO methods.

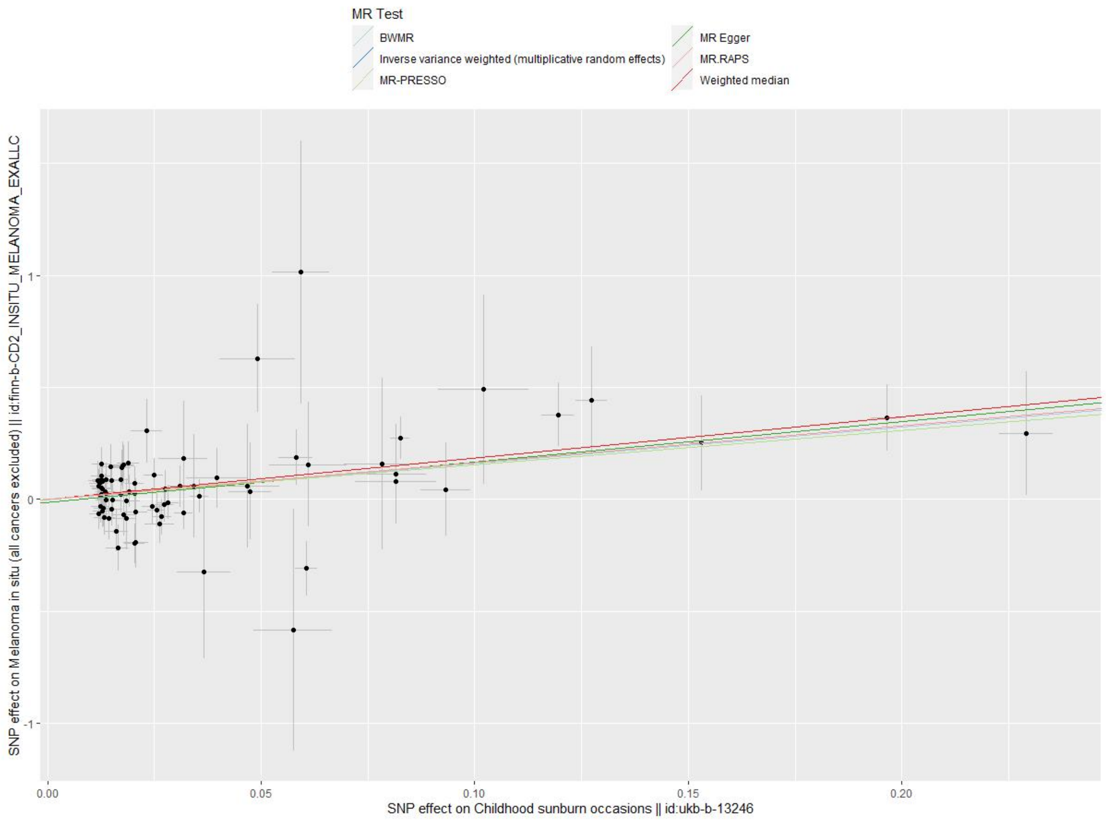

Supplementary Figure 13. Scatter plots of the replicate univariable mendelian randomization analysis of childhood sunburn and melanoma in situ (controls excluding all cancers). The slope of each line corresponding to the estimated MR effect in different models, including the conventional IVW, MBE, WMM, MR-Egger, MR-RAPS and MR-PRESSO methods.

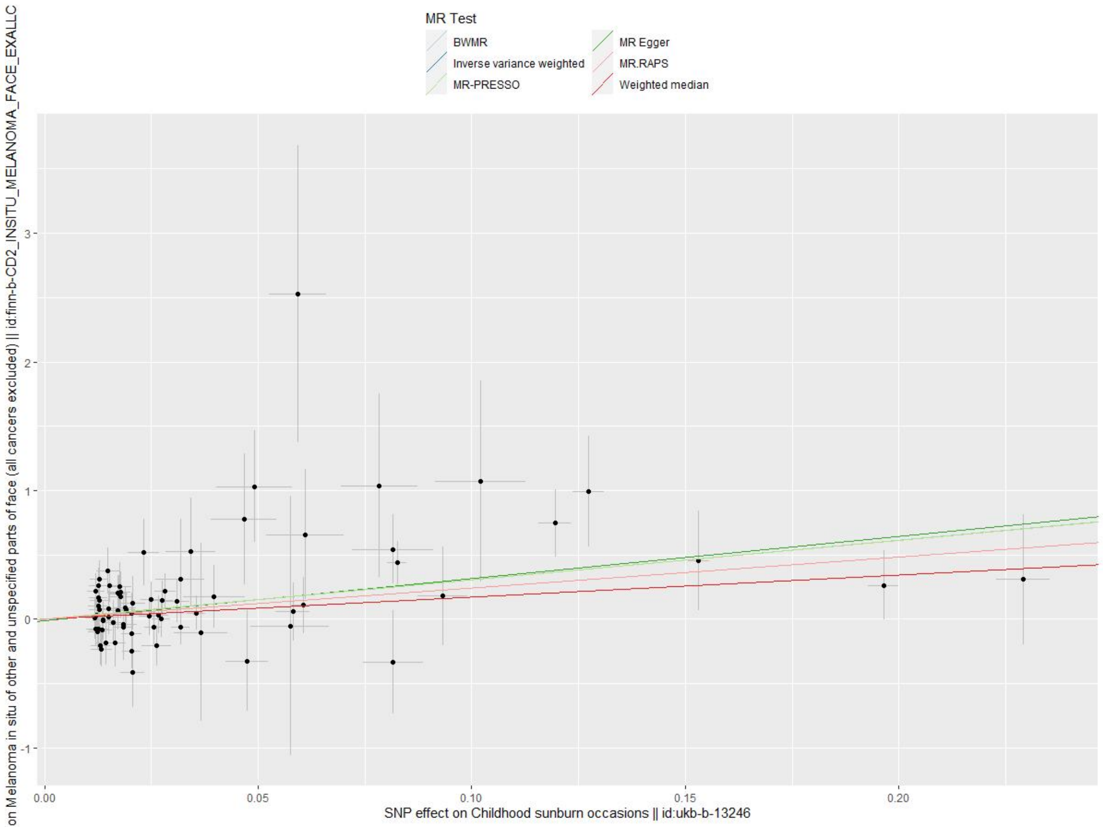

Supplementary Figure 14. Scatter plots of the replicate univariable mendelian randomization analysis of childhood sunburn and melanoma in situ of face (controls excluding all cancers). The slope of each line corresponding to the estimated MR effect in different models, including the conventional IVW, MBE, WMM, MR-Egger, MR-RAPS and MR-PRESSO methods.

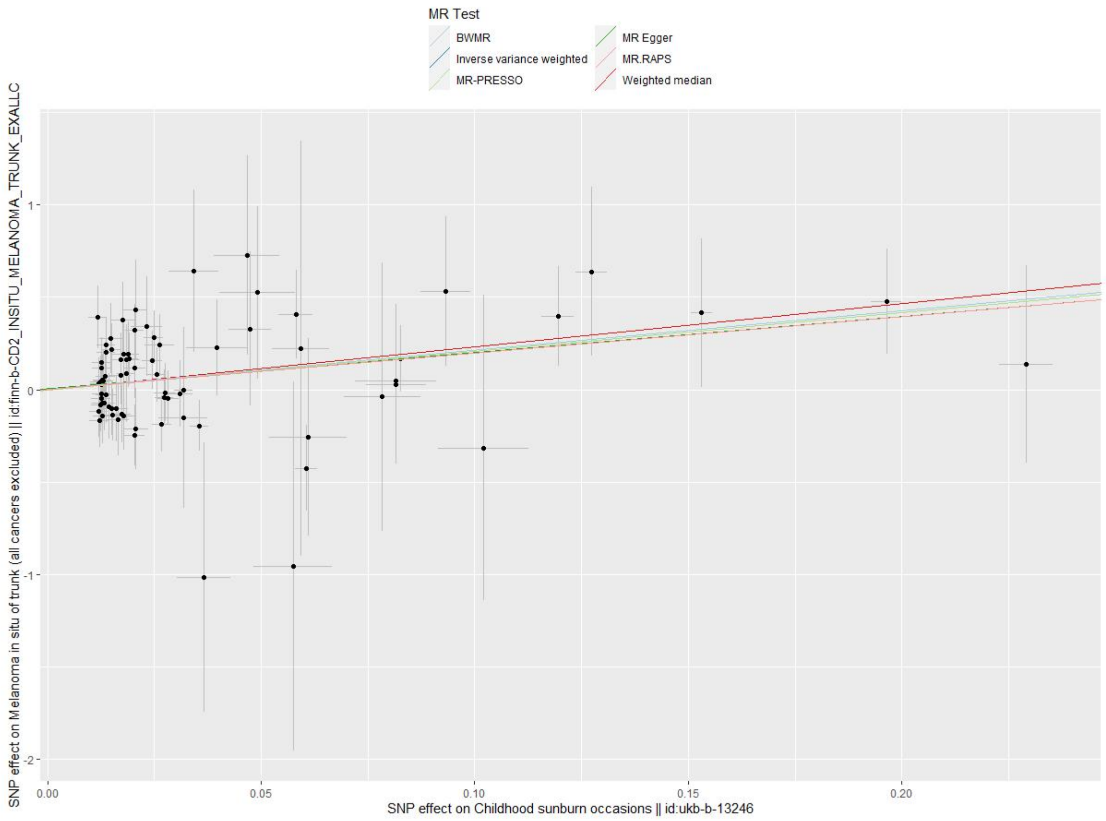

Supplementary Figure 15. Scatter plots of the replicate univariable mendelian randomization analysis of childhood sunburn and melanoma in situ of trunk (controls excluding all cancers). The slope of each line corresponding to the estimated MR effect in different models, including the conventional IVW, MBE, WMM, MR-Egger, MR-RAPS and MR-PRESSO methods.

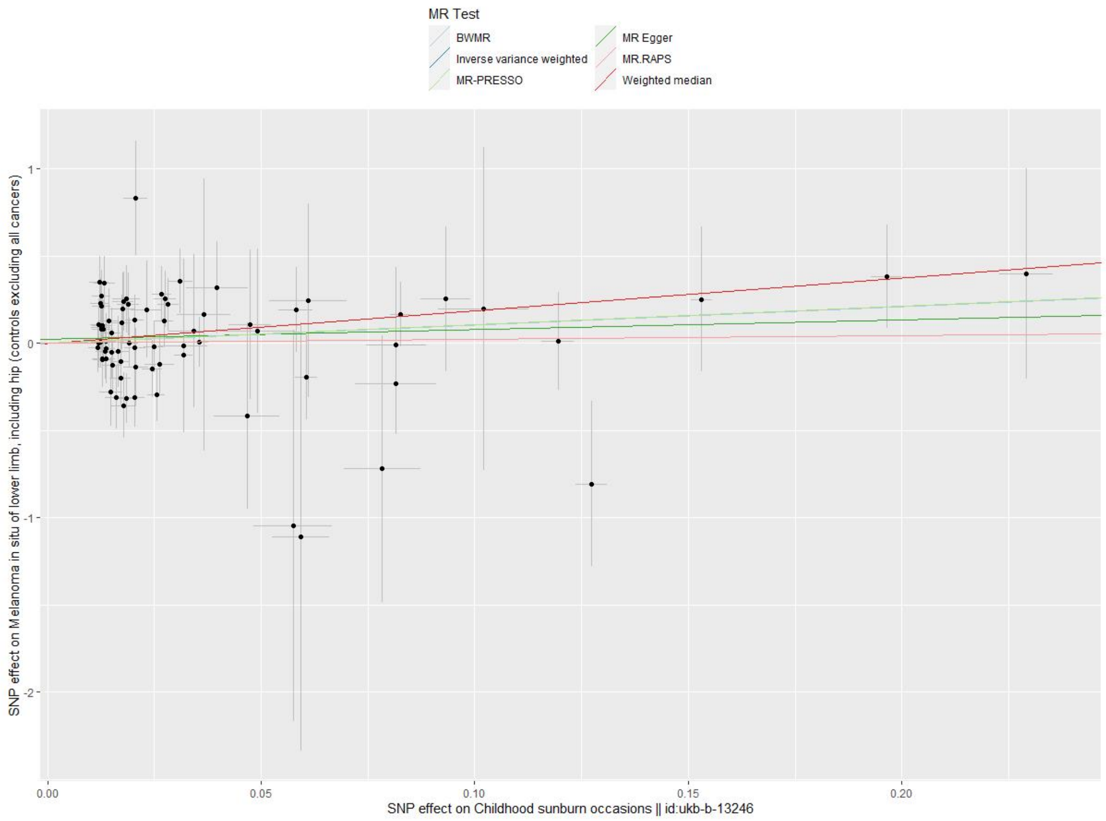

Supplementary Figure 16. Scatter plots of the replicate univariable mendelian randomization analysis of childhood sunburn and melanoma in situ of lowerlimb (controls excluding all cancers). The slope of each line corresponding to the estimated MR effect in different models, including the conventional IVW, MBE, WMM, MR-Egger, MR-RAPS and MR-PRESSO methods.

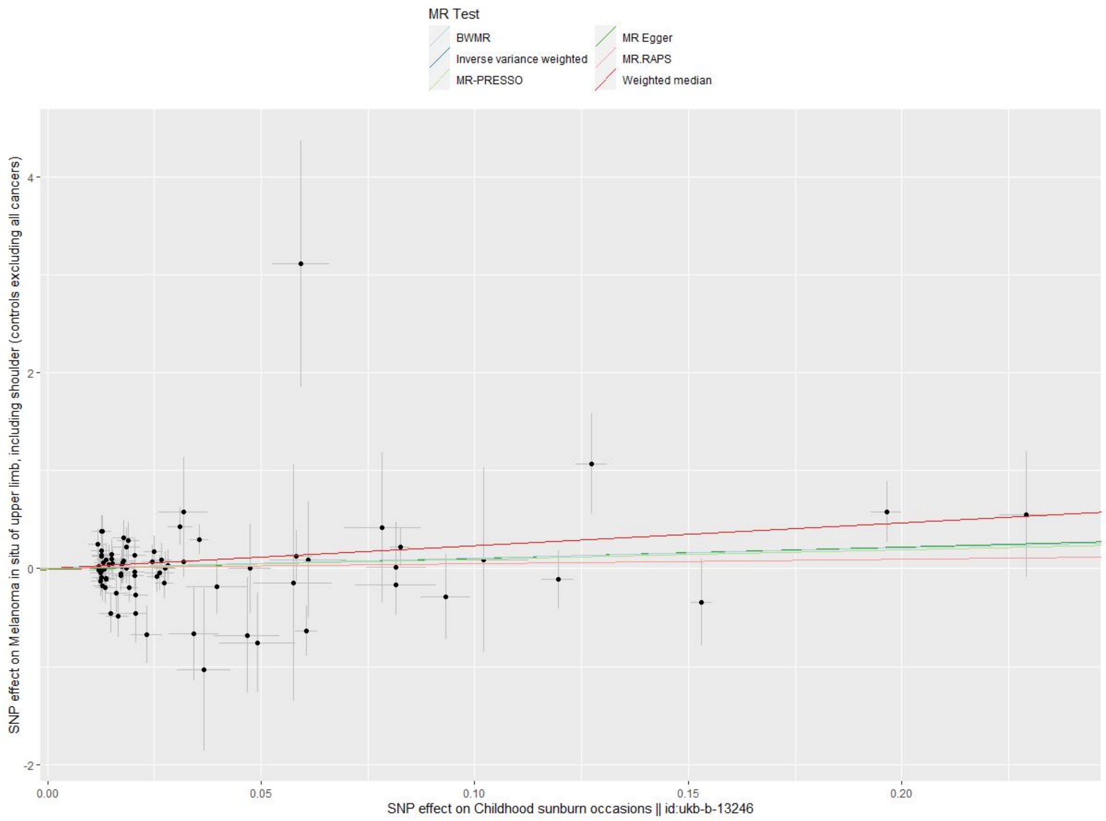

Supplementary Figure 17. Scatter plots of the replicate univariable mendelian randomization analysis of childhood sunburn and melanoma in situ of upperlimb (controls excluding all cancers). The slope of each line corresponding to the estimated MR effect in different models, including the conventional IVW, MBE, WMM, MR-Egger, MR-RAPS and MR-PRESSO methods.

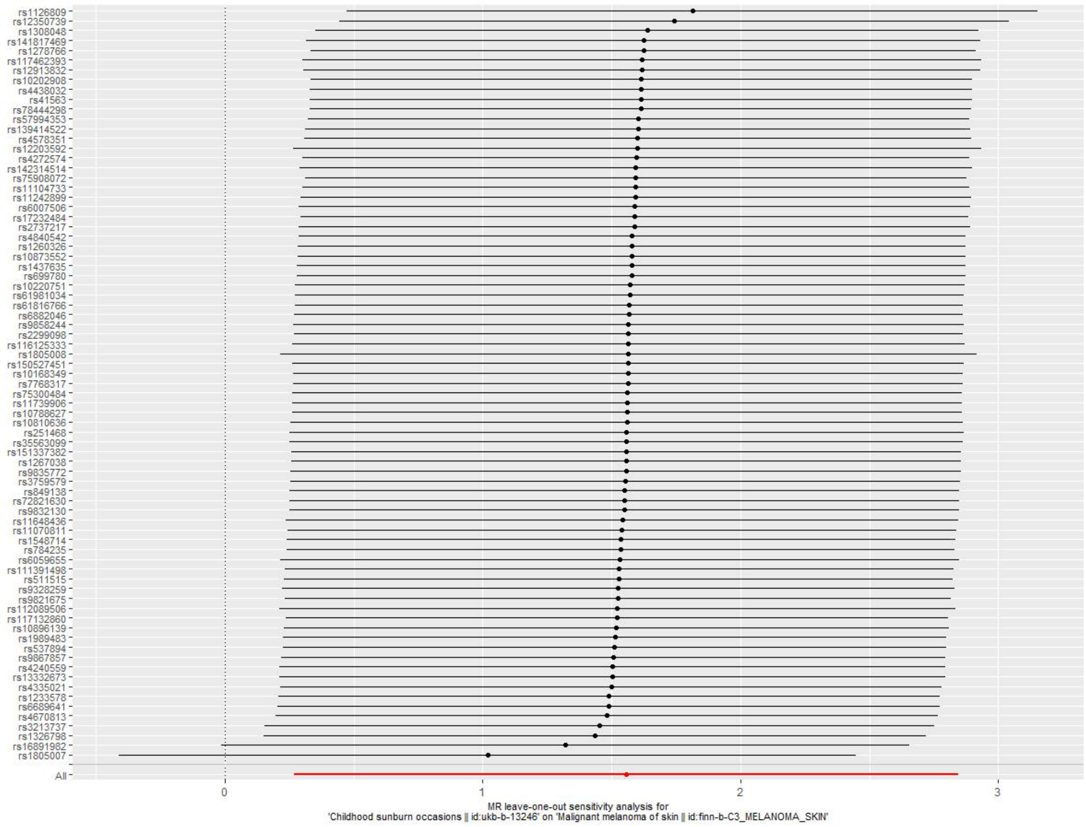

Supplementary Figure 18. Leave-one-out stability tests of the univariable mendelian randomization analysis of childhood sunburn and malignant melanoma of skin. Calculate the MR results of the remaining IVs after removing the IVs one by one.

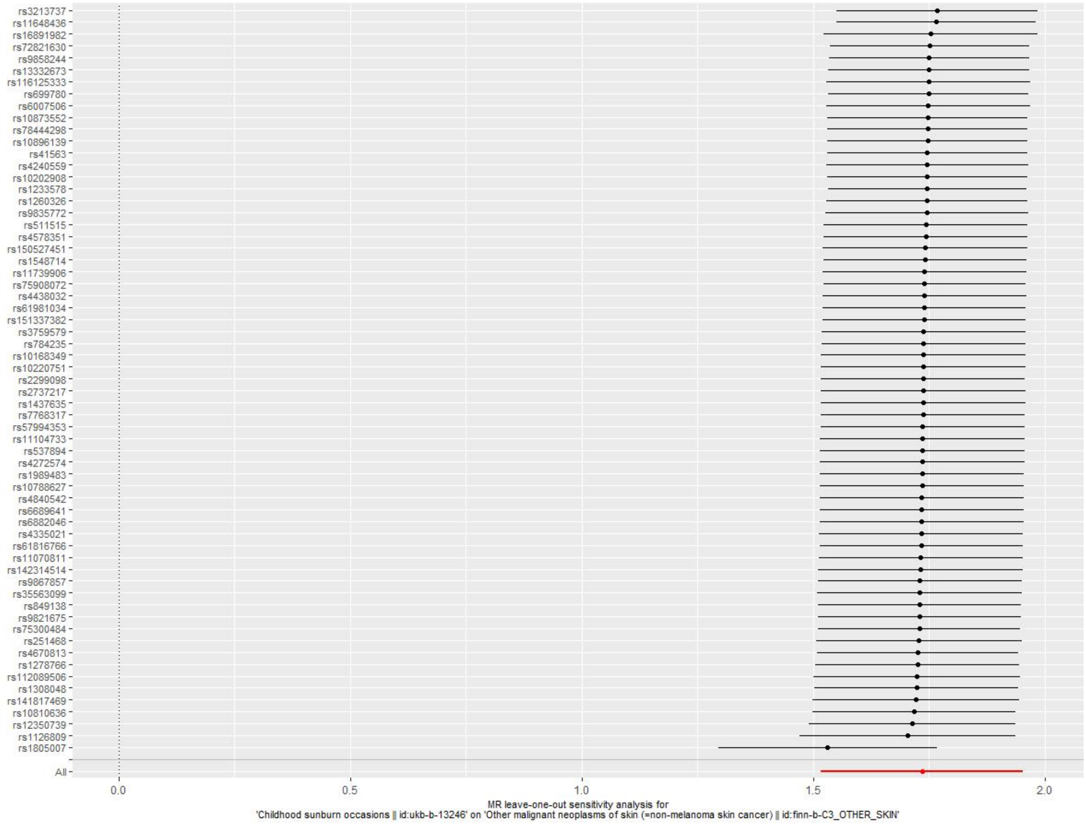

Supplementary Figure 19. Leave-one-out stability tests of the univariable mendelian randomization analysis of childhood sunburn and non-melanoma skin cancer. Calculate the MR results of the remaining IVs after removing the IVs one by one.

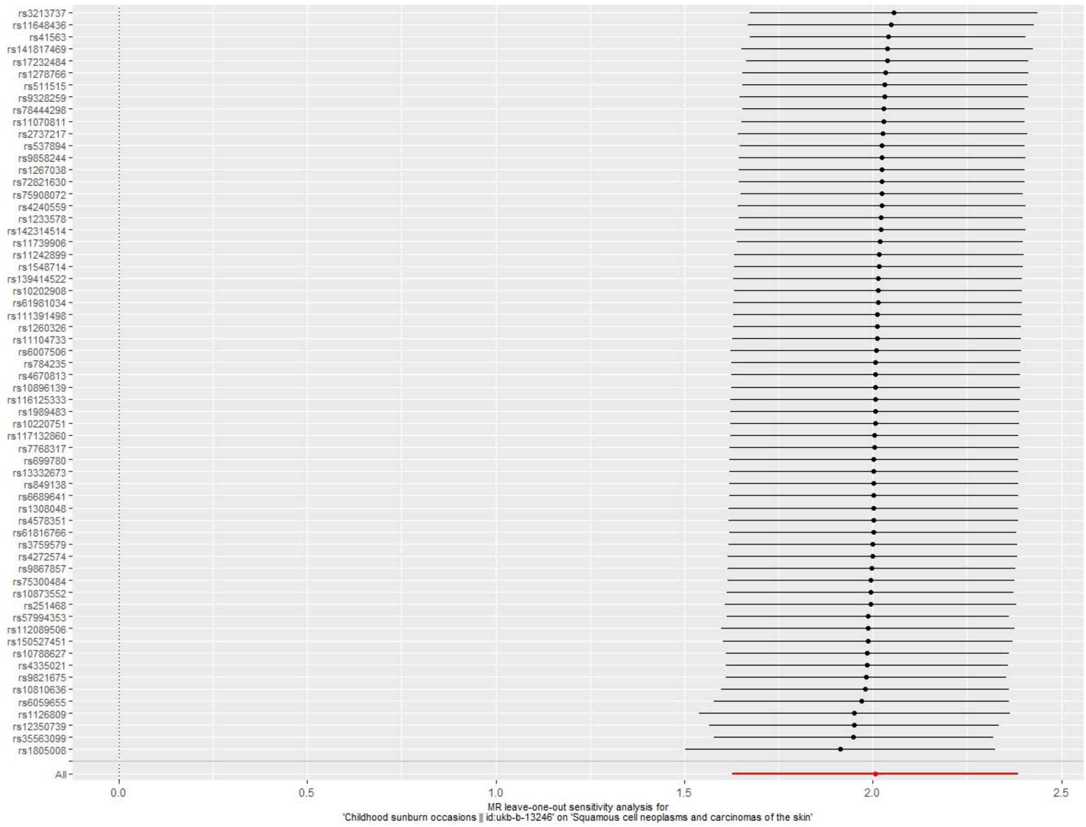

Supplementary Figure 20. Leave-one-out stability tests of the univariable mendelian randomization analysis of childhood sunburn and squamous cell carcinomas of the skin. Calculate the MR results of the remaining IVs after removing the IVs one by one.

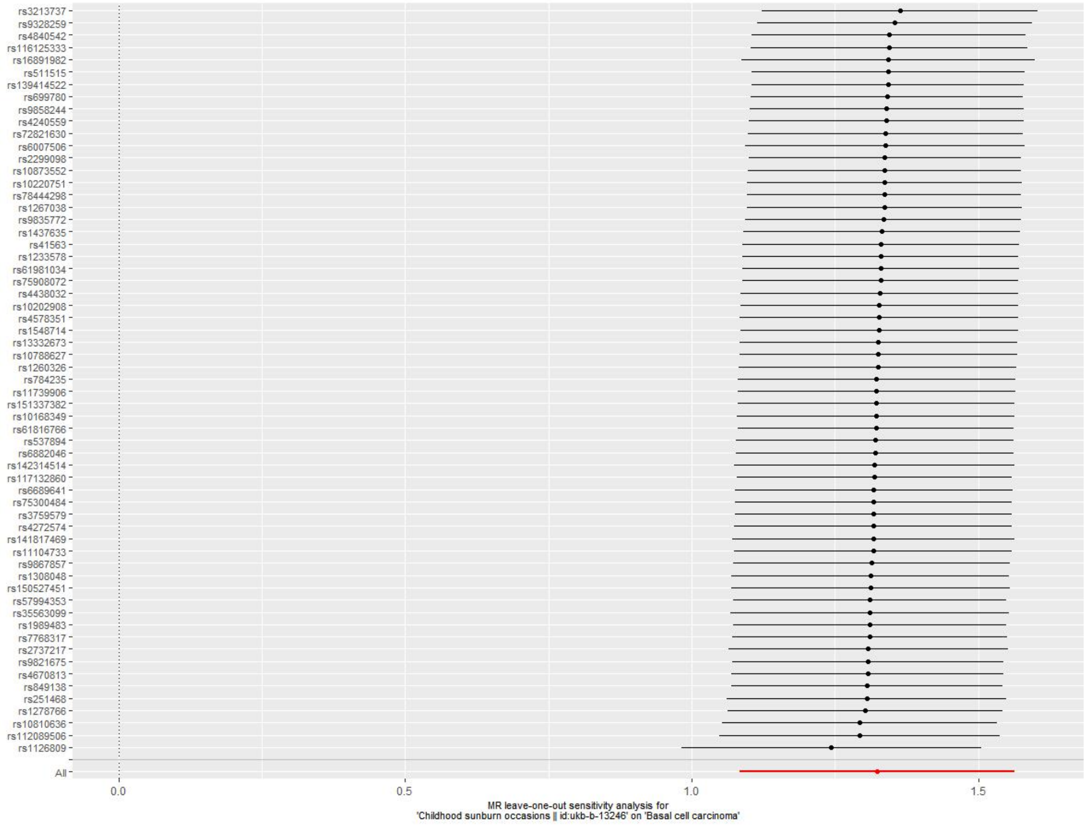

Supplementary Figure 21. Leave-one-out stability tests of the univariable mendelian randomization analysis of childhood sunburn and basal cell carcinomas of the skin. Calculate the MR results of the remaining IVs after removing the IVs one by one.

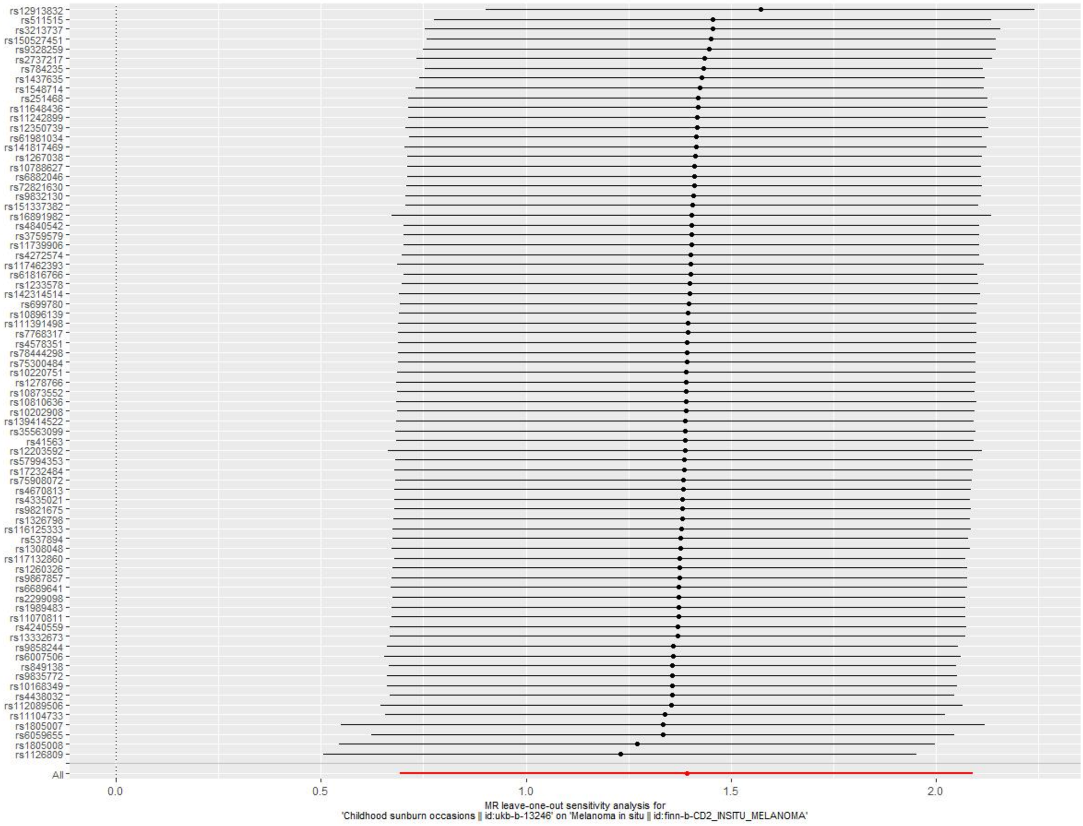

Supplementary Figure 22. Leave-one-out stability tests of the univariable mendelian randomization analysis of childhood sunburn and melanoma in situ. Calculate the MR results of the remaining IVs after removing the IVs one by one.

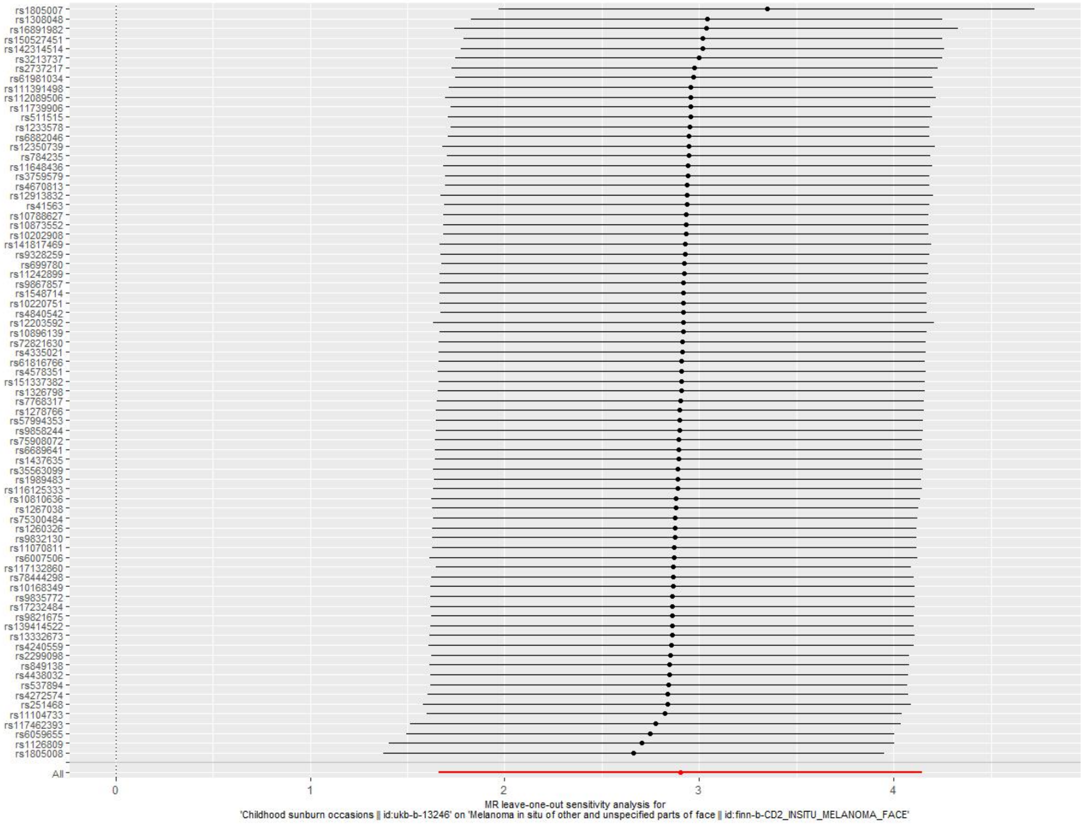

Supplementary Figure 23. Leave-one-out stability tests of the univariable mendelian randomization analysis of childhood sunburn and melanoma in situ of face. Calculate the MR results of the remaining IVs after removing the IVs one by one.

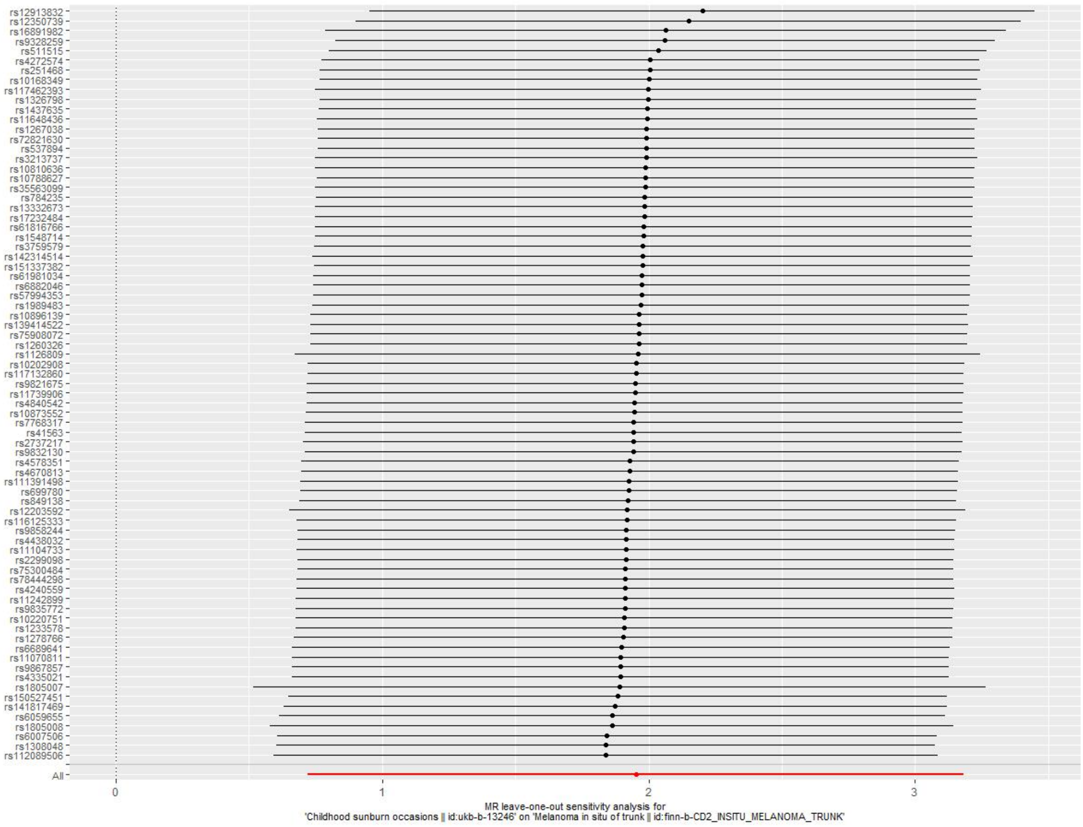

Supplementary Figure 24. Leave-one-out stability tests of the univariable mendelian randomization analysis of childhood sunburn and melanoma in situ of trunk. Calculate the MR results of the remaining IVs after removing the IVs one by one.

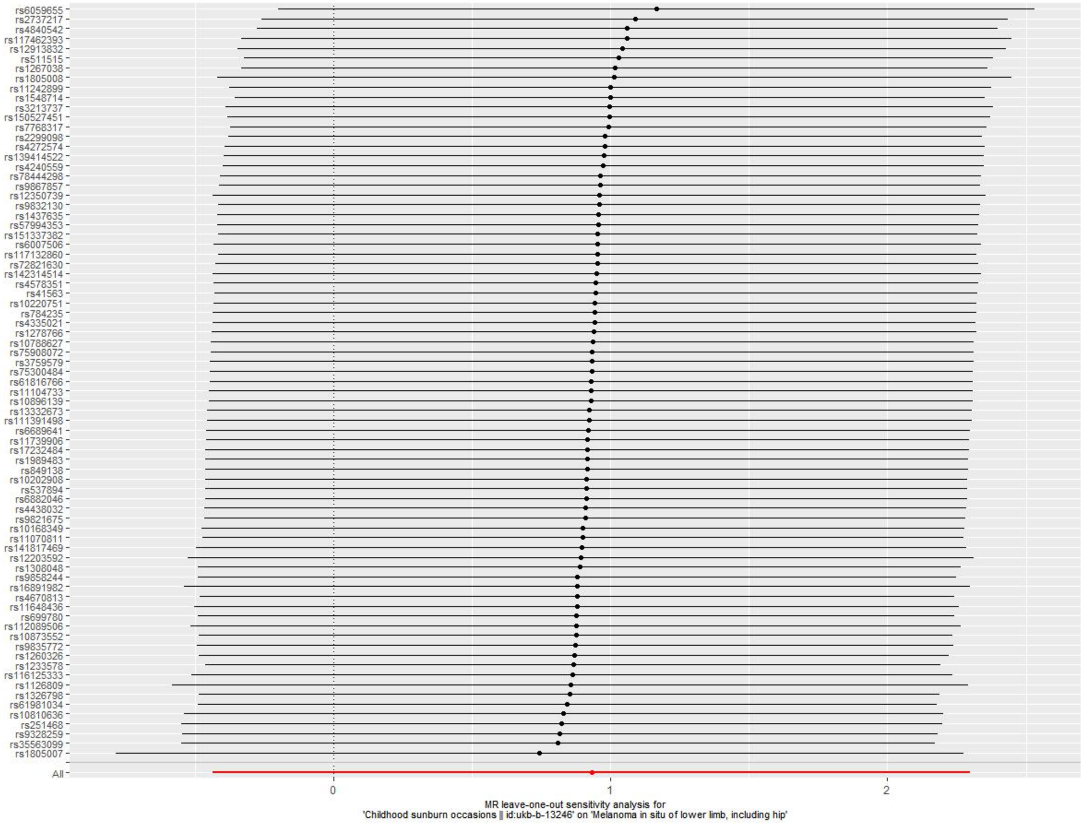

Supplementary Figure 25. Leave-one-out stability tests of the univariable mendelian randomization analysis of childhood sunburn and melanoma in situ of lowerlimb. Calculate the MR results of the remaining IVs after removing the IVs one by one.

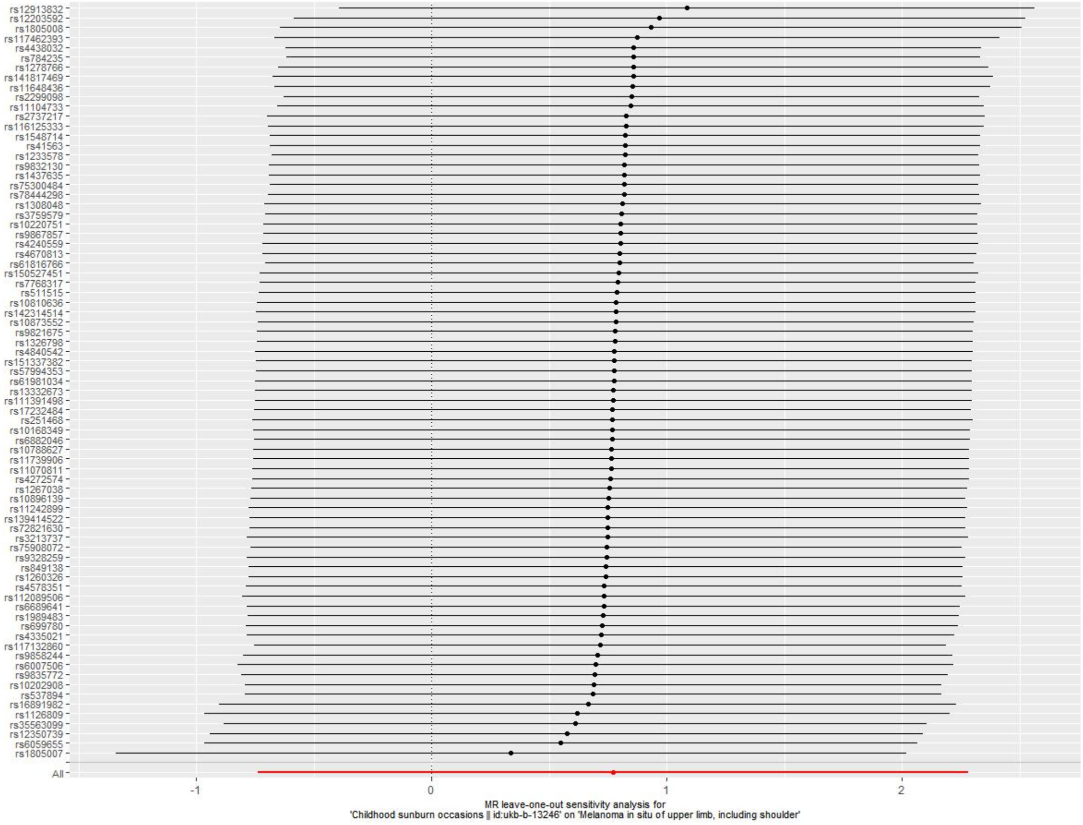

Supplementary Figure 26. Leave-one-out stability tests of the univariable mendelian randomization analysis of childhood sunburn and melanoma in situ of upperlimb. Calculate the MR results of the remaining IVs after removing the IVs one by one.

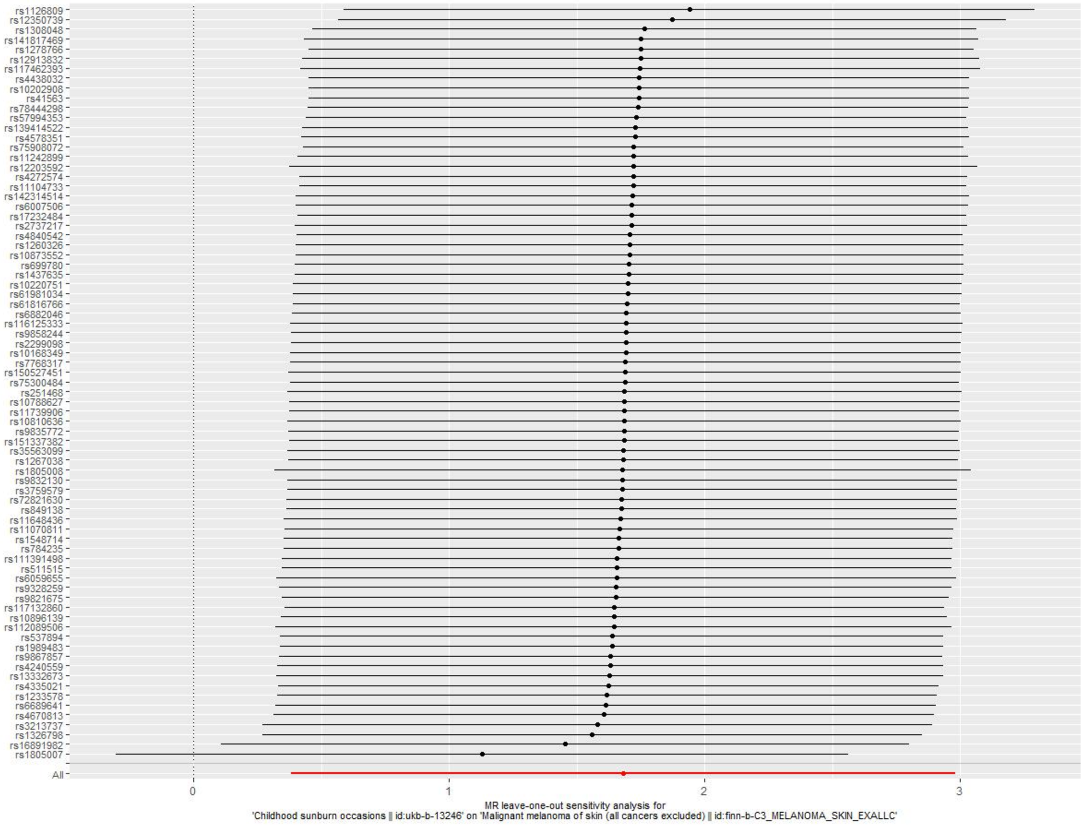

Supplementary Figure 27. Leave-one-out stability tests of the replicate univariable mendelian randomization analysis of childhood sunburn and malignant melanoma of skin (controls excluding all cancers). Calculate the MR results of the remaining IVs after removing the IVs one by one.

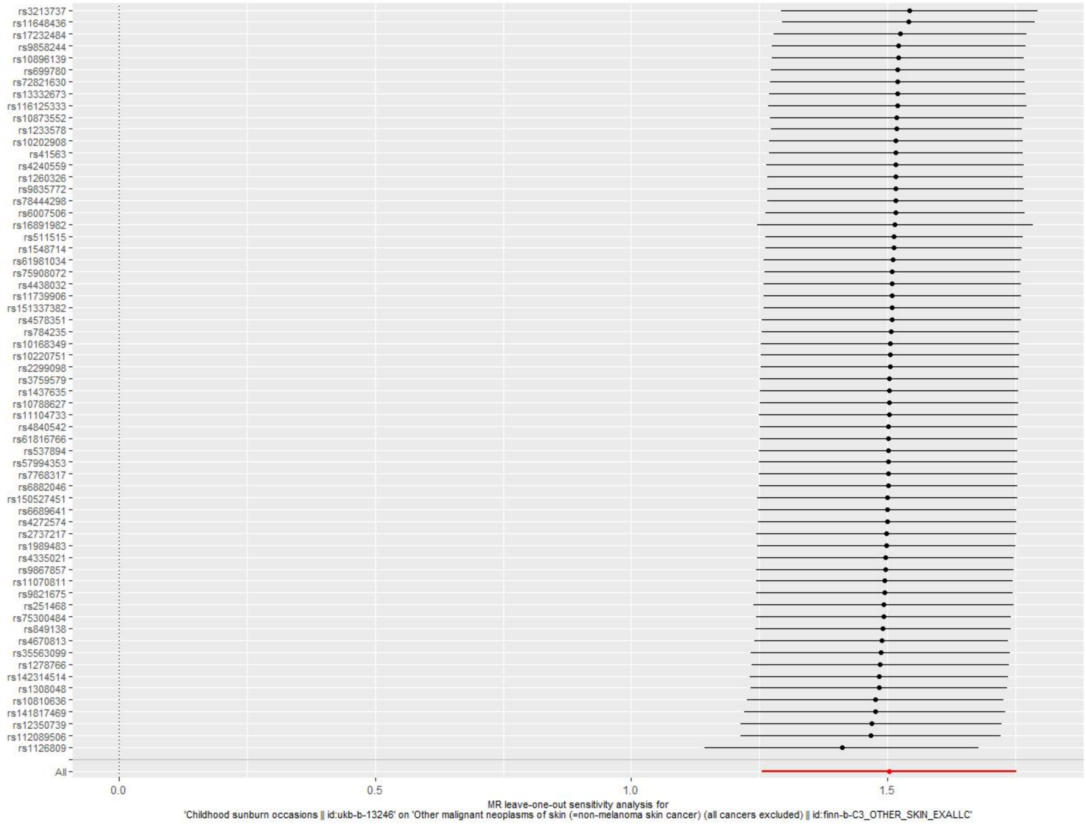

Supplementary Figure 28. Leave-one-out stability tests of the replicate univariable mendelian randomization analysis of childhood sunburn and non-melanoma skin cancer (controls excluding all cancers). Calculate the MR results of the remaining IVs after removing the IVs one by one.

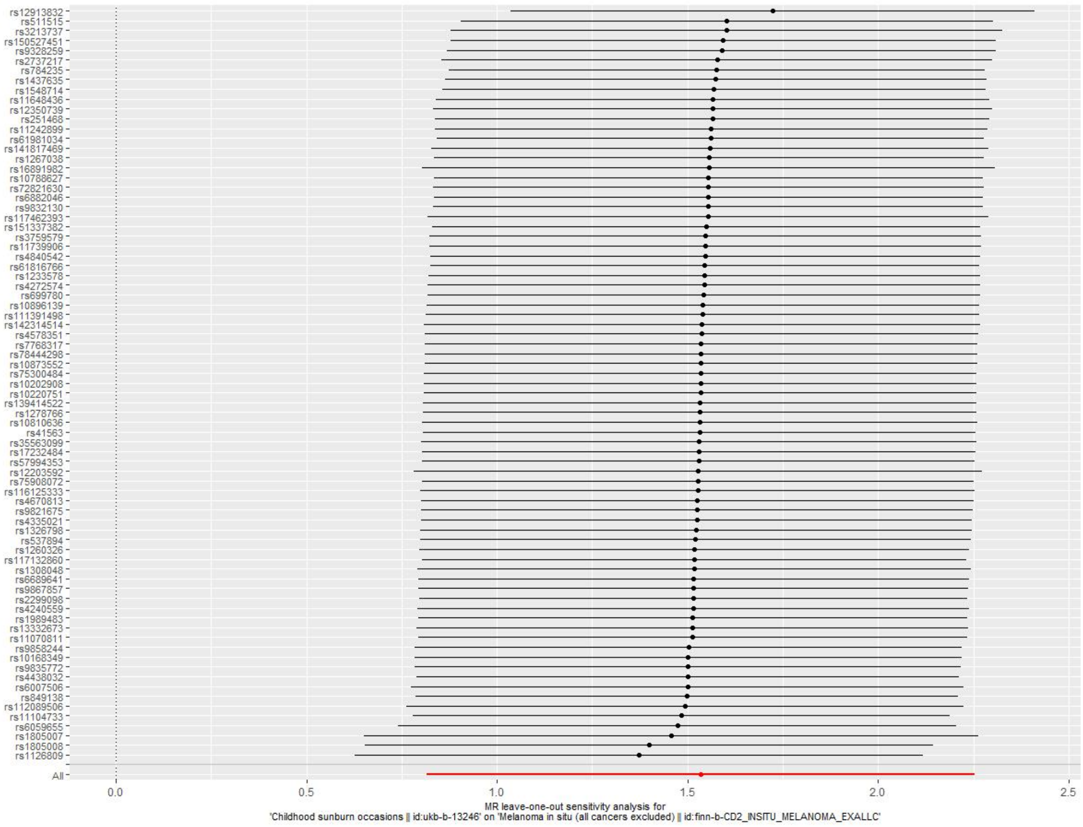

Supplementary Figure 29. Leave-one-out stability tests of the replicate univariable mendelian randomization analysis of childhood sunburn and melanoma in situ (controls excluding all cancers). Calculate the MR results of the remaining IVs after removing the IVs one by one.

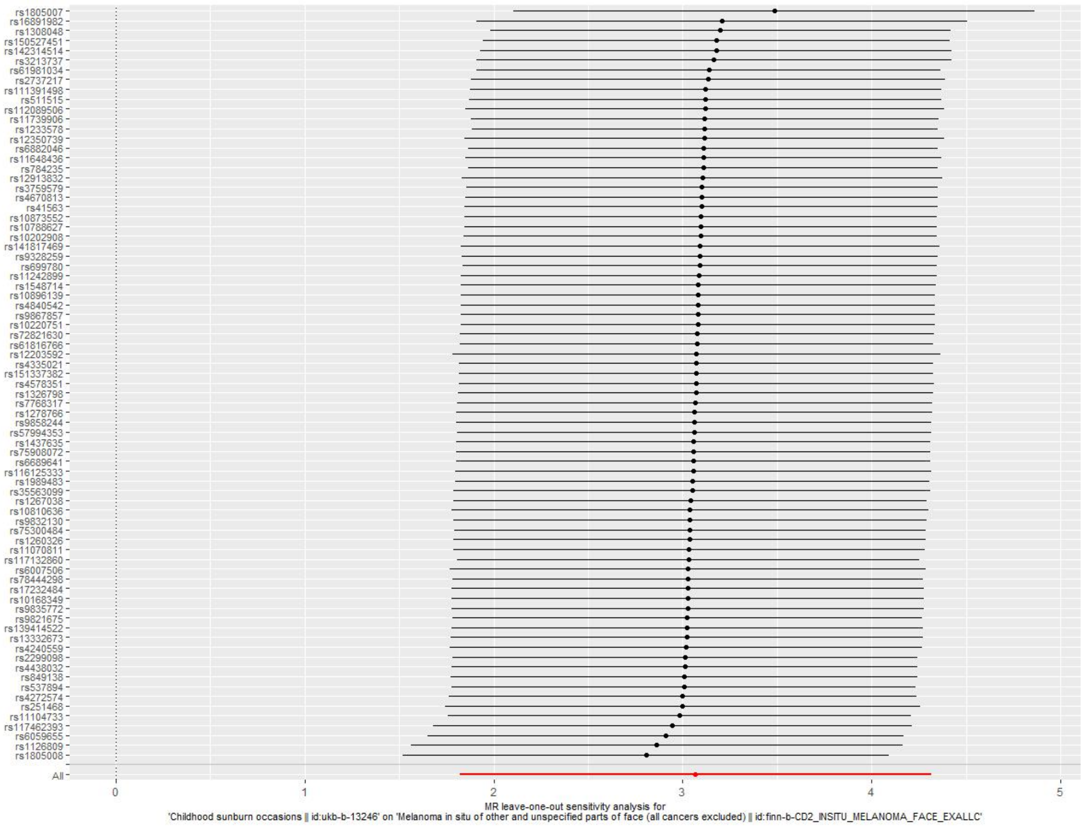

Supplementary Figure 30. Leave-one-out stability tests of the replicate univariable mendelian randomization analysis of childhood sunburn and melanoma in situ of face (controls excluding all cancers). Calculate the MR results of the remaining IVs after removing the IVs one by one.

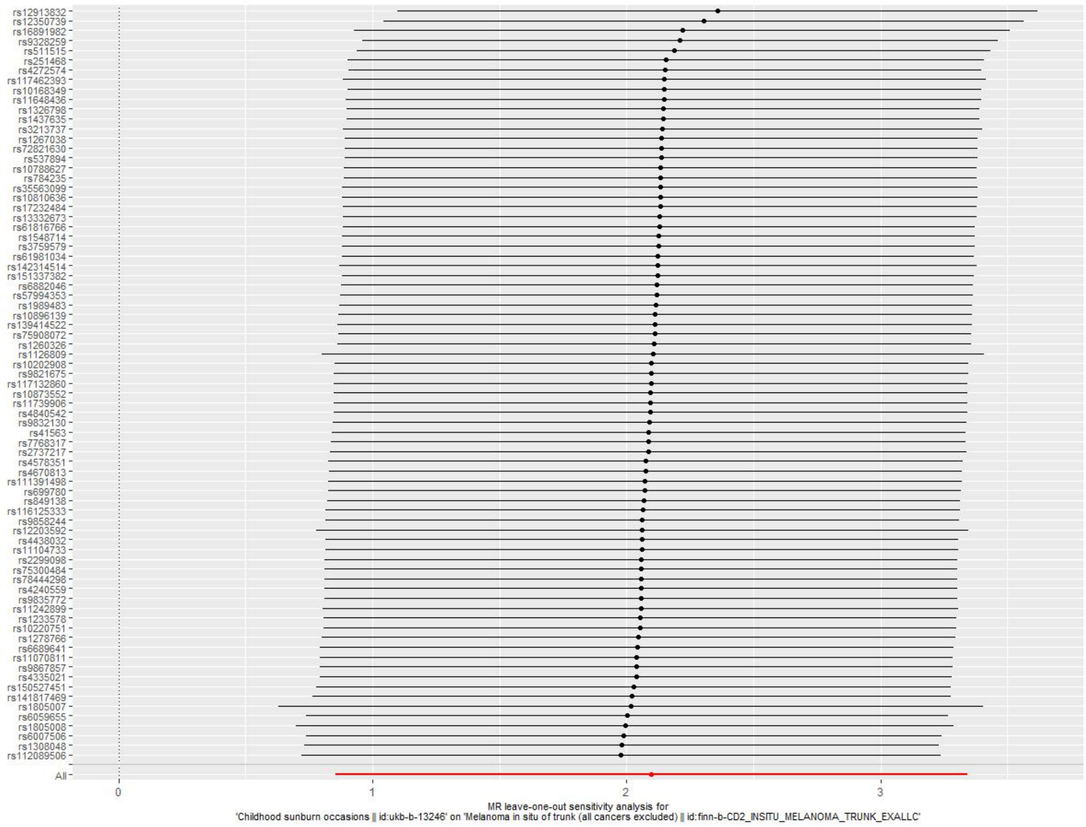

Supplementary Figure 31. Leave-one-out stability tests of the replicate univariable mendelian randomization analysis of childhood sunburn and melanoma in situ of trunk (controls excluding all cancers). Calculate the MR results of the remaining IVs after removing the IVs one by one.

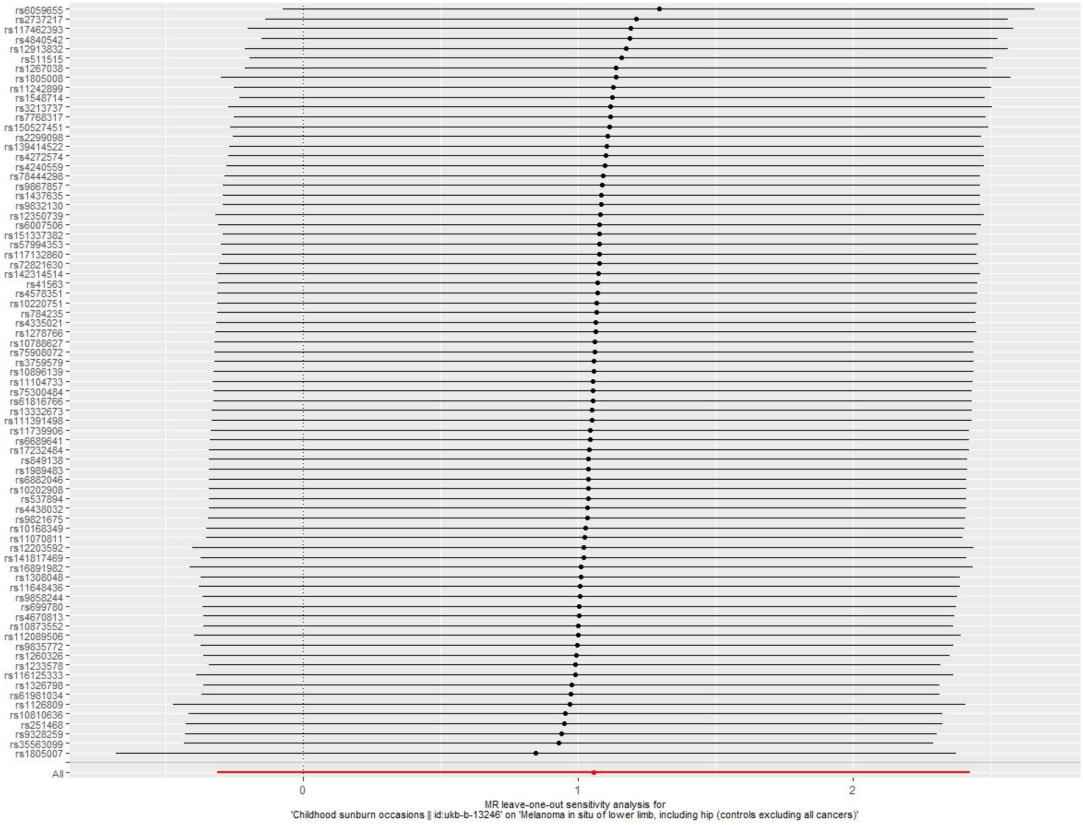

Supplementary Figure 32. Leave-one-out stability tests of the replicate univariable mendelian randomization analysis of childhood sunburn and melanoma in situ of lowerlimb (controls excluding all cancers). Calculate the MR results of the remaining IVs after removing the IVs one by one.

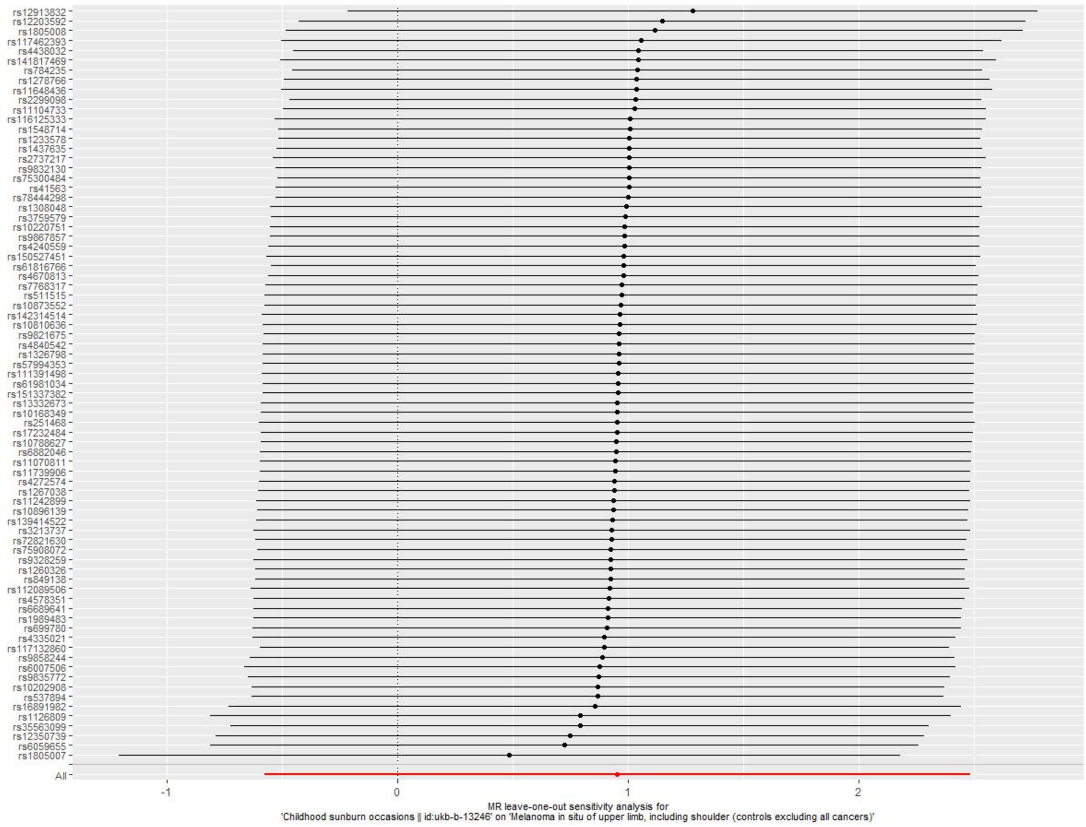

Supplementary Figure 33. Leave-one-out stability tests of the replicate univariable mendelian randomization analysis of childhood sunburn and melanoma in situ of upperlimb (controls excluding all cancers). Calculate the MR results of the remaining IVs after removing the IVs one by one.

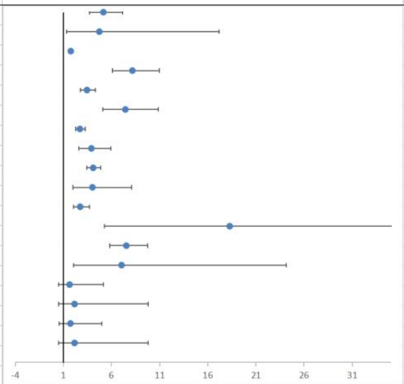

Exposure	Outcome	Method	Forest plot	OR	95% CIs	P Value	False positive error due to correlated pleiotropy
Childhood sunburn	Malignant melanoma of skin	CAUSE		5.1552	3.7062 to 7.1707	2.80E-04	No
		Inverse variance weighted		4.7387	1.3067 to 17.1853	1.79E-02	
	Non-melanoma skin cancer	CAUSE		1.7683	1.6323 to 1.9348	1.20E-04	No
		MR Egger		8.1694	6.0729 to 10.9896	1.53E-20	
	Squamous cell carcinomas of the skin	CAUSE		3.4556	2.7732 to 4.3492	3.70E-02	No
		Inverse variance weighted		7.4383	5.0923 to 10.8651	3.07E-25	
	Basal cell carcinomas of the skin	CAUSE		2.7183	2.2705 to 3.2871	1.30E-02	No
		MR Egger		3.9117	2.5863 to 5.9164	2.19E-08	
	Melanoma in situ	CAUSE		4.0960	3.4556 to 4.9037	3.70E-04	No
		Inverse variance weighted		4.0207	2.0000 to 8.0830	9.40E-05	
	Melanoma in situ of face	CAUSE		2.7456	2.0340 to 3.7062	1.10E-02	No
		Inverse variance weighted		18.2808	5.2753 to 63.3493	4.59E-06	
	Melanoma in situ of trunk	CAUSE		7.5383	5.8124 to 9.7767	1.70E-10	No
		Inverse variance weighted		7.0469	2.0579 to 24.1300	1.88E-03	
	Melanoma in situ of lowerlimb	CAUSE		1.6487	0.5273 to 5.1552	7.90E-01	No
		Inverse variance weighted		2.1631	0.4781 to 9.7874	3.16E-01	
	Melanoma in situ of upperlimb	CAUSE		1.7333	0.5655 to 5.0028	6.30E-01	No
		Inverse variance weighted		2.1631	0.4781 to 9.7874	3.16E-01	

Supplementary Figure 34. The forest plot of the CAUSE method MR analysis. Causal estimates were given as beta and 95% confidence intervals (CIs).

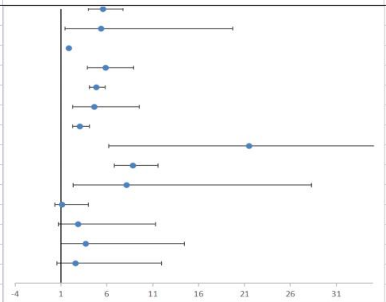

Exposure	Outcome	Method	Forest plot	OR	95% CIs	P Value	False positive error due to correlated pleiotropy
Childhood sunburn	Malignant melanoma of skin (controls excluding all cancers)	CAUSE		5.584528464	4.0149 to 7.7679	0.00039	No
		Inverse variance weighted		5.378743415	1.4657 to 19.7389	0.0112012	
	Non-melanoma skin cancer (controls excluding all cancers)	CAUSE		1.858928042	1.6989 to 2.0544	0.0046	No
		MR Egger		5.873892361	3.8641 to 8.9290	1.42E-11	
	Melanoma in situ (controls excluding all cancers)	CAUSE		4.854955811	4.0960 to 5.8124	0.00035	No
		Inverse variance weighted		4.639775424	2.2606 to 9.5229	0.0000287	
	Melanoma in situ of face (controls excluding all cancers)	CAUSE		3.064854203	2.2933 to 4.0960	0.00059	No
		MR Egger		21.50628371	6.1860 to 74.7689	1.39E-06	
	Melanoma in situ of trunk (controls excluding all cancers)	CAUSE		8.846306259	6.8210 to 11.5883	4.3E-12	No
		Inverse variance weighted		8.154444989	2.3501 to 28.2942	0.0009458	
	Melanoma in situ of lowerlimb (controls excluding all cancers)	CAUSE		1.127496852	0.3198 to 4.0149	0.97	No
		Inverse variance weighted		2.873388186	0.7302 to 11.3067	0.1310038	
	Melanoma in situ of upperlimb (controls excluding all cancers)	CAUSE		3.706173712	0.9802 to 14.4400	0.3	No
		Inverse variance weighted		2.593041399	0.5619 to 11.9669	0.2220219	

Supplementary Figure 35. The forest plot of the replicate CAUSE method MR analysis (controls excluding all cancers). Causal estimates were given as beta and 95% confidence intervals (CIs).

Exposure	Mediator		Forest plots	OR	95% CIs	P-value	Proportion of mediation	Outcome
Childhood sunburn	Total effect		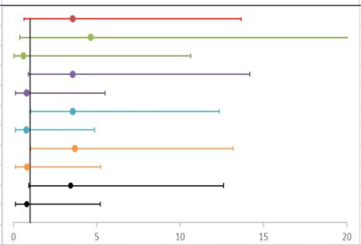	3.558	0.6260 to 13.6713	6.46E-02		Malignant melanoma of skin
	Skin colour	Direct effect		4.635	0.3688 to 58.2590	2.35E-01	39.60%	
		Meditation effect		0.605	0.0344 to 10.6372	7.31E-01		
	Serum vitamin D levels	Direct effect		3.557	0.8915 to 14.1914	7.23E-02	18.02%	
		Meditation effect		0.796	0.1154 to 5.4835	8.16E-01		
	Body mass index	Direct effect		3.559	1.0256 to 12.3523	4.55E-02	19.87%	
		Meditation effect		0.777	0.1243 to 4.8591	7.87E-01		
	Alcohol intake	Direct effect		3.691	1.0339 to 13.1786	4.43E-02	15.66%	
		Meditation effect		0.820	0.1286 to 5.2259	8.33E-01		
	Smoking	Direct effect		3.434	0.9354 to 12.6100	6.30E-02	17.33%	
		Meditation effect		0.803	0.1235 to 5.2164	8.18E-01		
Childhood sunburn	Total effect		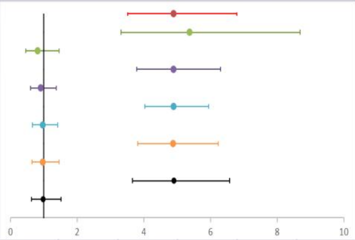	4.893	3.5252 to 6.7914	2.26E-21		Non-melanoma skin cancer
	Skin colour	Direct effect		5.374	3.3235 to 8.6884	6.93E-12	12.72%	
		Meditation effect		0.817	0.4567 to 1.4618	4.96E-01		
	Serum vitamin D levels	Direct effect		4.892	3.7920 to 6.3102	2.43E-34	6.00%	
		Meditation effect		0.909	0.6002 to 1.3769	6.53E-01		
	Body mass index	Direct effect		4.893	4.0254 to 5.9486	3.42E-57	1.81%	
		Meditation effect		0.972	0.6634 to 1.4231	8.83E-01		
	Alcohol intake	Direct effect		4.879	3.8230 to 6.2262	3.61E-37	1.88%	
		Meditation effect		0.971	0.6450 to 1.4605	8.86E-01		
	Smoking	Direct effect		4.904	3.6611 to 6.5693	1.54E-26	1.11%	
		Meditation effect		0.983	0.6333 to 1.5245	9.37E-01		
Childhood sunburn	Total effect		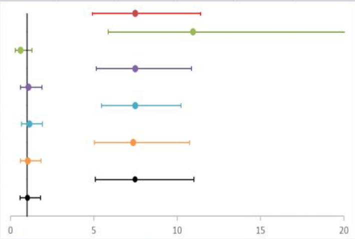	7.490	4.9176 to 11.4084	6.61E-21		Squamous cell carcinomas of the skin
	Skin colour	Direct effect		10.955	5.8619 to 20.4716	6.22E-14	24.30%	
		Meditation effect		0.613	0.2885 to 1.3026	2.03E-01		
	Serum vitamin D levels	Direct effect		7.490	5.1618 to 10.8688	2.97E-26	3.95%	
		Meditation effect		1.083	0.6173 to 1.8990	7.82E-01		
	Body mass index	Direct effect		7.490	5.4791 to 10.2380	1.53E-36	6.55%	
		Meditation effect		1.141	0.6756 to 1.9273	6.22E-01		
	Alcohol intake	Direct effect		7.366	5.0437 to 10.7580	4.97E-25	2.30%	
		Meditation effect		1.047	0.5946 to 1.8449	8.73E-01		
	Smoking	Direct effect		7.477	5.0801 to 11.0054	1.95E-24	1.29%	
		Meditation effect		1.026	0.5797 to 1.8174	9.29E-01		
Childhood sunburn	Total effect		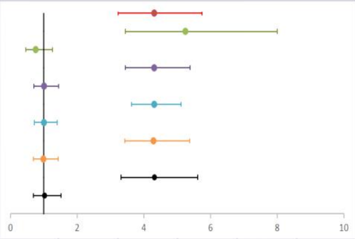	4.313	3.2346 to 5.7522	2.43E-23		Basal cell carcinomas of the skin
	Skin colour	Direct effect		5.248	3.4408 to 8.0054	1.40E-14	18.89%	
		Meditation effect		0.759	0.4551 to 1.2647	2.90E-01		
	Serum vitamin D levels	Direct effect		4.312	3.4503 to 5.3900	9.43E-38	0.58%	
		Meditation effect		1.009	0.7008 to 1.4516	9.63E-01		
	Body mass index	Direct effect		4.314	3.6373 to 5.1158	5.84E-03	0.40%	
		Meditation effect		1.006	0.7198 to 1.4055	9.73E-01		
	Alcohol intake	Direct effect		4.292	3.4272 to 5.3740	6.37E-37	0.71%	
		Meditation effect		0.990	0.6869 to 1.4261	9.56E-01		
	Smoking	Direct effect		4.321	3.3236 to 5.6168	8.02E-28	1.70%	
		Meditation effect		1.025	0.6945 to 1.5134	9.00E-01		
Childhood sunburn	Total effect		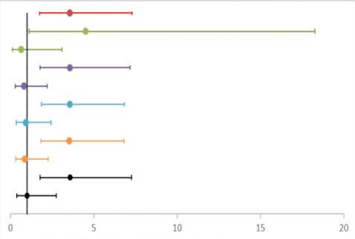	3.564	1.7412 to 7.2945	5.06E-04		Melanoma in situ
	Skin colour	Direct effect		4.511	1.1145 to 18.2538	3.47E-02	34.43%	
		Meditation effect		0.646	0.1342 to 3.1058	5.85E-01		
	Serum vitamin D levels	Direct effect		3.569	1.7732 to 7.1842	3.64E-04	16.15%	
		Meditation effect		0.814	0.2992 to 2.2164	6.88E-01		
	Body mass index	Direct effect		3.565	1.8576 to 6.8402	1.32E-04	6.34%	
		Meditation effect		0.923	0.3503 to 2.4297	8.70E-01		
	Alcohol intake	Direct effect		3.531	1.8307 to 6.8122	1.67E-04	11.97%	
		Meditation effect		0.859	0.3249 to 2.2701	7.59E-01		
	Smoking	Direct effect		3.582	1.7655 to 7.2689	4.08E-04	0.13%	
		Meditation effect		1.002	0.3660 to 2.7416	9.97E-01		
Childhood sunburn	Total effect		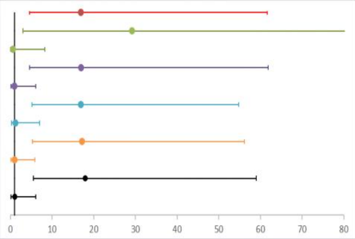	16.905	4.6416 to 61.5694	1.80E-05		Melanoma in situ of face
	Skin colour	Direct effect		29.179	3.0350 to 280.5275	3.48E-03	17.50%	
		Meditation effect		0.610	0.0450 to 8.2604	7.10E-01		
	Serum vitamin D levels	Direct effect		16.959	4.6509 to 61.8367	1.80E-05	0.93%	
		Meditation effect		0.974	0.1565 to 6.0653	9.78E-01		
	Body mass index	Direct effect		16.909	5.2231 to 54.7382	2.38E-06	6.88%	
		Meditation effect		1.215	0.2118 to 6.9667	8.27E-01		
	Alcohol intake	Direct effect		17.184	5.2619 to 56.1200	2.48E-06	0.36%	
		Meditation effect		1.010	0.1751 to 5.8287	9.91E-01		
	Smoking	Direct effect		17.955	5.4674 to 58.9649	1.93E-06	2.04%	
		Meditation effect		1.059	0.1829 to 6.1346	9.49E-01		
Childhood sunburn	Total effect		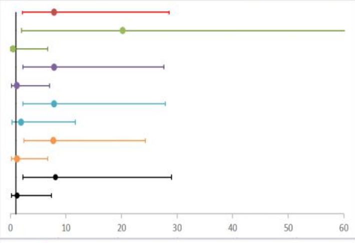	7.860	2.1599 to 28.6018	1.76E-03		Melanoma in situ of trunk
	Skin colour	Direct effect		20.201	2.0386 to 200.1828	1.02E-02	35.50%	
		Meditation effect		0.481	0.0346 to 6.6873	5.86E-01		
	Serum vitamin D levels	Direct effect		7.861	2.2396 to 27.5913	1.29E-03	7.22%	
		Meditation effect		1.161	0.1916 to 7.0307	8.71E-01		
	Body mass index	Direct effect		7.862	2.2194 to 27.8509	1.40E-03	31.62%	
		Meditation effect		1.919	0.3147 to 11.7009	4.80E-01		
	Alcohol intake	Direct effect		7.726	2.4602 to 24.2597	4.62E-04	8.59%	
		Meditation effect		1.194	0.2126 to 6.7043	8.41E-01		
	Smoking	Direct effect		8.115	2.2729 to 28.9748	1.26E-03	9.15%	
		Meditation effect		1.208	0.1970 to 7.4046	8.38E-01		
Childhood sunburn	Total effect		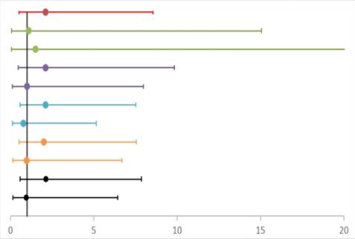	2.111	0.5220 to 8.5382	2.95E-01		Melanoma in situ of lowerlimb
	Skin colour	Direct effect		1.097	0.0800 to 15.0541	9.45E-01	54.66%	
		Meditation effect		1.504	0.0773 to 29.2786	7.87E-01		
	Serum vitamin D levels	Direct effect		2.112	0.4537 to 9.8329	3.41E-01	0.14%	
		Meditation effect		0.999	0.1251 to 7.9795	9.99E-01		
	Body mass index	Direct effect		2.111	0.5928 to 7.5204	2.49E-01	33.46%	
		Meditation effect		0.779	0.1178 to 5.1467	7.95E-01		
	Alcohol intake	Direct effect		2.007	0.5330 to 7.5571	3.03E-01	3.15%	
		Meditation effect		0.977	0.1423 to 6.7039	9.81E-01		
	Smoking	Direct effect		2.130	0.5761 to 7.8749	2.57E-01	6.72%	
		Meditation effect		0.951	0.1403 to 6.4464	9.59E-01		
Childhood sunburn	Total effect		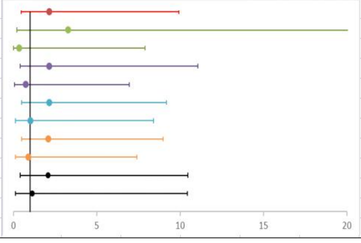	2.153	0.4670 to 9.9258	3.25E-01		Melanoma in situ of upperlimb
	Skin colour	Direct effect		3.282	0.2158 to 49.9005	3.92E-01	NA	
		Meditation effect		0.348	0.0153 to 7.8832	5.07E-01		
	Serum vitamin D levels	Direct effect		2.149	0.4173 to 11.0680	3.60E-01	39.54%	
		Meditation effect		0.738	0.0785 to 6.9431	7.91E-01		
	Body mass index	Direct effect		2.153	0.5052 to 9.1752	3.00E-01	2.79%	
		Meditation effect		1.022	0.1243 to 8.3965	9.84E-01		
	Alcohol intake	Direct effect		2.095	0.4895 to 8.9702	3.19E-01	14.18%	
		Meditation effect		0.897	0.1088 to 7.3950	9.20E-01		
	Smoking	Direct effect		2.080	0.4138 to 10.4592	3.74E-01	15.87%	
		Meditation effect		1.129	0.1222 to 10.4351	9.15E-01		

Supplementary Figure 36. The forest plot of the mediation MR analyses. Causal estimates given as odds ratio (OR) and 95% confidence intervals (CIs) for the effect of childhood sunburn and skin carcinoma risk. Red:total effect; Green: effect of skin colour; Purple: effect of serum vitamin D levels; Blue: effect of body mass index; Orange: effect of alcohol intake; Black: effect of smoking; Brown: effect of hair colour; Grey: effect of facial ageing.

Exposure	Mediator		Forest plots	OR	95% CIs	P-value	Proportion of mediation	Outcomes
Childhood sunburn	Total effect		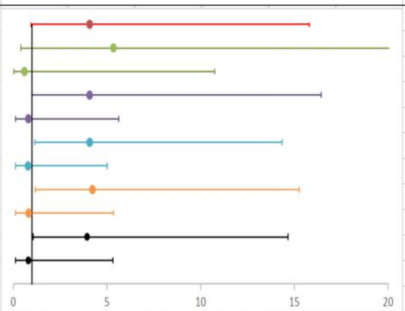	4.080	0.9629 to 15.8122	4.19E-02		Malignant melanoma of skin (controls excluding all cancers)
	Skin colour	Direct effect		5.348	0.4199 to 68.1256	1.96E-01	36.05%	
		Meditation effect		0.602	0.0337 to 10.7584	7.30E-01		
	Serum vitamin D levels	Direct effect		4.079	1.0126 to 16.4319	4.80E-02	15.14%	
		Meditation effect		0.808	0.1158 to 5.6427	8.30E-01		
	Body mass index	Direct effect		4.082	1.1621 to 14.3363	2.82E-02	16.77%	
		Meditation effect		0.790	0.1245 to 5.0114	8.02E-01		
	Alcohol intake	Direct effect		4.230	1.1715 to 15.2714	2.77E-02	13.64%	
		Meditation effect		0.825	0.1277 to 5.3365	8.40E-01		
	Smoking	Direct effect		3.943	1.0592 to 14.6752	4.08E-02	15.25%	
		Meditation effect		0.807	0.1222 to 5.3284	8.24E-01		
Childhood sunburn	Total effect		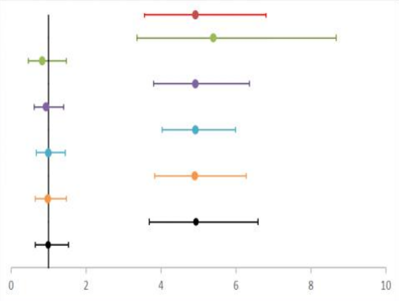	4.919	3.5572 to 6.8016	5.74E-22		Non-melanoma skin cancer (controls excluding all cancers)
	Skin colour	Direct effect		5.396	3.3583 to 8.6695	3.23E-12	11.68%	
		Meditation effect		0.830	0.4675 to 1.4745	5.25E-01		
	Serum vitamin D levels	Direct effect		4.918	3.8048 to 6.3557	4.56E-34	4.44%	
		Meditation effect		0.932	0.6163 to 1.4087	7.37E-01		
	Body mass index	Direct effect		4.919	4.0368 to 5.9945	3.40E-56	0.38%	
		Meditation effect		0.994	0.6800 to 1.4529	9.75E-01		
	Alcohol intake	Direct effect		4.902	3.8330 to 6.2693	9.21E-37	1.29%	
		Meditation effect		0.980	0.6522 to 1.4716	9.21E-01		
	Smoking	Direct effect		4.934	3.6965 to 6.5864	2.42E-27	0.61%	
		Meditation effect		0.990	0.6415 to 1.5286	9.65E-01		
Childhood sunburn	Total effect		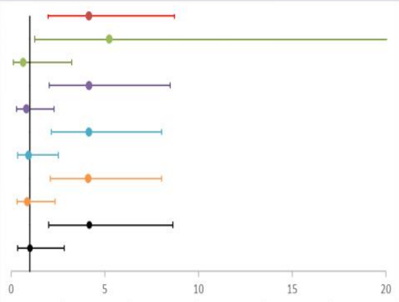	4.153	1.9774 to 8.7216	1.69E-04		Melanoma in situ (controls excluding all cancers)
	Skin colour	Direct effect		5.239	1.2542 to 21.8827	2.32E-02	30.82%	
		Meditation effect		0.645	0.1288 to 3.2280	5.93E-01		
	Serum vitamin D levels	Direct effect		4.159	2.0354 to 8.4991	9.26E-05	14.15%	
		Meditation effect		0.818	0.2918 to 2.2904	7.01E-01		
	Body mass index	Direct effect		4.154	2.1499 to 8.0252	2.26E-05	4.84%	
		Meditation effect		0.933	0.3461 to 2.5174	8.92E-01		
	Alcohol intake	Direct effect		4.115	2.1061 to 8.0406	3.48E-05	10.09%	
		Meditation effect		0.866	0.3188 to 2.3538	7.78E-01		
	Smoking	Direct effect		4.177	2.0218 to 8.6308	1.13E-04	0.68%	
		Meditation effect		1.010	0.3577 to 2.8509	9.85E-01		
Childhood sunburn	Total effect		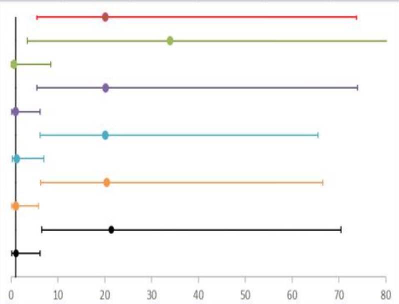	20.120	5.4920 to 73.7080	5.87E-06		Melanoma in situ of face (controls excluding all cancers)
	Skin colour	Direct effect		33.949	3.5048 to 328.8392	2.35E-03	15.74%	
		Meditation effect		0.623	0.0456 to 8.5264	7.23E-01		
	Serum vitamin D levels	Direct effect		20.185	5.5051 to 74.0131	5.81E-06	0.65%	
		Meditation effect		0.981	0.1563 to 6.1561	9.83E-01		
	Body mass index	Direct effect		20.124	6.1802 to 65.5262	6.23E-07	6.61%	
		Meditation effect		1.219	0.2109 to 7.0516	8.25E-01		
	Alcohol intake	Direct effect		20.429	6.2771 to 66.4875	5.41E-07	0.69%	
		Meditation effect		1.021	0.1766 to 5.9019	9.82E-01		
	Smoking	Direct effect		21.401	6.5078 to 70.3804	4.56E-07	2.18%	
		Meditation effect		1.068	0.1834 to 6.2146	9.42E-01		
Childhood sunburn	Total effect		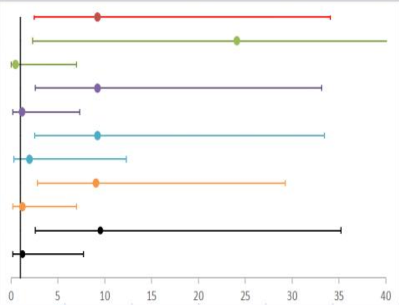	9.225	2.4969 to 34.0815	8.61E-04		Melanoma in situ of trunk (controls excluding all cancers)
	Skin colour	Direct effect		24.097	2.2991 to 252.5625	7.94E-03	33.52%	
		Meditation effect		0.475	0.0323 to 6.9851	5.87E-01		
	Serum vitamin D levels	Direct effect		9.227	2.5665 to 33.1698	6.65E-04	7.33%	
		Meditation effect		1.177	0.1890 to 7.3291	8.61E-01		
	Body mass index	Direct effect		9.228	2.5461 to 33.4423	7.18E-04	30.48%	
		Meditation effect		1.968	0.3143 to 12.3268	4.69E-01		
	Alcohol intake	Direct effect		9.063	2.8042 to 29.2889	2.31E-04	8.57%	
		Meditation effect		1.210	0.2089 to 7.0039	8.32E-01		
	Smoking	Direct effect		9.533	2.5820 to 35.2000	7.16E-04	9.05%	
		Meditation effect		1.223	0.1927 to 7.7584	8.31E-01		
Childhood sunburn	Total effect		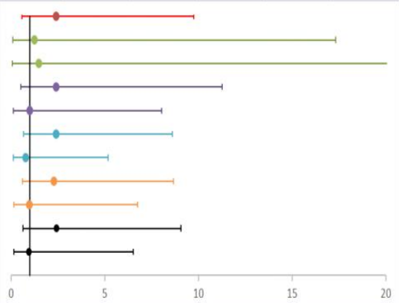	2.405	0.5925 to 9.7641	2.20E-01		Melanoma in situ of lowerlimb (controls excluding all cancers)
	Skin colour	Direct effect		1.248	0.0898 to 17.3285	8.69E-01	44.87%	
		Meditation effect		1.483	0.0752 to 29.2142	7.96E-01		
	Serum vitamin D levels	Direct effect		2.407	0.5142 to 11.2634	2.65E-01	0.18%	
		Meditation effect		0.998	0.1242 to 8.0270	9.99E-01		
	Body mass index	Direct effect		2.406	0.6724 to 8.6064	1.77E-01	28.44%	
		Meditation effect		0.779	0.1172 to 5.1783	7.96E-01		
	Alcohol intake	Direct effect		2.288	0.6041 to 8.6657	2.23E-01	2.53%	
		Meditation effect		0.978	0.1415 to 6.7579	9.82E-01		
	Smoking	Direct effect		2.425	0.6486 to 9.0692	1.88E-01	5.42%	
		Meditation effect		0.954	0.1392 to 6.5314	9.61E-01		
Childhood sunburn	Total effect		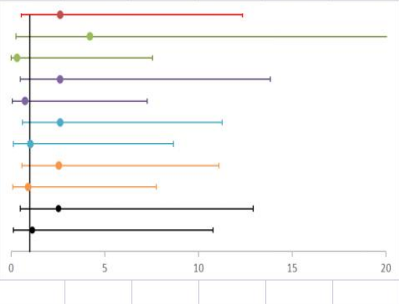	2.622	0.5565 to 12.3488	2.23E-01		Melanoma in situ of upperlimb (controls excluding all cancers)
	Skin colour	Direct effect		4.211	0.2674 to 66.2974	3.07E-01	NA	
		Meditation effect		0.319	0.0135 to 7.5476	4.79E-01		
	Serum vitamin D levels	Direct effect		2.617	0.4950 to 13.8334	2.58E-01	30.46%	
		Meditation effect		0.746	0.0767 to 7.2514	8.00E-01		
	Body mass index	Direct effect		2.622	0.6097 to 11.2711	1.95E-01	3.11%	
		Meditation effect		1.030	0.1227 to 8.6551	9.78E-01		
	Alcohol intake	Direct effect		2.549	0.5857 to 11.0947	2.12E-01	9.25%	
		Meditation effect		0.915	0.1080 to 7.7476	9.35E-01		
	Smoking	Direct effect		2.532	0.4963 to 12.9172	2.64E-01	13.34%	
		Meditation effect		1.137	0.1200 to 10.7772	9.11E-01		

Supplementary Figure 37. The forest plot of the replicate mediation MR analyses (controls excluding all cancers). Causal estimates given as odds ratio (OR) and 95% confidence intervals (CIs) for the effect of childhood sunburn and skin carcinoma risk. Red:total effect; Green: effect of skin colour; Purple: effect of serum vitamin D levels; Blue: effect of body mass index; Orange: effect of alcohol intake; Black: effect of smoking; Brown: effect of hair colour; Grey: effect of facial ageing.
