# Supplementary material for: Childhood sunburn and risk of melanoma and non-melanoma skin cancer: a Mendelian randomization study
Source: Environ Sci Pollut Res Int. 2023 Nov 14;30(58):122011–23. doi: 10.1007/s11356-023-30535-3 (PMC10724097; doi:10.1007/s11356-023-30535-3)
Supplement: Supplementary file 2 — (DOCX 217 kb) [file 11356_2023_30535_MOESM2_ESM.docx]

**Supplemenatary Table 1.** Summary of GWAS datasets included in this study

| **Phenotype** | **Released year** | **Sample size (case/control)** | **Number of SNPs** | **Population** | **Sex** | **Access** | **Category** |
| --- | --- | --- | --- | --- | --- | --- | --- |
| **Exposure** | | | | | | | |
| Childhood sunburn | 2018 | 456692 | 9851867 | European | Males and Females | UKB | Categorical Ordered |
| **Outcomes** | | | | | | | |
| Malignant melanoma | 2021 | 218792(98/218694) | 16380466 | European | Males and Females | FinnGen | Categorical Ordered |
| Malignant melanoma (all cancers excluded) | 2021 | 174104(98/174006) | 16380303 | European | Males and Females | FinnGen | Categorical Ordered |
| Melanoma in situ | 2021 | 309154(632/308522) | 16380466 | European | Males and Females | FinnGen | Categorical Ordered |
| Melanoma in situ (all cancers excluded) | 2021 | 249137(632/248505) | 16380337 | European | Males and Females | FinnGen | Categorical Ordered |
| Melanoma in situ of face | 2021 | 309154(178/308976) | 16380466 | European | Males and Females | FinnGen | Categorical Ordered |
| Melanoma in situ of face (all cancers excluded) | 2021 | 248823(178/248645) | 16380337 | European | Males and Females | FinnGen | Categorical Ordered |
| Melanoma in situ of trunk | 2021 | 309154(178/308976) | 16380466 | European | Males and Females | FinnGen | Categorical Ordered |
| Melanoma in situ of trunk (all cancers excluded) | 2021 | 248815(178/248637) | 16380337 | European | Males and Females | FinnGen | Categorical Ordered |
| Melanoma in situ of lower limb, including hip | 2021 | 309154(99/309055) | 16380466 | European | Males and Females | FinnGen | Categorical Ordered |
| Melanoma in situ of lower limb, including hip (all cancers excluded) | 2021 | 248763(99/248664) | 16380315 | European | Males and Females | FinnGen | Categorical Ordered |
| Melanoma in situ of upper limb, including shoulder | 2021 | 309154(89/309065) | 16380466 | European | Males and Females | FinnGen | Categorical Ordered |
| Melanoma in situ of upper limb, including shoulder (all cancers excluded) | 2021 | 248763(89/248674) | 16380267 | European | Males and Females | FinnGen | Categorical Ordered |
| Non-melanoma skin cancer | 2021 | 306688(13555/293133) | 16380466 | European | Males and Females | FinnGen | Categorical Ordered |
| Non-melanoma skin cancer (all cancers excluded) | 2021 | 252233(13555/238678) | 16380332 | European | Males and Females | FinnGen | Categorical Ordered |
| Squamous cell carcinomas | 2021 | 309154(2249/306905) | 16380332 | European | Males and Females | FinnGen | Categorical Ordered |
| Basal cell carcinoma | 2021 | 309154(14081/295073) | 16380209 | European | Males and Females | FinnGen | Categorical Ordered |
| **Mediators** | | | | | | | |
| Skin colour | 2018 | 456692 | 9851867 | European | Males and Females | UKB | Categorical Ordered |
| Hair colour | 2018 | 360270 | 13586531 | European | Males and Females | UKB | Categorical Ordered |
| Body mass index | 2018 | 461460 | 9851867 | European | Males and Females | UKB | Continues |
| Alcohol intake frequency. | 2018 | 462346 | 9851867 | European | Males and Females | UKB | Categorical Ordered |
| Serum 25-Hydroxyvitamin D levels | 2020 | 496946 | 6896093 | European | Males and Females | Liu M et.al. | Continues |
| Cigarettes smoked per day | 2019 | 249752 | 12003613 | European | Males and Females | Revez JA et.al. | Categorical Ordered |

**Supplementary Table 2.** Information of identified SNPs in exposure (Childhood sunburn) and outcomes (Malignant melanoma of skin and non-melanoma skin cancer).

| SNP | | | | | Exposure (Childhood sunburn) | | | | Outcome (Malignant melanoma of skin) | | | | Outcome (Non-melanoma skin cancer) | | | |
| --- | --- | --- | --- | --- | --- | --- | --- | --- | --- | --- | --- | --- | --- | --- | --- | --- |
| RS ID | Chr. | Position | EA | OA | Beta | Se | P-value | EAF | Beta | Se | P-value | EAF | Beta | Se | P-value | EAF |
| rs10168349 | 2 | 46360907 | C | G | 0.0173 | 0.0023 | 1.40E-14 | 0.3357 | -0.0015 | 0.1595 | 9.93E-01 | 0.2825 | 0.0239 | 0.0173 | 1.68E-01 | 0.2825 |
| rs10202908 | 2 | 169378292 | T | C | -0.0126 | 0.0023 | 2.90E-08 | 0.6749 | 0.2507 | 0.1561 | 1.08E-01 | 0.6989 | 0.0128 | 0.0170 | 4.49E-01 | 0.6989 |
| rs10220751 | 15 | 47923520 | G | T | -0.0137 | 0.0022 | 2.50E-10 | 0.4048 | 0.0395 | 0.1486 | 7.90E-01 | 0.3599 | -0.0174 | 0.0161 | 2.81E-01 | 0.3599 |
| rs10788627 | 10 | 82203069 | C | T | 0.0120 | 0.0021 | 1.60E-08 | 0.4745 | 0.0032 | 0.1436 | 9.82E-01 | 0.5463 | 0.0228 | 0.0156 | 1.44E-01 | 0.5463 |
| rs10810636 | 9 | 16799109 | G | A | 0.0275 | 0.0025 | 2.80E-28 | 0.7603 | 0.0360 | 0.1629 | 8.25E-01 | 0.7369 | 0.0749 | 0.0177 | 2.25E-05 | 0.7369 |
| rs10873552 | 14 | 105433129 | G | A | 0.0124 | 0.0022 | 3.70E-08 | 0.6577 | -0.0695 | 0.1450 | 6.31E-01 | 0.5732 | -0.0110 | 0.0157 | 4.84E-01 | 0.5732 |
| rs10896139 | 11 | 66650060 | T | C | -0.0136 | 0.0024 | 1.40E-08 | 0.2709 | -0.1855 | 0.1594 | 2.45E-01 | 0.2792 | 0.0115 | 0.0173 | 5.06E-01 | 0.2792 |
| rs11070811 | 15 | 31394082 | T | C | -0.0176 | 0.0027 | 1.50E-10 | 0.1832 | -0.1153 | 0.2097 | 5.82E-01 | 0.1331 | -0.0433 | 0.0228 | 5.79E-02 | 0.1331 |
| rs11104733 | 12 | 88490119 | T | C | 0.0492 | 0.0089 | 2.80E-08 | 0.0147 | -0.3401 | 0.4800 | 4.79E-01 | 0.0235 | 0.0834 | 0.0516 | 1.06E-01 | 0.0235 |
| rs111391498 | 4 | 1341553 | G | A | -0.0474 | 0.0050 | 1.70E-21 | 0.0479 | -0.3052 | 0.4286 | 4.76E-01 | 0.0288 | 0.0451 | 0.0461 | 3.27E-01 | 0.0288 |
| rs111650620 | 20 | 34591725 | A | G | 0.6618 | 0.0601 | 3.20E-28 | 0.0022 | -0.2503 | 0.4417 | 5.71E-01 | 0.0272 | 0.0205 | 0.0474 | 6.66E-01 | 0.0272 |
| rs112089506 | 16 | 90149171 | T | C | -0.0581 | 0.0040 | 1.40E-47 | 0.0812 | -0.1730 | 0.2488 | 4.87E-01 | 0.0934 | -0.1222 | 0.0271 | 6.39E-06 | 0.0934 |
| rs11242899 | 6 | 460302 | A | G | -0.0245 | 0.0024 | 3.20E-24 | 0.2663 | 0.0568 | 0.1631 | 7.28E-01 | 0.2626 | 0.0228 | 0.0177 | 1.98E-01 | 0.2626 |
| rs1126809 | 11 | 89017961 | A | G | 0.0825 | 0.0023 | 1.00E-200 | 0.3028 | -0.1031 | 0.1859 | 5.79E-01 | 0.1784 | 0.1630 | 0.0204 | 1.26E-15 | 0.1784 |
| rs116125333 | 5 | 33930012 | G | T | -0.0397 | 0.0072 | 3.40E-08 | 0.0232 | -0.0231 | 0.2626 | 9.30E-01 | 0.0842 | -0.0313 | 0.0282 | 2.67E-01 | 0.0842 |
| rs11648436 | 16 | 14008674 | T | C | -0.0273 | 0.0022 | 9.90E-35 | 0.3564 | -0.0725 | 0.1554 | 6.41E-01 | 0.3079 | -0.0051 | 0.0168 | 7.60E-01 | 0.3079 |
| rs117132860 | 7 | 17134708 | A | G | 0.0593 | 0.0067 | 1.20E-18 | 0.0256 | 1.8995 | 1.1521 | 9.92E-02 | 0.0040 | 0.4416 | 0.1259 | 4.52E-04 | 0.0040 |
| rs11739906 | 5 | 59019359 | C | A | 0.0130 | 0.0023 | 8.40E-09 | 0.3275 | 0.0000 | 0.1670 | 1.00E+00 | 0.2405 | 0.0047 | 0.0182 | 7.95E-01 | 0.2405 |
| rs117462393 | 16 | 14277804 | T | C | 0.0815 | 0.0096 | 1.50E-17 | 0.0144 | -0.0175 | 0.2931 | 9.52E-01 | 0.0648 | -0.0215 | 0.0320 | 5.01E-01 | 0.0648 |
| rs12203592 | 6 | 396321 | T | C | 0.1531 | 0.0025 | 1.00E-200 | 0.2191 | 0.1301 | 0.4194 | 7.56E-01 | 0.0307 | 0.3880 | 0.0473 | 2.45E-16 | 0.0307 |
| rs1233578 | 6 | 28712247 | G | A | -0.0206 | 0.0028 | 1.50E-13 | 0.1764 | -0.7463 | 0.3102 | 1.61E-02 | 0.0611 | 0.0446 | 0.0333 | 1.81E-01 | 0.0611 |
| rs12350739 | 9 | 16885017 | A | G | 0.0355 | 0.0022 | 5.80E-59 | 0.6068 | -0.1921 | 0.1434 | 1.81E-01 | 0.5454 | 0.0817 | 0.0156 | 1.55E-07 | 0.5454 |
| rs1260326 | 2 | 27730940 | C | T | -0.0125 | 0.0022 | 8.10E-09 | 0.6040 | 0.0807 | 0.1502 | 5.91E-01 | 0.6493 | 0.0093 | 0.0163 | 5.68E-01 | 0.6493 |
| rs1267038 | 2 | 162099153 | C | A | -0.0179 | 0.0030 | 2.20E-09 | 0.8449 | -0.0277 | 0.1858 | 8.81E-01 | 0.8185 | 0.0463 | 0.0202 | 2.21E-02 | 0.8185 |
| rs1278766 | 13 | 113534382 | C | T | 0.0191 | 0.0021 | 3.90E-19 | 0.5457 | -0.1389 | 0.1432 | 3.32E-01 | 0.4879 | 0.0520 | 0.0155 | 8.26E-04 | 0.4879 |
| rs12913832 | 15 | 28365618 | G | A | 0.0605 | 0.0025 | 1.90E-126 | 0.7753 | -0.0313 | 0.2354 | 8.94E-01 | 0.8942 | -0.1497 | 0.0255 | 4.56E-09 | 0.8942 |
| rs1308048 | 1 | 66888542 | C | T | -0.0204 | 0.0022 | 5.30E-21 | 0.4195 | 0.1634 | 0.1447 | 2.59E-01 | 0.4293 | -0.0566 | 0.0157 | 3.10E-04 | 0.4293 |
| rs1326798 | 9 | 12722227 | G | C | 0.0121 | 0.0022 | 3.60E-08 | 0.6206 | 0.5430 | 0.1500 | 2.95E-04 | 0.6355 | 0.0673 | 0.0161 | 2.97E-05 | 0.6355 |
| rs13332673 | 16 | 89940386 | T | G | -0.1021 | 0.0105 | 3.30E-22 | 0.0102 | -1.0547 | 0.8583 | 2.19E-01 | 0.0074 | -0.0126 | 0.0930 | 8.92E-01 | 0.0074 |
| rs139414522 | 16 | 89821155 | C | T | -0.0784 | 0.0091 | 5.40E-18 | 0.0153 | 0.6971 | 0.7699 | 3.65E-01 | 0.0093 | 0.1354 | 0.0821 | 9.88E-02 | 0.0093 |
| rs141817469 | 16 | 89927151 | T | C | -0.0932 | 0.0058 | 1.00E-57 | 0.0351 | 0.1419 | 0.4141 | 7.32E-01 | 0.0316 | -0.2023 | 0.0452 | 7.59E-06 | 0.0316 |
| rs142314514 | 16 | 90130996 | G | A | -0.0816 | 0.0070 | 3.60E-31 | 0.0285 | 0.0932 | 0.4497 | 8.36E-01 | 0.0280 | -0.1577 | 0.0498 | 1.55E-03 | 0.0280 |
| rs1437635 | 11 | 16358722 | A | C | 0.0207 | 0.0029 | 5.20E-13 | 0.1660 | -0.0880 | 0.2235 | 6.94E-01 | 0.1156 | 0.0284 | 0.0243 | 2.43E-01 | 0.1156 |
| rs150527451 | 11 | 68817897 | A | G | 0.0262 | 0.0035 | 4.00E-14 | 0.1064 | 0.0177 | 0.1709 | 9.18E-01 | 0.2348 | 0.0340 | 0.0184 | 6.53E-02 | 0.2348 |
| rs151337382 | 20 | 34204925 | A | T | 0.0575 | 0.0093 | 5.20E-10 | 0.0136 | 0.0470 | 1.0406 | 9.64E-01 | 0.0045 | -0.0318 | 0.1172 | 7.86E-01 | 0.0045 |
| rs1548714 | 17 | 26280204 | C | A | 0.0162 | 0.0027 | 3.00E-09 | 0.8117 | 0.1198 | 0.1837 | 5.14E-01 | 0.8124 | 0.0072 | 0.0199 | 7.19E-01 | 0.8124 |
| rs16891982 | 5 | 33951693 | G | C | 0.2292 | 0.0062 | 1.00E-200 | 0.9709 | 1.0331 | 0.5523 | 6.14E-02 | 0.9821 | 0.3583 | 0.0622 | 8.28E-09 | 0.9821 |
| rs17232484 | 16 | 89861650 | A | G | 0.0610 | 0.0091 | 1.80E-11 | 0.0142 | -0.2896 | 0.5558 | 6.02E-01 | 0.0199 | -0.0641 | 0.0598 | 2.84E-01 | 0.0199 |
| rs1805007 | 16 | 89986117 | T | C | 0.1964 | 0.0035 | 1.00E-200 | 0.1009 | 0.7378 | 0.2900 | 1.09E-02 | 0.0667 | 0.4515 | 0.0321 | 8.00E-45 | 0.0667 |
| rs1805008 | 16 | 89986144 | T | C | 0.1195 | 0.0038 | 1.00E-200 | 0.0866 | 0.1732 | 0.2793 | 5.35E-01 | 0.0687 | 0.3309 | 0.0310 | 1.39E-26 | 0.0687 |
| rs1989483 | 7 | 16942661 | G | A | 0.0125 | 0.0022 | 1.20E-08 | 0.3891 | 0.1936 | 0.1482 | 1.92E-01 | 0.3690 | 0.0228 | 0.0161 | 1.56E-01 | 0.3690 |
| rs2299098 | 7 | 24756377 | C | G | 0.0148 | 0.0027 | 3.60E-08 | 0.1962 | -0.0366 | 0.1965 | 8.52E-01 | 0.1559 | 0.0164 | 0.0214 | 4.45E-01 | 0.1559 |
| rs251468 | 5 | 149194485 | T | C | -0.0282 | 0.0025 | 4.40E-30 | 0.2484 | -0.0385 | 0.1511 | 7.99E-01 | 0.3441 | -0.0582 | 0.0164 | 3.72E-04 | 0.3441 |
| rs2737217 | 8 | 116630311 | G | A | -0.0255 | 0.0022 | 4.10E-32 | 0.5633 | 0.0292 | 0.1524 | 8.48E-01 | 0.6657 | -0.0412 | 0.0166 | 1.31E-02 | 0.6657 |
| rs3213737 | 12 | 96379806 | A | G | -0.0319 | 0.0022 | 2.70E-49 | 0.5760 | -0.2174 | 0.1500 | 1.47E-01 | 0.6553 | -0.0183 | 0.0163 | 2.61E-01 | 0.6553 |
| rs35563099 | 10 | 119572403 | T | C | -0.0310 | 0.0029 | 1.80E-26 | 0.1640 | -0.0442 | 0.1864 | 8.13E-01 | 0.1849 | -0.0637 | 0.0200 | 1.46E-03 | 0.1849 |
| rs3759579 | 14 | 103851272 | G | A | 0.0124 | 0.0022 | 8.10E-09 | 0.5888 | 0.0355 | 0.1476 | 8.10E-01 | 0.6206 | 0.0130 | 0.0160 | 4.19E-01 | 0.6206 |
| rs41563 | 7 | 104852654 | A | G | 0.0135 | 0.0022 | 1.40E-09 | 0.3498 | -0.2231 | 0.1552 | 1.51E-01 | 0.3078 | -0.0088 | 0.0168 | 5.99E-01 | 0.3078 |
| rs4240559 | 6 | 98437775 | C | T | -0.0171 | 0.0021 | 1.40E-15 | 0.5579 | -0.1825 | 0.1498 | 2.23E-01 | 0.6514 | -0.0061 | 0.0163 | 7.08E-01 | 0.6514 |
| rs4272574 | 1 | 73661205 | T | C | 0.0152 | 0.0021 | 8.40E-13 | 0.4783 | -0.0998 | 0.1432 | 4.86E-01 | 0.5101 | 0.0267 | 0.0156 | 8.61E-02 | 0.5101 |
| rs4335021 | 6 | 32386619 | C | T | -0.0118 | 0.0022 | 4.90E-08 | 0.5997 | -0.3636 | 0.1756 | 3.84E-02 | 0.6420 | -0.0284 | 0.0190 | 1.36E-01 | 0.6420 |
| rs4438032 | 11 | 88786692 | G | C | 0.0232 | 0.0036 | 2.00E-10 | 0.9062 | -0.4122 | 0.2748 | 1.34E-01 | 0.9271 | 0.0142 | 0.0300 | 6.36E-01 | 0.9271 |
| rs4578351 | 11 | 16587580 | C | T | -0.0205 | 0.0026 | 1.70E-15 | 0.2220 | 0.1111 | 0.1665 | 5.04E-01 | 0.2496 | -0.0193 | 0.0180 | 2.84E-01 | 0.2496 |
| rs4670813 | 2 | 38317710 | A | G | -0.0127 | 0.0021 | 3.50E-09 | 0.4708 | -0.3041 | 0.1438 | 3.45E-02 | 0.5243 | -0.0504 | 0.0156 | 1.21E-03 | 0.5243 |
| rs4840542 | 8 | 10944809 | T | G | 0.0185 | 0.0021 | 4.00E-18 | 0.5058 | -0.2031 | 0.2747 | 4.60E-01 | 0.4261 | 0.0404 | 0.0301 | 1.79E-01 | 0.4261 |
| rs511515 | 6 | 33541507 | G | A | -0.0204 | 0.0023 | 1.60E-18 | 0.7009 | -0.1301 | 0.1742 | 4.55E-01 | 0.7820 | -0.0168 | 0.0189 | 3.74E-01 | 0.7820 |
| rs537894 | 3 | 138348595 | A | G | 0.0127 | 0.0022 | 5.00E-09 | 0.5490 | 0.2103 | 0.1544 | 1.73E-01 | 0.6724 | 0.0218 | 0.0168 | 1.93E-01 | 0.6724 |
| rs57994353 | 9 | 139356987 | C | T | 0.0127 | 0.0023 | 4.30E-08 | 0.2991 | -0.2203 | 0.1638 | 1.79E-01 | 0.2556 | 0.0205 | 0.0178 | 2.50E-01 | 0.2556 |
| rs6007506 | 22 | 45622014 | T | C | -0.0248 | 0.0023 | 4.10E-28 | 0.3374 | 0.0301 | 0.1489 | 8.40E-01 | 0.3559 | -0.0245 | 0.0162 | 1.31E-01 | 0.3559 |
| rs6059655 | 20 | 32665748 | G | A | -0.1273 | 0.0036 | 1.00E-200 | 0.8980 | -0.2904 | 0.4700 | 5.37E-01 | 0.9773 | -0.3416 | 0.0523 | 6.52E-11 | 0.9773 |
| rs61816766 | 1 | 152319572 | C | T | 0.0366 | 0.0062 | 3.40E-09 | 0.0323 | -0.4250 | 0.7871 | 5.89E-01 | 0.0079 | 0.1192 | 0.0882 | 1.77E-01 | 0.0079 |
| rs61981034 | 14 | 97377089 | A | G | 0.0133 | 0.0024 | 4.60E-08 | 0.2580 | -0.0361 | 0.1518 | 8.12E-01 | 0.3367 | 0.0115 | 0.0164 | 4.84E-01 | 0.3367 |
| rs6689641 | 1 | 110720400 | G | A | 0.0149 | 0.0021 | 2.60E-12 | 0.5426 | 0.2368 | 0.1441 | 1.00E-01 | 0.5587 | 0.0288 | 0.0156 | 6.57E-02 | 0.5587 |
| rs6882046 | 5 | 87968864 | G | A | 0.0144 | 0.0024 | 3.10E-09 | 0.2679 | -0.0407 | 0.1855 | 8.26E-01 | 0.1804 | 0.0311 | 0.0203 | 1.24E-01 | 0.1804 |
| rs699780 | 1 | 120455441 | G | A | -0.0184 | 0.0032 | 7.00E-09 | 0.1278 | 0.0681 | 0.1908 | 7.21E-01 | 0.1682 | 0.0116 | 0.0208 | 5.75E-01 | 0.1682 |
| rs72821630 | 2 | 63696212 | T | C | -0.0150 | 0.0025 | 9.00E-10 | 0.2514 | -0.0444 | 0.1495 | 7.67E-01 | 0.3625 | 0.0125 | 0.0162 | 4.38E-01 | 0.3625 |
| rs75300484 | 16 | 89489061 | T | C | -0.0342 | 0.0058 | 3.70E-09 | 0.0347 | 0.0252 | 0.4616 | 9.56E-01 | 0.0233 | -0.1302 | 0.0516 | 1.17E-02 | 0.0233 |
| rs75908072 | 6 | 290438 | C | T | -0.0318 | 0.0057 | 2.80E-08 | 0.0366 | 0.6766 | 0.5078 | 1.83E-01 | 0.0227 | 0.0117 | 0.0552 | 8.33E-01 | 0.0227 |
| rs7768317 | 6 | 41922220 | T | C | -0.0170 | 0.0025 | 4.90E-12 | 0.2489 | 0.0020 | 0.1631 | 9.90E-01 | 0.2621 | -0.0262 | 0.0176 | 1.37E-01 | 0.2621 |
| rs784235 | 18 | 53423144 | G | A | -0.0164 | 0.0028 | 3.00E-09 | 0.8191 | -0.1430 | 0.2035 | 4.82E-01 | 0.8551 | -0.0167 | 0.0220 | 4.48E-01 | 0.8551 |
| rs78444298 | 1 | 184672098 | A | G | -0.0467 | 0.0077 | 1.40E-09 | 0.0197 | 0.8009 | 0.5534 | 1.48E-01 | 0.0172 | 0.0433 | 0.0599 | 4.71E-01 | 0.0172 |
| rs849138 | 7 | 28177338 | A | G | 0.0126 | 0.0021 | 3.80E-09 | 0.5070 | 0.0413 | 0.1432 | 7.73E-01 | 0.4910 | 0.0376 | 0.0155 | 1.54E-02 | 0.4910 |
| rs9328259 | 6 | 508972 | A | C | 0.0267 | 0.0024 | 1.60E-29 | 0.7182 | 0.1061 | 0.1595 | 5.06E-01 | 0.7206 | -0.0105 | 0.0173 | 5.44E-01 | 0.7206 |
| rs964127 | 12 | 96290733 | T | A | 0.0123 | 0.0023 | 4.90E-08 | 0.3328 | 0.0004 | 0.1434 | 9.98E-01 | 0.4643 | -0.0077 | 0.0156 | 6.22E-01 | 0.4643 |
| rs9821675 | 3 | 49902544 | G | A | -0.0120 | 0.0021 | 1.50E-08 | 0.5053 | -0.1457 | 0.1463 | 3.19E-01 | 0.3949 | -0.0398 | 0.0159 | 1.24E-02 | 0.3949 |
| rs9832130 | 3 | 189194752 | A | G | -0.0128 | 0.0022 | 3.30E-09 | 0.5807 | -0.0457 | 0.1446 | 7.52E-01 | 0.5615 | 0.0366 | 0.0157 | 1.98E-02 | 0.5615 |
| rs9835772 | 3 | 85766025 | T | A | 0.0177 | 0.0025 | 8.60E-13 | 0.2437 | 0.0277 | 0.1700 | 8.71E-01 | 0.2285 | 0.0048 | 0.0185 | 7.95E-01 | 0.2285 |
| rs9858244 | 3 | 85787399 | A | G | 0.0189 | 0.0026 | 3.00E-13 | 0.2142 | -0.0121 | 0.1839 | 9.47E-01 | 0.1843 | -0.0103 | 0.0200 | 6.06E-01 | 0.1843 |
| rs9867857 | 3 | 156491160 | T | C | 0.0136 | 0.0021 | 1.80E-10 | 0.4892 | 0.1962 | 0.1446 | 1.75E-01 | 0.4296 | 0.0373 | 0.0157 | 1.74E-02 | 0.4296 |
| rs9904468 | 17 | 17573187 | G | C | 0.0137 | 0.0021 | 1.10E-10 | 0.5335 | -0.2258 | 0.1434 | 1.15E-01 | 0.5500 | -0.0131 | 0.0156 | 4.00E-01 | 0.5500 |

SNP: Single-nucleotide polymorphisms; Chr: Chromosome; EA: Effect allele; OA: Other allele; EAF: Effect allele frequency.

**Supplementary Table 3.** Information of identified SNPs in exposure (Childhood sunburn) and outcomes (Skin squamous cell carcinomas and basal cell carcinomas).

| SNP | | | | | Exposure (Childhood sunburn) | | | | Outcome ( Squamous cell carcinomas of the skin) | | | | Outcome (Basal cell carcinomas of the skin) | | | |
| --- | --- | --- | --- | --- | --- | --- | --- | --- | --- | --- | --- | --- | --- | --- | --- | --- |
| RS ID | Chr. | Position | EA | OA | Beta | Se | P-value | EAF | Beta | Se | P-value | EAF | Beta | Se | P-value | EAF |
| rs10168349 | 2 | 46360907 | C | G | 0.0173 | 0.0023 | 1.40E-14 | 0.3357 | -0.0294 | 0.0338 | 3.85E-01 | 0.3125 | 0.0263 | 0.0141 | 6.23E-02 | 0.2821 |
| rs10202908 | 2 | 169378292 | T | C | -0.0126 | 0.0023 | 2.90E-08 | 0.6749 | -0.0073 | 0.0331 | 8.26E-01 | 0.6221 | -0.0076 | 0.0139 | 5.84E-01 | 0.6990 |
| rs10220751 | 15 | 47923520 | G | T | -0.0137 | 0.0022 | 2.50E-10 | 0.4048 | -0.0294 | 0.0315 | 3.50E-01 | 0.3785 | 0.0050 | 0.0132 | 7.02E-01 | 0.3600 |
| rs10788627 | 10 | 82203069 | C | T | 0.0120 | 0.0021 | 1.60E-08 | 0.4745 | 0.0756 | 0.0304 | 1.30E-02 | 0.4145 | 0.0118 | 0.0127 | 3.53E-01 | 0.5464 |
| rs10810636 | 9 | 16799109 | G | A | 0.0275 | 0.0025 | 2.80E-28 | 0.7603 | 0.0919 | 0.0348 | 8.28E-03 | 0.7677 | 0.0688 | 0.0145 | 2.14E-06 | 0.7367 |
| rs10873552 | 14 | 105433129 | G | A | 0.0124 | 0.0022 | 3.70E-08 | 0.6577 | 0.0553 | 0.0307 | 7.20E-02 | 0.5867 | -0.0085 | 0.0128 | 5.10E-01 | 0.5721 |
| rs10896139 | 11 | 66650060 | T | C | -0.0136 | 0.0024 | 1.40E-08 | 0.2709 | -0.0253 | 0.0338 | 4.54E-01 | 0.3101 | 0.0372 | 0.0141 | 8.16E-03 | 0.2797 |
| rs11070811 | 15 | 31394082 | T | C | -0.0176 | 0.0027 | 1.50E-10 | 0.1832 | 0.0383 | 0.0443 | 3.88E-01 | 0.1646 | -0.0861 | 0.0189 | 5.37E-06 | 0.1336 |
| rs11104733 | 12 | 88490119 | T | C | 0.0492 | 0.0089 | 2.80E-08 | 0.0147 | 0.0727 | 0.1011 | 4.72E-01 | 0.0472 | 0.1010 | 0.0420 | 1.62E-02 | 0.0235 |
| rs111391498 | 4 | 1341553 | G | A | -0.0474 | 0.0050 | 1.70E-21 | 0.0479 | -0.0628 | 0.0900 | 4.85E-01 | 0.0379 | 0.0508 | 0.0376 | 1.77E-01 | 0.0288 |
| rs111650620 | 20 | 34591725 | A | G | 0.6618 | 0.0601 | 3.20E-28 | 0.0022 | 0.1639 | 0.0921 | 7.52E-02 | 0.0034 | 0.0115 | 0.0386 | 7.66E-01 | 0.0272 |
| rs112089506 | 16 | 90149171 | T | C | -0.0581 | 0.0040 | 1.40E-47 | 0.0812 | -0.1469 | 0.0542 | 6.67E-03 | 0.0902 | -0.1129 | 0.0224 | 4.80E-07 | 0.0939 |
| rs11242899 | 6 | 460302 | A | G | -0.0245 | 0.0024 | 3.20E-24 | 0.2663 | -0.0337 | 0.0346 | 3.30E-01 | 0.3451 | 0.0106 | 0.0145 | 4.62E-01 | 0.2612 |
| rs1126809 | 11 | 89017961 | A | G | 0.0825 | 0.0023 | 1.00E-200 | 0.3028 | 0.1911 | 0.0378 | 4.35E-07 | 0.2341 | 0.1393 | 0.0162 | 7.45E-18 | 0.1790 |
| rs116125333 | 5 | 33930012 | G | T | -0.0397 | 0.0072 | 3.40E-08 | 0.0232 | -0.0795 | 0.0554 | 1.51E-01 | 0.0219 | -0.0133 | 0.0231 | 5.64E-01 | 0.0848 |
| rs11648436 | 16 | 14008674 | T | C | -0.0273 | 0.0022 | 9.90E-35 | 0.3564 | -0.0030 | 0.0329 | 9.27E-01 | 0.3564 | 0.0038 | 0.0137 | 7.80E-01 | 0.3070 |
| rs117132860 | 7 | 17134708 | A | G | 0.0593 | 0.0067 | 1.20E-18 | 0.0256 | 0.2033 | 0.2592 | 4.33E-01 | 0.0301 | 0.2076 | 0.1076 | 5.37E-02 | 0.0040 |
| rs11739906 | 5 | 59019359 | C | A | 0.0130 | 0.0023 | 8.40E-09 | 0.3275 | -0.0099 | 0.0355 | 7.81E-01 | 0.3153 | 0.0197 | 0.0148 | 1.83E-01 | 0.2405 |
| rs117462393 | 16 | 14277804 | T | C | 0.0815 | 0.0096 | 1.50E-17 | 0.0144 | -0.0001 | 0.0619 | 9.99E-01 | 0.0124 | -0.0288 | 0.0260 | 2.68E-01 | 0.0652 |
| rs12203592 | 6 | 396321 | T | C | 0.1531 | 0.0025 | 1.00E-200 | 0.2191 | 0.5437 | 0.0755 | 5.89E-13 | 0.1983 | 0.3837 | 0.0340 | 1.65E-29 | 0.0312 |
| rs1233578 | 6 | 28712247 | G | A | -0.0206 | 0.0028 | 1.50E-13 | 0.1764 | 0.0511 | 0.0622 | 4.12E-01 | 0.1891 | 0.0036 | 0.0263 | 8.91E-01 | 0.0615 |
| rs12350739 | 9 | 16885017 | A | G | 0.0355 | 0.0022 | 5.80E-59 | 0.6068 | 0.1153 | 0.0303 | 1.43E-04 | 0.5628 | 0.1034 | 0.0127 | 3.90E-16 | 0.5451 |
| rs1260326 | 2 | 27730940 | C | T | -0.0125 | 0.0022 | 8.10E-09 | 0.6040 | -0.0129 | 0.0319 | 6.86E-01 | 0.5983 | -0.0146 | 0.0133 | 2.73E-01 | 0.6502 |
| rs1267038 | 2 | 162099153 | C | A | -0.0179 | 0.0030 | 2.20E-09 | 0.8449 | 0.0127 | 0.0395 | 7.47E-01 | 0.9747 | 0.0037 | 0.0165 | 8.25E-01 | 0.8189 |
| rs1278766 | 13 | 113534382 | C | T | 0.0191 | 0.0021 | 3.90E-19 | 0.5457 | -0.0028 | 0.0303 | 9.27E-01 | 0.5682 | 0.0502 | 0.0126 | 7.33E-05 | 0.4868 |
| rs12913832 | 15 | 28365618 | G | A | 0.0605 | 0.0025 | 1.90E-126 | 0.7753 | -0.2116 | 0.0480 | 1.05E-05 | 0.8027 | -0.1457 | 0.0206 | 1.51E-12 | 0.8957 |
| rs1308048 | 1 | 66888542 | C | T | -0.0204 | 0.0022 | 5.30E-21 | 0.4195 | -0.0480 | 0.0307 | 1.17E-01 | 0.3925 | -0.0403 | 0.0128 | 1.66E-03 | 0.4294 |
| rs1326798 | 9 | 12722227 | G | C | 0.0121 | 0.0022 | 3.60E-08 | 0.6206 | 0.0787 | 0.0315 | 1.25E-02 | 0.7677 | 0.0546 | 0.0132 | 3.59E-05 | 0.6360 |
| rs13332673 | 16 | 89940386 | T | G | -0.1021 | 0.0105 | 3.30E-22 | 0.0102 | -0.2551 | 0.1973 | 1.96E-01 | 0.0216 | -0.1126 | 0.0825 | 1.73E-01 | 0.0067 |
| rs139414522 | 16 | 89821155 | C | T | -0.0784 | 0.0091 | 5.40E-18 | 0.0153 | -0.0878 | 0.1590 | 5.81E-01 | 0.0180 | 0.0494 | 0.0666 | 4.58E-01 | 0.0092 |
| rs141817469 | 16 | 89927151 | T | C | -0.0932 | 0.0058 | 1.00E-57 | 0.0351 | -0.1078 | 0.0883 | 2.22E-01 | 0.0318 | -0.1378 | 0.0380 | 2.90E-04 | 0.0319 |
| rs142314514 | 16 | 90130996 | G | A | -0.0816 | 0.0070 | 3.60E-31 | 0.0285 | -0.1155 | 0.0966 | 2.32E-01 | 0.0292 | -0.1214 | 0.0417 | 3.62E-03 | 0.0278 |
| rs1437635 | 11 | 16358722 | A | C | 0.0207 | 0.0029 | 5.20E-13 | 0.1660 | -0.0841 | 0.0475 | 7.63E-02 | 0.1522 | 0.0055 | 0.0198 | 7.80E-01 | 0.1156 |
| rs150527451 | 11 | 68817897 | A | G | 0.0262 | 0.0035 | 4.00E-14 | 0.1064 | 0.0832 | 0.0361 | 2.10E-02 | 0.2345 | 0.0489 | 0.0149 | 1.07E-03 | 0.2345 |
| rs151337382 | 20 | 34204925 | A | T | 0.0575 | 0.0093 | 5.20E-10 | 0.0136 | 0.2705 | 0.2311 | 2.42E-01 | 0.0075 | 0.1185 | 0.0972 | 2.22E-01 | 0.0045 |
| rs1548714 | 17 | 26280204 | C | A | 0.0162 | 0.0027 | 3.00E-09 | 0.8117 | 0.0074 | 0.0389 | 8.49E-01 | 0.8035 | 0.0129 | 0.0162 | 4.28E-01 | 0.8125 |
| rs16891982 | 5 | 33951693 | G | C | 0.2292 | 0.0062 | 1.00E-200 | 0.9709 | 0.4141 | 0.1427 | 3.70E-03 | 0.9812 | 0.2670 | 0.0566 | 2.39E-06 | 0.9834 |
| rs17232484 | 16 | 89861650 | A | G | 0.0610 | 0.0091 | 1.80E-11 | 0.0142 | -0.0923 | 0.1167 | 4.29E-01 | 0.0223 | -0.0384 | 0.0486 | 4.29E-01 | 0.0201 |
| rs1805007 | 16 | 89986117 | T | C | 0.1964 | 0.0035 | 1.00E-200 | 0.1009 | 0.5129 | 0.0524 | 1.16E-22 | 0.0766 | 0.3514 | 0.0234 | 7.06E-51 | 0.0663 |
| rs1805008 | 16 | 89986144 | T | C | 0.1195 | 0.0038 | 1.00E-200 | 0.0866 | 0.3023 | 0.0547 | 3.33E-08 | 0.0707 | 0.2527 | 0.0235 | 6.50E-27 | 0.0688 |
| rs1989483 | 7 | 16942661 | G | A | 0.0125 | 0.0022 | 1.20E-08 | 0.3891 | 0.0271 | 0.0315 | 3.88E-01 | 0.3698 | 0.0418 | 0.0131 | 1.39E-03 | 0.3698 |
| rs2299098 | 7 | 24756377 | C | G | 0.0148 | 0.0027 | 3.60E-08 | 0.1962 | 0.0517 | 0.0417 | 2.15E-01 | 0.1676 | -0.0205 | 0.0174 | 2.40E-01 | 0.1555 |
| rs251468 | 5 | 149194485 | T | C | -0.0282 | 0.0025 | 4.40E-30 | 0.2484 | -0.0709 | 0.0319 | 2.64E-02 | 0.3234 | -0.0533 | 0.0134 | 6.94E-05 | 0.3435 |
| rs2737217 | 8 | 116630311 | G | A | -0.0255 | 0.0022 | 4.10E-32 | 0.5633 | -0.0273 | 0.0323 | 3.98E-01 | 0.6928 | -0.0490 | 0.0134 | 2.64E-04 | 0.6646 |
| rs3213737 | 12 | 96379806 | A | G | -0.0319 | 0.0022 | 2.70E-49 | 0.5760 | -0.0157 | 0.0319 | 6.22E-01 | 0.5682 | -0.0114 | 0.0133 | 3.90E-01 | 0.6552 |
| rs35563099 | 10 | 119572403 | T | C | -0.0310 | 0.0029 | 1.80E-26 | 0.1640 | -0.1563 | 0.0402 | 1.02E-04 | 0.2019 | -0.0569 | 0.0165 | 5.62E-04 | 0.1837 |
| rs3759579 | 14 | 103851272 | G | A | 0.0124 | 0.0022 | 8.10E-09 | 0.5888 | 0.0401 | 0.0312 | 1.99E-01 | 0.6021 | 0.0294 | 0.0131 | 2.45E-02 | 0.6198 |
| rs41563 | 7 | 104852654 | A | G | 0.0135 | 0.0022 | 1.40E-09 | 0.3498 | -0.0564 | 0.0328 | 8.59E-02 | 0.3208 | 0.0045 | 0.0137 | 7.45E-01 | 0.3071 |
| rs4240559 | 6 | 98437775 | C | T | -0.0171 | 0.0021 | 1.40E-15 | 0.5579 | -0.0024 | 0.0318 | 9.40E-01 | 0.5965 | 0.0014 | 0.0133 | 9.18E-01 | 0.6521 |
| rs4272574 | 1 | 73661205 | T | C | 0.0152 | 0.0021 | 8.40E-13 | 0.4783 | 0.0444 | 0.0304 | 1.43E-01 | 0.5122 | 0.0302 | 0.0127 | 1.74E-02 | 0.5098 |
| rs4335021 | 6 | 32386619 | C | T | -0.0118 | 0.0022 | 4.90E-08 | 0.5997 | -0.0809 | 0.0311 | 9.33E-03 | 0.6473 | -0.0539 | 0.0131 | 3.89E-05 | 0.6412 |
| rs4438032 | 11 | 88786692 | G | C | 0.0232 | 0.0036 | 2.00E-10 | 0.9062 | -0.0602 | 0.0586 | 3.04E-01 | 0.9271 | 0.0143 | 0.0245 | 5.59E-01 | 0.9272 |
| rs4578351 | 11 | 16587580 | C | T | -0.0205 | 0.0026 | 1.70E-15 | 0.2220 | -0.0508 | 0.0354 | 1.51E-01 | 0.2650 | -0.0215 | 0.0148 | 1.46E-01 | 0.2474 |
| rs4670813 | 2 | 38317710 | A | G | -0.0127 | 0.0021 | 3.50E-09 | 0.4708 | -0.0233 | 0.0305 | 4.45E-01 | 0.5274 | -0.0471 | 0.0127 | 2.13E-04 | 0.5242 |
| rs4840542 | 8 | 10944809 | T | G | 0.0185 | 0.0021 | 4.00E-18 | 0.5058 | -0.0364 | 0.0308 | 2.38E-01 | 0.4133 | -0.0020 | 0.0129 | 8.76E-01 | 0.4270 |
| rs511515 | 6 | 33541507 | G | A | -0.0204 | 0.0023 | 1.60E-18 | 0.7009 | 0.0108 | 0.0366 | 7.69E-01 | 0.7982 | 0.0049 | 0.0153 | 7.50E-01 | 0.7814 |
| rs537894 | 3 | 138348595 | A | G | 0.0127 | 0.0022 | 5.00E-09 | 0.5490 | -0.0217 | 0.0327 | 5.07E-01 | 0.6724 | 0.0248 | 0.0137 | 6.96E-02 | 0.6732 |
| rs57994353 | 9 | 139356987 | C | T | 0.0127 | 0.0023 | 4.30E-08 | 0.2991 | 0.0841 | 0.0347 | 1.54E-02 | 0.2556 | 0.0462 | 0.0144 | 1.36E-03 | 0.2551 |
| rs6007506 | 22 | 45622014 | T | C | -0.0248 | 0.0023 | 4.10E-28 | 0.3374 | -0.0474 | 0.0316 | 1.34E-01 | 0.3560 | -0.0189 | 0.0132 | 1.52E-01 | 0.3559 |
| rs6059655 | 20 | 32665748 | G | A | -0.1273 | 0.0036 | 1.00E-200 | 0.8980 | -0.3272 | 0.0918 | 3.65E-04 | 0.9778 | -0.3126 | 0.0395 | 2.38E-15 | 0.9775 |
| rs61816766 | 1 | 152319572 | C | T | 0.0366 | 0.0062 | 3.40E-09 | 0.0323 | 0.2092 | 0.1716 | 2.23E-01 | 0.0079 | 0.0950 | 0.0718 | 1.86E-01 | 0.0079 |
| rs61981034 | 14 | 97377089 | A | G | 0.0133 | 0.0024 | 4.60E-08 | 0.2580 | 0.0114 | 0.0320 | 7.22E-01 | 0.3724 | 0.0054 | 0.0134 | 6.86E-01 | 0.3376 |
| rs6689641 | 1 | 110720400 | G | A | 0.0149 | 0.0021 | 2.60E-12 | 0.5426 | 0.0387 | 0.0305 | 2.05E-01 | 0.5716 | 0.0289 | 0.0128 | 2.36E-02 | 0.5590 |
| rs6882046 | 5 | 87968864 | G | A | 0.0144 | 0.0024 | 3.10E-09 | 0.2679 | -0.0773 | 0.0395 | 5.07E-02 | 0.1750 | 0.0295 | 0.0166 | 7.51E-02 | 0.1807 |
| rs699780 | 1 | 120455441 | G | A | -0.0184 | 0.0032 | 7.00E-09 | 0.1278 | -0.0474 | 0.0406 | 2.42E-01 | 0.1688 | 0.0148 | 0.0170 | 3.84E-01 | 0.1684 |
| rs72821630 | 2 | 63696212 | T | C | -0.0150 | 0.0025 | 9.00E-10 | 0.2514 | 0.0057 | 0.0316 | 8.56E-01 | 0.2346 | 0.0033 | 0.0132 | 8.05E-01 | 0.3624 |
| rs75300484 | 16 | 89489061 | T | C | -0.0342 | 0.0058 | 3.70E-09 | 0.0347 | -0.1717 | 0.0996 | 8.47E-02 | 0.0296 | -0.0893 | 0.0419 | 3.31E-02 | 0.0234 |
| rs75908072 | 6 | 290438 | C | T | -0.0318 | 0.0057 | 2.80E-08 | 0.0366 | 0.1342 | 0.1075 | 2.12E-01 | 0.0327 | 0.0149 | 0.0454 | 7.42E-01 | 0.0232 |
| rs7768317 | 6 | 41922220 | T | C | -0.0170 | 0.0025 | 4.90E-12 | 0.2489 | -0.0395 | 0.0345 | 2.53E-01 | 0.2763 | -0.0453 | 0.0145 | 1.75E-03 | 0.2617 |
| rs784235 | 18 | 53423144 | G | A | -0.0164 | 0.0028 | 3.00E-09 | 0.8191 | -0.0288 | 0.0431 | 5.04E-01 | 0.8216 | -0.0244 | 0.0180 | 1.75E-01 | 0.8556 |
| rs78444298 | 1 | 184672098 | A | G | -0.0467 | 0.0077 | 1.40E-09 | 0.0197 | 0.1027 | 0.1154 | 3.74E-01 | 0.0187 | 0.0311 | 0.0487 | 5.22E-01 | 0.0174 |
| rs849138 | 7 | 28177338 | A | G | 0.0126 | 0.0021 | 3.80E-09 | 0.5070 | 0.0352 | 0.0303 | 2.45E-01 | 0.5419 | 0.0491 | 0.0126 | 1.04E-04 | 0.4910 |
| rs9328259 | 6 | 508972 | A | C | 0.0267 | 0.0024 | 1.60E-29 | 0.7182 | 0.0224 | 0.0338 | 5.07E-01 | 0.7312 | 0.0032 | 0.0141 | 8.22E-01 | 0.7206 |
| rs964127 | 12 | 96290733 | T | A | 0.0123 | 0.0023 | 4.90E-08 | 0.3328 | -0.0075 | 0.0304 | 8.05E-01 | 0.4265 | -0.0161 | 0.0127 | 2.04E-01 | 0.4648 |
| rs9821675 | 3 | 49902544 | G | A | -0.0120 | 0.0021 | 1.50E-08 | 0.5053 | -0.0869 | 0.0311 | 5.26E-03 | 0.3934 | -0.0473 | 0.0130 | 2.76E-04 | 0.3949 |
| rs9832130 | 3 | 189194752 | A | G | -0.0128 | 0.0022 | 3.30E-09 | 0.5807 | -0.1195 | 0.0304 | 8.58E-05 | 0.5612 | 0.0191 | 0.0128 | 1.37E-01 | 0.5637 |
| rs9835772 | 3 | 85766025 | T | A | 0.0177 | 0.0025 | 8.60E-13 | 0.2437 | -0.0135 | 0.0361 | 7.08E-01 | 0.3123 | 0.0032 | 0.0151 | 8.32E-01 | 0.2289 |
| rs9858244 | 3 | 85787399 | A | G | 0.0189 | 0.0026 | 3.00E-13 | 0.2142 | -0.0069 | 0.0391 | 8.60E-01 | 0.1815 | -0.0085 | 0.0163 | 6.03E-01 | 0.1847 |
| rs9867857 | 3 | 156491160 | T | C | 0.0136 | 0.0021 | 1.80E-10 | 0.4892 | 0.0498 | 0.0307 | 1.04E-01 | 0.4729 | 0.0342 | 0.0128 | 7.40E-03 | 0.4290 |
| rs9904468 | 17 | 17573187 | G | C | 0.0137 | 0.0021 | 1.10E-10 | 0.5335 | 0.1069 | 0.0302 | 3.91E-04 | 0.4955 | 0.0034 | 0.0127 | 7.91E-01 | 0.5497 |

SNP: Single-nucleotide polymorphisms; Chr: Chromosome; EA: Effect allele; OA: Other allele; EAF: Effect allele frequency.

**Supplementary Table 4.** Information of identified SNPs in exposure (Childhood sunburn) and outcomes (overall Melanoma in situ and Melanoma in situ of face).

| SNP | | | | | Exposure (Childhood sunburn) | | | | Outcome (Melanoma in situ) | | | | Outcome (Melanoma in situ of face) | | | |
| --- | --- | --- | --- | --- | --- | --- | --- | --- | --- | --- | --- | --- | --- | --- | --- | --- |
| RS ID | Chr. | Position | EA | OA | Beta | Se | P-  value | EAF | Beta | Se | P-  value | EAF | Beta | Se | P-  value | EAF |
| rs10168349 | 2 | 46360907 | C | G | 0.0173 | 0.0023 | 1.40E-14 | 0.3357 | 0.1443 | 0.0807 | 7.37E-02 | 0.2825 | 0.1954 | 0.1463 | 1.82E-01 | 0.2825 |
| rs10202908 | 2 | 169378292 | T | C | -0.0126 | 0.0023 | 2.90E-08 | 0.6749 | -0.0231 | 0.0788 | 7.69E-01 | 0.6989 | 0.0790 | 0.1426 | 5.80E-01 | 0.6989 |
| rs10220751 | 15 | 47923520 | G | T | -0.0137 | 0.0022 | 2.50E-10 | 0.4048 | -0.0207 | 0.0751 | 7.83E-01 | 0.3599 | 0.0093 | 0.1361 | 9.45E-01 | 0.3599 |
| rs10788627 | 10 | 82203069 | C | T | 0.0120 | 0.0021 | 1.60E-08 | 0.4745 | -0.0619 | 0.0726 | 3.94E-01 | 0.5463 | -0.0724 | 0.1320 | 5.83E-01 | 0.5463 |
| rs10810636 | 9 | 16799109 | G | A | 0.0275 | 0.0025 | 2.80E-28 | 0.7603 | 0.0410 | 0.0823 | 6.18E-01 | 0.7369 | 0.1349 | 0.1488 | 3.65E-01 | 0.7369 |
| rs10873552 | 14 | 105433129 | G | A | 0.0124 | 0.0022 | 3.70E-08 | 0.6577 | 0.0209 | 0.0733 | 7.76E-01 | 0.5732 | -0.0694 | 0.1326 | 6.01E-01 | 0.5732 |
| rs10896139 | 11 | 66650060 | T | C | -0.0136 | 0.0024 | 1.40E-08 | 0.2709 | -0.0044 | 0.0809 | 9.57E-01 | 0.2792 | 0.0074 | 0.1463 | 9.59E-01 | 0.2792 |
| rs11070811 | 15 | 31394082 | T | C | -0.0176 | 0.0027 | 1.50E-10 | 0.1832 | -0.1399 | 0.1058 | 1.86E-01 | 0.1331 | -0.2444 | 0.1919 | 2.03E-01 | 0.1331 |
| rs11104733 | 12 | 88490119 | T | C | 0.0492 | 0.0089 | 2.80E-08 | 0.0147 | 0.6426 | 0.2433 | 8.25E-03 | 0.0235 | 1.0599 | 0.4383 | 1.56E-02 | 0.0235 |
| rs111391498 | 4 | 1341553 | G | A | -0.0474 | 0.0050 | 1.70E-21 | 0.0479 | -0.0432 | 0.2162 | 8.42E-01 | 0.0288 | 0.3361 | 0.3932 | 3.93E-01 | 0.0288 |
| rs111650620 | 20 | 34591725 | A | G | 0.6618 | 0.0601 | 3.20E-28 | 0.0022 | -0.0157 | 0.2229 | 9.44E-01 | 0.0272 | 0.0864 | 0.4013 | 8.30E-01 | 0.0272 |
| rs112089506 | 16 | 90149171 | T | C | -0.0581 | 0.0040 | 1.40E-47 | 0.0812 | -0.1702 | 0.1250 | 1.73E-01 | 0.0934 | -0.0481 | 0.2273 | 8.33E-01 | 0.0934 |
| rs11242899 | 6 | 460302 | A | G | -0.0245 | 0.0024 | 3.20E-24 | 0.2663 | 0.0329 | 0.0824 | 6.90E-01 | 0.2626 | -0.0245 | 0.1496 | 8.70E-01 | 0.2626 |
| rs1126809 | 11 | 89017961 | A | G | 0.0825 | 0.0023 | 1.00E-200 | 0.3028 | 0.2609 | 0.0939 | 5.49E-03 | 0.1784 | 0.4211 | 0.1705 | 1.35E-02 | 0.1784 |
| rs116125333 | 5 | 33930012 | G | T | -0.0397 | 0.0072 | 3.40E-08 | 0.0232 | -0.1021 | 0.1326 | 4.41E-01 | 0.0842 | -0.1796 | 0.2417 | 4.58E-01 | 0.0842 |
| rs11648436 | 16 | 14008674 | T | C | -0.0273 | 0.0022 | 9.90E-35 | 0.3564 | 0.0182 | 0.0783 | 8.16E-01 | 0.3079 | -0.0066 | 0.1420 | 9.63E-01 | 0.3079 |
| rs117132860 | 7 | 17134708 | A | G | 0.0593 | 0.0067 | 1.20E-18 | 0.0256 | 0.8889 | 0.5701 | 1.19E-01 | 0.0040 | 2.3649 | 1.1058 | 3.25E-02 | 0.0040 |
| rs11739906 | 5 | 59019359 | C | A | 0.0130 | 0.0023 | 8.40E-09 | 0.3275 | -0.0410 | 0.0843 | 6.27E-01 | 0.2405 | -0.2085 | 0.1533 | 1.74E-01 | 0.2405 |
| rs117462393 | 16 | 14277804 | T | C | 0.0815 | 0.0096 | 1.50E-17 | 0.0144 | 0.0899 | 0.1491 | 5.46E-01 | 0.0648 | 0.5485 | 0.2683 | 4.09E-02 | 0.0648 |
| rs12203592 | 6 | 396321 | T | C | 0.1531 | 0.0025 | 1.00E-200 | 0.2191 | 0.2227 | 0.2100 | 2.89E-01 | 0.0307 | 0.4124 | 0.3827 | 2.81E-01 | 0.0307 |
| rs1233578 | 6 | 28712247 | G | A | -0.0206 | 0.0028 | 1.50E-13 | 0.1764 | 0.0515 | 0.1480 | 7.28E-01 | 0.0611 | 0.4157 | 0.2733 | 1.28E-01 | 0.0611 |
| rs12350739 | 9 | 16885017 | A | G | 0.0355 | 0.0022 | 5.80E-59 | 0.6068 | 0.0152 | 0.0724 | 8.34E-01 | 0.5454 | 0.0480 | 0.1314 | 7.15E-01 | 0.5454 |
| rs1260326 | 2 | 27730940 | C | T | -0.0125 | 0.0022 | 8.10E-09 | 0.6040 | -0.0887 | 0.0761 | 2.44E-01 | 0.6493 | -0.1712 | 0.1381 | 2.15E-01 | 0.6493 |
| rs1267038 | 2 | 162099153 | C | A | -0.0179 | 0.0030 | 2.20E-09 | 0.8449 | 0.0698 | 0.0944 | 4.60E-01 | 0.8185 | -0.1780 | 0.1709 | 2.98E-01 | 0.8185 |
| rs1278766 | 13 | 113534382 | C | T | 0.0191 | 0.0021 | 3.90E-19 | 0.5457 | 0.0285 | 0.0724 | 6.93E-01 | 0.4879 | 0.0683 | 0.1314 | 6.03E-01 | 0.4879 |
| rs12913832 | 15 | 28365618 | G | A | 0.0605 | 0.0025 | 1.90E-126 | 0.7753 | -0.2981 | 0.1198 | 1.29E-02 | 0.8942 | 0.1111 | 0.2167 | 6.08E-01 | 0.8942 |
| rs1308048 | 1 | 66888542 | C | T | -0.0204 | 0.0022 | 5.30E-21 | 0.4195 | -0.0630 | 0.0731 | 3.89E-01 | 0.4293 | 0.2623 | 0.1324 | 4.77E-02 | 0.4293 |
| rs1326798 | 9 | 12722227 | G | C | 0.0121 | 0.0022 | 3.60E-08 | 0.6206 | 0.0635 | 0.0751 | 3.97E-01 | 0.6355 | 0.0244 | 0.1360 | 8.58E-01 | 0.6355 |
| rs13332673 | 16 | 89940386 | T | G | -0.1021 | 0.0105 | 3.30E-22 | 0.0102 | -0.4862 | 0.4227 | 2.50E-01 | 0.0074 | -1.0594 | 0.7761 | 1.72E-01 | 0.0074 |
| rs139414522 | 16 | 89821155 | C | T | -0.0784 | 0.0091 | 5.40E-18 | 0.0153 | -0.1616 | 0.3820 | 6.72E-01 | 0.0093 | -1.0359 | 0.7058 | 1.42E-01 | 0.0093 |
| rs141817469 | 16 | 89927151 | T | C | -0.0932 | 0.0058 | 1.00E-57 | 0.0351 | -0.0299 | 0.2116 | 8.88E-01 | 0.0316 | -0.1673 | 0.3860 | 6.65E-01 | 0.0316 |
| rs142314514 | 16 | 90130996 | G | A | -0.0816 | 0.0070 | 3.60E-31 | 0.0285 | -0.0731 | 0.2260 | 7.46E-01 | 0.0280 | 0.3858 | 0.4088 | 3.45E-01 | 0.0280 |
| rs1437635 | 11 | 16358722 | A | C | 0.0207 | 0.0029 | 5.20E-13 | 0.1660 | -0.1876 | 0.1140 | 9.99E-02 | 0.1156 | 0.1357 | 0.2071 | 5.12E-01 | 0.1156 |
| rs150527451 | 11 | 68817897 | A | G | 0.0262 | 0.0035 | 4.00E-14 | 0.1064 | -0.1194 | 0.0863 | 1.67E-01 | 0.2348 | -0.2151 | 0.1566 | 1.70E-01 | 0.2348 |
| rs151337382 | 20 | 34204925 | A | T | 0.0575 | 0.0093 | 5.20E-10 | 0.0136 | -0.5662 | 0.5465 | 3.00E-01 | 0.0045 | -0.0387 | 1.0048 | 9.69E-01 | 0.0045 |
| rs1548714 | 17 | 26280204 | C | A | 0.0162 | 0.0027 | 3.00E-09 | 0.8117 | -0.1350 | 0.0925 | 1.45E-01 | 0.8124 | -0.0190 | 0.1681 | 9.10E-01 | 0.8124 |
| rs16891982 | 5 | 33951693 | G | C | 0.2292 | 0.0062 | 1.00E-200 | 0.9709 | 0.2834 | 0.2795 | 3.11E-01 | 0.9821 | 0.2896 | 0.5085 | 5.69E-01 | 0.9821 |
| rs17232484 | 16 | 89861650 | A | G | 0.0610 | 0.0091 | 1.80E-11 | 0.0142 | 0.1656 | 0.2790 | 5.53E-01 | 0.0199 | 0.6794 | 0.5077 | 1.81E-01 | 0.0199 |
| rs1805007 | 16 | 89986117 | T | C | 0.1964 | 0.0035 | 1.00E-200 | 0.1009 | 0.3186 | 0.1452 | 2.83E-02 | 0.0667 | 0.2176 | 0.2644 | 4.11E-01 | 0.0667 |
| rs1805008 | 16 | 89986144 | T | C | 0.1195 | 0.0038 | 1.00E-200 | 0.0866 | 0.3323 | 0.1396 | 1.73E-02 | 0.0687 | 0.6855 | 0.2546 | 7.10E-03 | 0.0687 |
| rs1989483 | 7 | 16942661 | G | A | 0.0125 | 0.0022 | 1.20E-08 | 0.3891 | 0.0967 | 0.0747 | 1.96E-01 | 0.3690 | 0.0993 | 0.1356 | 4.64E-01 | 0.3690 |
| rs2299098 | 7 | 24756377 | C | G | 0.0148 | 0.0027 | 3.60E-08 | 0.1962 | 0.1352 | 0.0992 | 1.73E-01 | 0.1559 | 0.3680 | 0.1792 | 4.00E-02 | 0.1559 |
| rs251468 | 5 | 149194485 | T | C | -0.0282 | 0.0025 | 4.40E-30 | 0.2484 | 0.0132 | 0.0762 | 8.62E-01 | 0.3441 | -0.2138 | 0.1381 | 1.22E-01 | 0.3441 |
| rs2737217 | 8 | 116630311 | G | A | -0.0255 | 0.0022 | 4.10E-32 | 0.5633 | 0.0570 | 0.0768 | 4.58E-01 | 0.6657 | 0.0726 | 0.1392 | 6.02E-01 | 0.6657 |
| rs3213737 | 12 | 96379806 | A | G | -0.0319 | 0.0022 | 2.70E-49 | 0.5760 | 0.0595 | 0.0760 | 4.34E-01 | 0.6553 | 0.0583 | 0.1380 | 6.73E-01 | 0.6553 |
| rs35563099 | 10 | 119572403 | T | C | -0.0310 | 0.0029 | 1.80E-26 | 0.1640 | -0.0514 | 0.0939 | 5.84E-01 | 0.1849 | -0.1284 | 0.1704 | 4.51E-01 | 0.1849 |
| rs3759579 | 14 | 103851272 | G | A | 0.0124 | 0.0022 | 8.10E-09 | 0.5888 | -0.0324 | 0.0747 | 6.65E-01 | 0.6206 | -0.1026 | 0.1353 | 4.48E-01 | 0.6206 |
| rs41563 | 7 | 104852654 | A | G | 0.0135 | 0.0022 | 1.40E-09 | 0.3498 | 0.0328 | 0.0784 | 6.76E-01 | 0.3078 | -0.0817 | 0.1421 | 5.66E-01 | 0.3078 |
| rs4240559 | 6 | 98437775 | C | T | -0.0171 | 0.0021 | 1.40E-15 | 0.5579 | -0.0871 | 0.0757 | 2.50E-01 | 0.6514 | -0.2017 | 0.1375 | 1.42E-01 | 0.6514 |
| rs4272574 | 1 | 73661205 | T | C | 0.0152 | 0.0021 | 8.40E-13 | 0.4783 | -0.0113 | 0.0723 | 8.76E-01 | 0.5101 | 0.2547 | 0.1313 | 5.25E-02 | 0.5101 |
| rs4335021 | 6 | 32386619 | C | T | -0.0118 | 0.0022 | 4.90E-08 | 0.5997 | -0.0741 | 0.0883 | 4.01E-01 | 0.6420 | 0.0058 | 0.1605 | 9.71E-01 | 0.6420 |
| rs4438032 | 11 | 88786692 | G | C | 0.0232 | 0.0036 | 2.00E-10 | 0.9062 | 0.3118 | 0.1404 | 2.64E-02 | 0.9271 | 0.5264 | 0.2541 | 3.83E-02 | 0.9271 |
| rs4578351 | 11 | 16587580 | C | T | -0.0205 | 0.0026 | 1.70E-15 | 0.2220 | -0.0239 | 0.0843 | 7.77E-01 | 0.2496 | -0.0459 | 0.1531 | 7.64E-01 | 0.2496 |
| rs4670813 | 2 | 38317710 | A | G | -0.0127 | 0.0021 | 3.50E-09 | 0.4708 | -0.0515 | 0.0726 | 4.78E-01 | 0.5243 | 0.0893 | 0.1316 | 4.98E-01 | 0.5243 |
| rs4840542 | 8 | 10944809 | T | G | 0.0185 | 0.0021 | 4.00E-18 | 0.5058 | -0.0948 | 0.1413 | 5.02E-01 | 0.4261 | -0.0742 | 0.2559 | 7.72E-01 | 0.4261 |
| rs511515 | 6 | 33541507 | G | A | -0.0204 | 0.0023 | 1.60E-18 | 0.7009 | 0.1931 | 0.0873 | 2.70E-02 | 0.7820 | 0.1054 | 0.1588 | 5.07E-01 | 0.7820 |
| rs537894 | 3 | 138348595 | A | G | 0.0127 | 0.0022 | 5.00E-09 | 0.5490 | 0.0784 | 0.0780 | 3.15E-01 | 0.6724 | 0.3069 | 0.1414 | 3.00E-02 | 0.6724 |
| rs57994353 | 9 | 139356987 | C | T | 0.0127 | 0.0023 | 4.30E-08 | 0.2991 | 0.0432 | 0.0828 | 6.02E-01 | 0.2556 | 0.0734 | 0.1502 | 6.25E-01 | 0.2556 |
| rs6007506 | 22 | 45622014 | T | C | -0.0248 | 0.0023 | 4.10E-28 | 0.3374 | -0.1049 | 0.0753 | 1.64E-01 | 0.3559 | -0.1494 | 0.1367 | 2.74E-01 | 0.3559 |
| rs6059655 | 20 | 32665748 | G | A | -0.1273 | 0.0036 | 1.00E-200 | 0.8980 | -0.4034 | 0.2368 | 8.84E-02 | 0.9773 | -0.9753 | 0.4260 | 2.21E-02 | 0.9773 |
| rs61816766 | 1 | 152319572 | C | T | 0.0366 | 0.0062 | 3.40E-09 | 0.0323 | -0.3062 | 0.3891 | 4.31E-01 | 0.0079 | -0.0614 | 0.7052 | 9.31E-01 | 0.0079 |
| rs61981034 | 14 | 97377089 | A | G | 0.0133 | 0.0024 | 4.60E-08 | 0.2580 | -0.0749 | 0.0766 | 3.28E-01 | 0.3367 | -0.2270 | 0.1386 | 1.01E-01 | 0.3367 |
| rs6689641 | 1 | 110720400 | G | A | 0.0149 | 0.0021 | 2.60E-12 | 0.5426 | 0.0808 | 0.0729 | 2.67E-01 | 0.5587 | 0.0834 | 0.1321 | 5.28E-01 | 0.5587 |
| rs6882046 | 5 | 87968864 | G | A | 0.0144 | 0.0024 | 3.10E-09 | 0.2679 | -0.0866 | 0.0934 | 3.54E-01 | 0.1804 | -0.1883 | 0.1687 | 2.64E-01 | 0.1804 |
| rs699780 | 1 | 120455441 | G | A | -0.0184 | 0.0032 | 7.00E-09 | 0.1278 | 0.0029 | 0.0966 | 9.76E-01 | 0.1682 | 0.0329 | 0.1750 | 8.51E-01 | 0.1682 |
| rs72821630 | 2 | 63696212 | T | C | -0.0150 | 0.0025 | 9.00E-10 | 0.2514 | 0.0449 | 0.0754 | 5.51E-01 | 0.3625 | -0.0181 | 0.1365 | 8.94E-01 | 0.3625 |
| rs75300484 | 16 | 89489061 | T | C | -0.0342 | 0.0058 | 3.70E-09 | 0.0347 | -0.0415 | 0.2321 | 8.58E-01 | 0.0233 | -0.5149 | 0.4234 | 2.24E-01 | 0.0233 |
| rs75908072 | 6 | 290438 | C | T | -0.0318 | 0.0057 | 2.80E-08 | 0.0366 | -0.1864 | 0.2545 | 4.64E-01 | 0.0227 | -0.3066 | 0.4635 | 5.08E-01 | 0.0227 |
| rs7768317 | 6 | 41922220 | T | C | -0.0170 | 0.0025 | 4.90E-12 | 0.2489 | -0.0154 | 0.0825 | 8.52E-01 | 0.2621 | -0.0550 | 0.1496 | 7.13E-01 | 0.2621 |
| rs784235 | 18 | 53423144 | G | A | -0.0164 | 0.0028 | 3.00E-09 | 0.8191 | 0.2227 | 0.1023 | 2.95E-02 | 0.8551 | 0.1885 | 0.1862 | 3.11E-01 | 0.8551 |
| rs78444298 | 1 | 184672098 | A | G | -0.0467 | 0.0077 | 1.40E-09 | 0.0197 | -0.0538 | 0.2777 | 8.46E-01 | 0.0172 | -0.7791 | 0.5105 | 1.27E-01 | 0.0172 |
| rs849138 | 7 | 28177338 | A | G | 0.0126 | 0.0021 | 3.80E-09 | 0.5070 | 0.1477 | 0.0723 | 4.12E-02 | 0.4910 | 0.2574 | 0.1311 | 4.96E-02 | 0.4910 |
| rs9328259 | 6 | 508972 | A | C | 0.0267 | 0.0024 | 1.60E-29 | 0.7182 | -0.0844 | 0.0804 | 2.94E-01 | 0.7206 | 0.0291 | 0.1461 | 8.42E-01 | 0.7206 |
| rs964127 | 12 | 96290733 | T | A | 0.0123 | 0.0023 | 4.90E-08 | 0.3328 | 0.0127 | 0.0725 | 8.61E-01 | 0.4643 | -0.1716 | 0.1312 | 1.91E-01 | 0.4643 |
| rs9821675 | 3 | 49902544 | G | A | -0.0120 | 0.0021 | 1.50E-08 | 0.5053 | -0.0578 | 0.0740 | 4.35E-01 | 0.3949 | -0.2168 | 0.1343 | 1.07E-01 | 0.3949 |
| rs9832130 | 3 | 189194752 | A | G | -0.0128 | 0.0022 | 3.30E-09 | 0.5807 | 0.0435 | 0.0732 | 5.52E-01 | 0.5615 | -0.1607 | 0.1325 | 2.25E-01 | 0.5615 |
| rs9835772 | 3 | 85766025 | T | A | 0.0177 | 0.0025 | 8.60E-13 | 0.2437 | 0.1564 | 0.0859 | 6.88E-02 | 0.2285 | 0.2133 | 0.1556 | 1.71E-01 | 0.2285 |
| rs9858244 | 3 | 85787399 | A | G | 0.0189 | 0.0026 | 3.00E-13 | 0.2142 | 0.1651 | 0.0928 | 7.53E-02 | 0.1843 | 0.0858 | 0.1680 | 6.10E-01 | 0.1843 |
| rs9867857 | 3 | 156491160 | T | C | 0.0136 | 0.0021 | 1.80E-10 | 0.4892 | 0.0828 | 0.0731 | 2.57E-01 | 0.4296 | -0.0102 | 0.1326 | 9.39E-01 | 0.4296 |
| rs9904468 | 17 | 17573187 | G | C | 0.0137 | 0.0021 | 1.10E-10 | 0.5335 | -0.0161 | 0.0724 | 8.24E-01 | 0.5500 | 0.0474 | 0.1313 | 7.18E-01 | 0.5500 |

SNP: Single-nucleotide polymorphisms; Chr: Chromosome; EA: Effect allele; OA: Other allele; EAF: Effect allele frequency.

**Supplementary Table 5.** Information of identified SNPs in exposure (Childhood sunburn) and outcomes (overall Melanoma in situ and Melanoma in situ of face).

| SNP | | | | | Exposure (Childhood sunburn) | | | | Outcome (Melanoma in situ of trunk) | | | | Outcome (Melanoma in situ of lowerlimb) | | | | Outcome ( Melanoma in situ of upperlimb) | | | |
| --- | --- | --- | --- | --- | --- | --- | --- | --- | --- | --- | --- | --- | --- | --- | --- | --- | --- | --- | --- | --- |
| RS ID | Chr | Position | EA | OA | Beta | Se | P-  value | EAF | Beta | Se | P-  value | EAF | Beta | Se | P-  value | EAF | Beta | Se | P-value | EAF |
| rs10168349 | 2 | 46360907 | C | G | 0.0173 | 0.0023 | 1.40E-14 | 0.3357 | -0.1308 | 0.1536 | 3.94E-01 | 0.2825 | 0.1200 | 0.1582 | 4.48E-01 | 0.2821 | 0.0264 | 0.1682 | 8.75E-01 | 0.2821 |
| rs10202908 | 2 | 169378292 | T | C | -0.0126 | 0.0023 | 2.90E-08 | 0.6749 | -0.0267 | 0.1497 | 8.58E-01 | 0.6989 | -0.0983 | 0.1557 | 5.28E-01 | 0.6990 | -0.3785 | 0.1641 | 2.11E-02 | 0.6990 |
| rs10220751 | 15 | 47923520 | G | T | -0.0137 | 0.0022 | 2.50E-10 | 0.4048 | -0.1945 | 0.1431 | 1.74E-01 | 0.3599 | 0.0317 | 0.1473 | 8.30E-01 | 0.3600 | 0.1108 | 0.1562 | 4.78E-01 | 0.3600 |
| rs10788627 | 10 | 82203069 | C | T | 0.0120 | 0.0021 | 1.60E-08 | 0.4745 | -0.1141 | 0.1383 | 4.10E-01 | 0.5463 | 0.0005 | 0.1432 | 9.97E-01 | 0.5464 | 0.0325 | 0.1506 | 8.29E-01 | 0.5464 |
| rs10810636 | 9 | 16799109 | G | A | 0.0275 | 0.0025 | 2.80E-28 | 0.7603 | -0.0230 | 0.1567 | 8.83E-01 | 0.7369 | 0.2487 | 0.1627 | 1.26E-01 | 0.7367 | -0.0097 | 0.1710 | 9.55E-01 | 0.7367 |
| rs10873552 | 14 | 105433129 | G | A | 0.0124 | 0.0022 | 3.70E-08 | 0.6577 | 0.0449 | 0.1396 | 7.48E-01 | 0.5732 | 0.2357 | 0.1444 | 1.03E-01 | 0.5721 | -0.0372 | 0.1526 | 8.07E-01 | 0.5721 |
| rs10896139 | 11 | 66650060 | T | C | -0.0136 | 0.0024 | 1.40E-08 | 0.2709 | 0.0277 | 0.1536 | 8.57E-01 | 0.2792 | -0.0259 | 0.1590 | 8.71E-01 | 0.2797 | -0.1014 | 0.1682 | 5.47E-01 | 0.2797 |
| rs11070811 | 15 | 31394082 | T | C | -0.0176 | 0.0027 | 1.50E-10 | 0.1832 | -0.3709 | 0.2005 | 6.43E-02 | 0.1331 | -0.1906 | 0.2061 | 3.55E-01 | 0.1336 | -0.0673 | 0.2198 | 7.60E-01 | 0.1336 |
| rs11104733 | 12 | 88490119 | T | C | 0.0492 | 0.0089 | 2.80E-08 | 0.0147 | 0.5297 | 0.4681 | 2.58E-01 | 0.0235 | 0.0718 | 0.4700 | 8.79E-01 | 0.0235 | -0.7720 | 0.5067 | 1.28E-01 | 0.0235 |
| rs111391498 | 4 | 1341553 | G | A | -0.0474 | 0.0050 | 1.70E-21 | 0.0479 | -0.3274 | 0.4141 | 4.29E-01 | 0.0288 | -0.1159 | 0.4273 | 7.86E-01 | 0.0288 | -0.0303 | 0.4510 | 9.46E-01 | 0.0288 |
| rs111650620 | 20 | 34591725 | A | G | 0.6618 | 0.0601 | 3.20E-28 | 0.0022 | 0.0867 | 0.4260 | 8.39E-01 | 0.0272 | -0.4479 | 0.4464 | 3.16E-01 | 0.0272 | -0.0031 | 0.4552 | 9.95E-01 | 0.0272 |
| rs112089506 | 16 | 90149171 | T | C | -0.0581 | 0.0040 | 1.40E-47 | 0.0812 | -0.3903 | 0.2394 | 1.03E-01 | 0.0934 | -0.1858 | 0.2444 | 4.47E-01 | 0.0939 | -0.1332 | 0.2579 | 6.06E-01 | 0.0939 |
| rs11242899 | 6 | 460302 | A | G | -0.0245 | 0.0024 | 3.20E-24 | 0.2663 | -0.1522 | 0.1570 | 3.32E-01 | 0.2626 | 0.1461 | 0.1625 | 3.69E-01 | 0.2612 | -0.0734 | 0.1716 | 6.69E-01 | 0.2612 |
| rs1126809 | 11 | 89017961 | A | G | 0.0825 | 0.0023 | 1.00E-200 | 0.3028 | 0.1547 | 0.1781 | 3.85E-01 | 0.1784 | 0.1450 | 0.1848 | 4.33E-01 | 0.1790 | 0.1975 | 0.1953 | 3.12E-01 | 0.1790 |
| rs116125333 | 5 | 33930012 | G | T | -0.0397 | 0.0072 | 3.40E-08 | 0.0232 | -0.2249 | 0.2562 | 3.80E-01 | 0.0842 | -0.3206 | 0.2651 | 2.26E-01 | 0.0848 | 0.1942 | 0.2793 | 4.87E-01 | 0.0848 |
| rs11648436 | 16 | 14008674 | T | C | -0.0273 | 0.0022 | 9.90E-35 | 0.3564 | 0.0348 | 0.1494 | 8.16E-01 | 0.3079 | -0.1345 | 0.1550 | 3.86E-01 | 0.3070 | 0.1437 | 0.1635 | 3.79E-01 | 0.3070 |
| rs117132860 | 7 | 17134708 | A | G | 0.0593 | 0.0067 | 1.20E-18 | 0.0256 | 0.1665 | 1.0908 | 8.79E-01 | 0.0040 | -1.1095 | 1.2060 | 3.58E-01 | 0.0040 | 3.1897 | 1.2696 | 1.20E-02 | 0.0040 |
| rs11739906 | 5 | 59019359 | C | A | 0.0130 | 0.0023 | 8.40E-09 | 0.3275 | 0.0467 | 0.1606 | 7.71E-01 | 0.2405 | 0.0860 | 0.1653 | 6.03E-01 | 0.2405 | 0.0503 | 0.1752 | 7.74E-01 | 0.2405 |
| rs117462393 | 16 | 14277804 | T | C | 0.0815 | 0.0096 | 1.50E-17 | 0.0144 | 0.0455 | 0.2863 | 8.74E-01 | 0.0648 | -0.2269 | 0.2940 | 4.40E-01 | 0.0652 | -0.1799 | 0.3071 | 5.58E-01 | 0.0652 |
| rs12203592 | 6 | 396321 | T | C | 0.1531 | 0.0025 | 1.00E-200 | 0.2191 | 0.3783 | 0.3963 | 3.40E-01 | 0.0307 | 0.2366 | 0.4127 | 5.66E-01 | 0.0312 | -0.3685 | 0.4375 | 4.00E-01 | 0.0312 |
| rs1233578 | 6 | 28712247 | G | A | -0.0206 | 0.0028 | 1.50E-13 | 0.1764 | -0.4341 | 0.2660 | 1.03E-01 | 0.0611 | -0.8300 | 0.3275 | 1.13E-02 | 0.0615 | 0.4578 | 0.3017 | 1.29E-01 | 0.0615 |
| rs12350739 | 9 | 16885017 | A | G | 0.0355 | 0.0022 | 5.80E-59 | 0.6068 | -0.1913 | 0.1379 | 1.65E-01 | 0.5454 | -0.0033 | 0.1428 | 9.81E-01 | 0.5451 | 0.2823 | 0.1504 | 6.04E-02 | 0.5451 |
| rs1260326 | 2 | 27730940 | C | T | -0.0125 | 0.0022 | 8.10E-09 | 0.6040 | 0.0193 | 0.1448 | 8.94E-01 | 0.6493 | -0.2735 | 0.1501 | 6.85E-02 | 0.6502 | -0.1381 | 0.1581 | 3.82E-01 | 0.6502 |
| rs1267038 | 2 | 162099153 | C | A | -0.0179 | 0.0030 | 2.20E-09 | 0.8449 | 0.1437 | 0.1797 | 4.24E-01 | 0.8185 | 0.3581 | 0.1870 | 5.54E-02 | 0.8189 | -0.0844 | 0.1963 | 6.67E-01 | 0.8189 |
| rs1278766 | 13 | 113534382 | C | T | 0.0191 | 0.0021 | 3.90E-19 | 0.5457 | 0.1599 | 0.1379 | 2.46E-01 | 0.4879 | -0.0048 | 0.1426 | 9.73E-01 | 0.4868 | -0.2007 | 0.1512 | 1.84E-01 | 0.4868 |
| rs12913832 | 15 | 28365618 | G | A | 0.0605 | 0.0025 | 1.90E-126 | 0.7753 | -0.4113 | 0.2284 | 7.18E-02 | 0.8942 | -0.1807 | 0.2403 | 4.52E-01 | 0.8957 | -0.6347 | 0.2535 | 1.23E-02 | 0.8957 |
| rs1308048 | 1 | 66888542 | C | T | -0.0204 | 0.0022 | 5.30E-21 | 0.4195 | -0.3128 | 0.1397 | 2.51E-02 | 0.4293 | -0.1212 | 0.1438 | 3.99E-01 | 0.4294 | 0.0806 | 0.1524 | 5.97E-01 | 0.4294 |
| rs1326798 | 9 | 12722227 | G | C | 0.0121 | 0.0022 | 3.60E-08 | 0.6206 | -0.1670 | 0.1431 | 2.43E-01 | 0.6355 | 0.3456 | 0.1479 | 1.94E-02 | 0.6360 | -0.0191 | 0.1563 | 9.03E-01 | 0.6360 |
| rs13332673 | 16 | 89940386 | T | G | -0.1021 | 0.0105 | 3.30E-22 | 0.0102 | 0.2969 | 0.8132 | 7.15E-01 | 0.0074 | -0.2337 | 0.9063 | 7.97E-01 | 0.0067 | -0.0360 | 0.9666 | 9.70E-01 | 0.0067 |
| rs139414522 | 16 | 89821155 | C | T | -0.0784 | 0.0091 | 5.40E-18 | 0.0153 | 0.0517 | 0.7263 | 9.43E-01 | 0.0093 | 0.6791 | 0.7549 | 3.68E-01 | 0.0092 | -0.4126 | 0.7701 | 5.92E-01 | 0.0092 |
| rs141817469 | 16 | 89927151 | T | C | -0.0932 | 0.0058 | 1.00E-57 | 0.0351 | -0.5240 | 0.4050 | 1.96E-01 | 0.0316 | -0.2429 | 0.4168 | 5.60E-01 | 0.0319 | 0.2889 | 0.4377 | 5.09E-01 | 0.0319 |
| rs142314514 | 16 | 90130996 | G | A | -0.0816 | 0.0070 | 3.60E-31 | 0.0285 | -0.0142 | 0.4304 | 9.74E-01 | 0.0280 | 0.0303 | 0.4474 | 9.46E-01 | 0.0278 | 0.0050 | 0.4656 | 9.92E-01 | 0.0278 |
| rs1437635 | 11 | 16358722 | A | C | 0.0207 | 0.0029 | 5.20E-13 | 0.1660 | -0.2082 | 0.2171 | 3.38E-01 | 0.1156 | -0.1243 | 0.2235 | 5.78E-01 | 0.1156 | -0.2607 | 0.2377 | 2.73E-01 | 0.1156 |
| rs150527451 | 11 | 68817897 | A | G | 0.0262 | 0.0035 | 4.00E-14 | 0.1064 | 0.2294 | 0.1652 | 1.65E-01 | 0.2348 | -0.1356 | 0.1704 | 4.26E-01 | 0.2345 | -0.0446 | 0.1787 | 8.03E-01 | 0.2345 |
| rs151337382 | 20 | 34204925 | A | T | 0.0575 | 0.0093 | 5.20E-10 | 0.0136 | -0.9356 | 1.0114 | 3.55E-01 | 0.0045 | -1.0471 | 1.1093 | 3.45E-01 | 0.0045 | -0.1616 | 1.1958 | 8.92E-01 | 0.0045 |
| rs1548714 | 17 | 26280204 | C | A | 0.0162 | 0.0027 | 3.00E-09 | 0.8117 | -0.0999 | 0.1760 | 5.70E-01 | 0.8124 | -0.3024 | 0.1821 | 9.68E-02 | 0.8125 | -0.2363 | 0.1921 | 2.19E-01 | 0.8125 |
| rs16891982 | 5 | 33951693 | G | C | 0.2292 | 0.0062 | 1.00E-200 | 0.9709 | 0.1242 | 0.5332 | 8.16E-01 | 0.9821 | 0.3954 | 0.6034 | 5.12E-01 | 0.9834 | 0.5461 | 0.6368 | 3.91E-01 | 0.9834 |
| rs17232484 | 16 | 89861650 | A | G | 0.0610 | 0.0091 | 1.80E-11 | 0.0142 | -0.2412 | 0.5382 | 6.54E-01 | 0.0199 | 0.2522 | 0.5584 | 6.51E-01 | 0.0201 | 0.0724 | 0.5877 | 9.02E-01 | 0.0201 |
| rs1805007 | 16 | 89986117 | T | C | 0.1964 | 0.0035 | 1.00E-200 | 0.1009 | 0.4332 | 0.2783 | 1.20E-01 | 0.0667 | 0.3355 | 0.2918 | 2.50E-01 | 0.0663 | 0.4998 | 0.3062 | 1.03E-01 | 0.0663 |
| rs1805008 | 16 | 89986144 | T | C | 0.1195 | 0.0038 | 1.00E-200 | 0.0866 | 0.3572 | 0.2657 | 1.79E-01 | 0.0687 | -0.0020 | 0.2773 | 9.94E-01 | 0.0688 | -0.1331 | 0.2941 | 6.51E-01 | 0.0688 |
| rs1989483 | 7 | 16942661 | G | A | 0.0125 | 0.0022 | 1.20E-08 | 0.3891 | -0.0525 | 0.1425 | 7.12E-01 | 0.3690 | 0.0809 | 0.1473 | 5.83E-01 | 0.3698 | 0.1786 | 0.1558 | 2.51E-01 | 0.3698 |
| rs2299098 | 7 | 24756377 | C | G | 0.0148 | 0.0027 | 3.60E-08 | 0.1962 | 0.2684 | 0.1891 | 1.56E-01 | 0.1559 | -0.2810 | 0.1956 | 1.51E-01 | 0.1555 | -0.4628 | 0.2066 | 2.51E-02 | 0.1555 |
| rs251468 | 5 | 149194485 | T | C | -0.0282 | 0.0025 | 4.40E-30 | 0.2484 | 0.0453 | 0.1458 | 7.56E-01 | 0.3441 | -0.2242 | 0.1504 | 1.36E-01 | 0.3435 | -0.0277 | 0.1589 | 8.61E-01 | 0.3435 |
| rs2737217 | 8 | 116630311 | G | A | -0.0255 | 0.0022 | 4.10E-32 | 0.5633 | -0.0726 | 0.1461 | 6.19E-01 | 0.6657 | 0.3009 | 0.1515 | 4.71E-02 | 0.6646 | 0.0943 | 0.1593 | 5.54E-01 | 0.6646 |
| rs3213737 | 12 | 96379806 | A | G | -0.0319 | 0.0022 | 2.70E-49 | 0.5760 | 0.0001 | 0.1449 | 9.99E-01 | 0.6553 | 0.0732 | 0.1500 | 6.25E-01 | 0.6552 | -0.0619 | 0.1582 | 6.95E-01 | 0.6552 |
| rs35563099 | 10 | 119572403 | T | C | -0.0310 | 0.0029 | 1.80E-26 | 0.1640 | 0.0277 | 0.1791 | 8.77E-01 | 0.1849 | -0.3424 | 0.1872 | 6.74E-02 | 0.1837 | -0.4271 | 0.1943 | 2.80E-02 | 0.1837 |
| rs3759579 | 14 | 103851272 | G | A | 0.0124 | 0.0022 | 8.10E-09 | 0.5888 | -0.0831 | 0.1418 | 5.58E-01 | 0.6206 | 0.0038 | 0.1472 | 9.79E-01 | 0.6198 | -0.1345 | 0.1549 | 3.85E-01 | 0.6198 |
| rs41563 | 7 | 104852654 | A | G | 0.0135 | 0.0022 | 1.40E-09 | 0.3498 | 0.0708 | 0.1497 | 6.36E-01 | 0.3078 | -0.0498 | 0.1544 | 7.47E-01 | 0.3071 | -0.2001 | 0.1626 | 2.18E-01 | 0.3071 |
| rs4240559 | 6 | 98437775 | C | T | -0.0171 | 0.0021 | 1.40E-15 | 0.5579 | -0.1583 | 0.1442 | 2.72E-01 | 0.6514 | 0.1109 | 0.1491 | 4.57E-01 | 0.6521 | 0.0792 | 0.1574 | 6.15E-01 | 0.6521 |
| rs4272574 | 1 | 73661205 | T | C | 0.0152 | 0.0021 | 8.40E-13 | 0.4783 | -0.1393 | 0.1378 | 3.12E-01 | 0.5101 | -0.1321 | 0.1428 | 3.55E-01 | 0.5098 | 0.0430 | 0.1506 | 7.75E-01 | 0.5098 |
| rs4335021 | 6 | 32386619 | C | T | -0.0118 | 0.0022 | 4.90E-08 | 0.5997 | -0.3808 | 0.1680 | 2.34E-02 | 0.6420 | 0.0325 | 0.1475 | 8.26E-01 | 0.6412 | -0.2342 | 0.1562 | 1.34E-01 | 0.6412 |
| rs4438032 | 11 | 88786692 | G | C | 0.0232 | 0.0036 | 2.00E-10 | 0.9062 | 0.3426 | 0.2662 | 1.98E-01 | 0.9271 | 0.1865 | 0.2782 | 5.03E-01 | 0.9272 | -0.6480 | 0.2896 | 2.52E-02 | 0.9272 |
| rs4578351 | 11 | 16587580 | C | T | -0.0205 | 0.0026 | 1.70E-15 | 0.2220 | -0.1113 | 0.1610 | 4.89E-01 | 0.2496 | 0.0308 | 0.1686 | 8.55E-01 | 0.2474 | -0.1341 | 0.1758 | 4.45E-01 | 0.2474 |
| rs4670813 | 2 | 38317710 | A | G | -0.0127 | 0.0021 | 3.50E-09 | 0.4708 | -0.1170 | 0.1380 | 3.96E-01 | 0.5243 | -0.2116 | 0.1438 | 1.41E-01 | 0.5242 | 0.0990 | 0.1512 | 5.12E-01 | 0.5242 |
| rs4840542 | 8 | 10944809 | T | G | 0.0185 | 0.0021 | 4.00E-18 | 0.5058 | 0.0824 | 0.2641 | 7.55E-01 | 0.4261 | -0.3183 | 0.1445 | 2.77E-02 | 0.4270 | 0.0010 | 0.1525 | 9.95E-01 | 0.4270 |
| rs511515 | 6 | 33541507 | G | A | -0.0204 | 0.0023 | 1.60E-18 | 0.7009 | 0.2389 | 0.1647 | 1.47E-01 | 0.7820 | 0.3073 | 0.1718 | 7.37E-02 | 0.7814 | 0.0439 | 0.1817 | 8.09E-01 | 0.7814 |
| rs537894 | 3 | 138348595 | A | G | 0.0127 | 0.0022 | 5.00E-09 | 0.5490 | -0.1426 | 0.1488 | 3.38E-01 | 0.6724 | 0.0967 | 0.1537 | 5.29E-01 | 0.6732 | 0.3750 | 0.1626 | 2.11E-02 | 0.6732 |
| rs57994353 | 9 | 139356987 | C | T | 0.0127 | 0.0023 | 4.30E-08 | 0.2991 | -0.0775 | 0.1573 | 6.22E-01 | 0.2556 | -0.1030 | 0.1632 | 5.28E-01 | 0.2551 | -0.0082 | 0.1722 | 9.62E-01 | 0.2551 |
| rs6007506 | 22 | 45622014 | T | C | -0.0248 | 0.0023 | 4.10E-28 | 0.3374 | -0.2756 | 0.1438 | 5.52E-02 | 0.3559 | 0.0192 | 0.1482 | 8.97E-01 | 0.3559 | -0.1705 | 0.1565 | 2.76E-01 | 0.3559 |
| rs6059655 | 20 | 32665748 | G | A | -0.1273 | 0.0036 | 1.00E-200 | 0.8980 | -0.5908 | 0.4467 | 1.86E-01 | 0.9773 | 0.8117 | 0.4726 | 8.59E-02 | 0.9775 | -1.0049 | 0.5057 | 4.69E-02 | 0.9775 |
| rs61816766 | 1 | 152319572 | C | T | 0.0366 | 0.0062 | 3.40E-09 | 0.0323 | -0.9997 | 0.7328 | 1.73E-01 | 0.0079 | 0.1090 | 0.7578 | 8.86E-01 | 0.0079 | -1.0309 | 0.8228 | 2.10E-01 | 0.0079 |
| rs61981034 | 14 | 97377089 | A | G | 0.0133 | 0.0024 | 4.60E-08 | 0.2580 | -0.0639 | 0.1458 | 6.61E-01 | 0.3367 | 0.3609 | 0.1513 | 1.71E-02 | 0.3376 | 0.0008 | 0.1591 | 9.96E-01 | 0.3376 |
| rs6689641 | 1 | 110720400 | G | A | 0.0149 | 0.0021 | 2.60E-12 | 0.5426 | 0.2115 | 0.1388 | 1.27E-01 | 0.5587 | 0.0541 | 0.1437 | 7.07E-01 | 0.5590 | 0.1422 | 0.1516 | 3.48E-01 | 0.5590 |
| rs6882046 | 5 | 87968864 | G | A | 0.0144 | 0.0024 | 3.10E-09 | 0.2679 | -0.0908 | 0.1767 | 6.07E-01 | 0.1804 | 0.1253 | 0.1851 | 4.98E-01 | 0.1807 | 0.0367 | 0.1948 | 8.51E-01 | 0.1807 |
| rs699780 | 1 | 120455441 | G | A | -0.0184 | 0.0032 | 7.00E-09 | 0.1278 | -0.1617 | 0.1844 | 3.80E-01 | 0.1682 | -0.2655 | 0.1903 | 1.63E-01 | 0.1684 | -0.2220 | 0.2006 | 2.68E-01 | 0.1684 |
| rs72821630 | 2 | 63696212 | T | C | -0.0150 | 0.0025 | 9.00E-10 | 0.2514 | 0.1040 | 0.1440 | 4.70E-01 | 0.3625 | 0.0536 | 0.1492 | 7.19E-01 | 0.3624 | -0.0900 | 0.1568 | 5.66E-01 | 0.3624 |
| rs75300484 | 16 | 89489061 | T | C | -0.0342 | 0.0058 | 3.70E-09 | 0.0347 | -0.6275 | 0.4395 | 1.53E-01 | 0.0233 | -0.0413 | 0.4466 | 9.26E-01 | 0.0234 | 0.6421 | 0.4768 | 1.78E-01 | 0.0234 |
| rs75908072 | 6 | 290438 | C | T | -0.0318 | 0.0057 | 2.80E-08 | 0.0366 | 0.1459 | 0.4843 | 7.63E-01 | 0.0227 | 0.0111 | 0.4933 | 9.82E-01 | 0.0232 | -0.5846 | 0.5484 | 2.86E-01 | 0.0232 |
| rs7768317 | 6 | 41922220 | T | C | -0.0170 | 0.0025 | 4.90E-12 | 0.2489 | -0.0670 | 0.1571 | 6.70E-01 | 0.2621 | 0.2052 | 0.1627 | 2.07E-01 | 0.2617 | 0.0573 | 0.1719 | 7.39E-01 | 0.2617 |
| rs784235 | 18 | 53423144 | G | A | -0.0164 | 0.0028 | 3.00E-09 | 0.8191 | 0.1636 | 0.1949 | 4.01E-01 | 0.8551 | 0.0472 | 0.2019 | 8.15E-01 | 0.8556 | 0.4931 | 0.2135 | 2.09E-02 | 0.8556 |
| rs78444298 | 1 | 184672098 | A | G | -0.0467 | 0.0077 | 1.40E-09 | 0.0197 | -0.7173 | 0.5327 | 1.78E-01 | 0.0172 | 0.4070 | 0.5343 | 4.46E-01 | 0.0174 | 0.6565 | 0.5829 | 2.60E-01 | 0.0174 |
| rs849138 | 7 | 28177338 | A | G | 0.0126 | 0.0021 | 3.80E-09 | 0.5070 | 0.1365 | 0.1380 | 3.23E-01 | 0.4910 | 0.0764 | 0.1426 | 5.92E-01 | 0.4910 | 0.1223 | 0.1503 | 4.16E-01 | 0.4910 |
| rs9328259 | 6 | 508972 | A | C | 0.0267 | 0.0024 | 1.60E-29 | 0.7182 | -0.1877 | 0.1529 | 2.20E-01 | 0.7206 | 0.2735 | 0.1589 | 8.52E-02 | 0.7206 | 0.0826 | 0.1672 | 6.21E-01 | 0.7206 |
| rs964127 | 12 | 96290733 | T | A | 0.0123 | 0.0023 | 4.90E-08 | 0.3328 | -0.0144 | 0.1382 | 9.17E-01 | 0.4643 | 0.0169 | 0.1434 | 9.06E-01 | 0.4648 | 0.1510 | 0.1511 | 3.18E-01 | 0.4648 |
| rs9821675 | 3 | 49902544 | G | A | -0.0120 | 0.0021 | 1.50E-08 | 0.5053 | -0.0292 | 0.1407 | 8.36E-01 | 0.3949 | -0.1093 | 0.1456 | 4.53E-01 | 0.3949 | 0.0233 | 0.1537 | 8.79E-01 | 0.3949 |
| rs9832130 | 3 | 189194752 | A | G | -0.0128 | 0.0022 | 3.30E-09 | 0.5807 | -0.0668 | 0.1391 | 6.31E-01 | 0.5615 | 0.0899 | 0.1450 | 5.35E-01 | 0.5637 | 0.1752 | 0.1525 | 2.51E-01 | 0.5637 |
| rs9835772 | 3 | 85766025 | T | A | 0.0177 | 0.0025 | 8.60E-13 | 0.2437 | 0.1939 | 0.1639 | 2.37E-01 | 0.2285 | 0.2428 | 0.1694 | 1.52E-01 | 0.2289 | 0.3091 | 0.1791 | 8.44E-02 | 0.2289 |
| rs9858244 | 3 | 85787399 | A | G | 0.0189 | 0.0026 | 3.00E-13 | 0.2142 | 0.1883 | 0.1770 | 2.88E-01 | 0.1843 | 0.2277 | 0.1823 | 2.12E-01 | 0.1847 | 0.2787 | 0.1931 | 1.49E-01 | 0.1847 |
| rs9867857 | 3 | 156491160 | T | C | 0.0136 | 0.0021 | 1.80E-10 | 0.4892 | 0.2372 | 0.1393 | 8.85E-02 | 0.4296 | -0.0911 | 0.1441 | 5.27E-01 | 0.4290 | -0.1000 | 0.1514 | 5.09E-01 | 0.4290 |
| rs9904468 | 17 | 17573187 | G | C | 0.0137 | 0.0021 | 1.10E-10 | 0.5335 | -0.0096 | 0.1380 | 9.44E-01 | 0.5500 | 0.0452 | 0.1433 | 7.52E-01 | 0.5497 | 0.0758 | 0.1506 | 6.15E-01 | 0.5497 |

SNP: Single-nucleotide polymorphisms; Chr: Chromosome; EA: Effect allele; OA: Other allele; EAF: Effect allele frequency.

**Supplementary Table 6.** Information of identified SNPs in exposure (Childhood sunburn) and outcomes(skin carcinoma with all other cancers excluded ).

| SNP | | | | | Exposure (Childhood sunburn) | | | | Outcome (Malignant melanoma of skin, controls excluding all cancers) | | | | Outcome (Non-melanoma skin cancer, controls excluding all cancers) | | | |
| --- | --- | --- | --- | --- | --- | --- | --- | --- | --- | --- | --- | --- | --- | --- | --- | --- |
| RS ID | Chr | Position | EA | OA | Beta | Se | P-value | EAF | Beta | Se | P-value | EAF | Beta | Se | P-value | EAF |
| rs10168349 | 2 | 46360907 | C | G | 0.0173 | 0.0023 | 1.40E-14 | 0.3357 | -0.0002 | 0.1596 | 9.99E-01 | 0.2822 | 0.0219 | 0.0177 | 2.15E-01 | 0.2824 |
| rs10202908 | 2 | 169378292 | T | C | -0.0126 | 0.0023 | 2.90E-08 | 0.6749 | 0.2495 | 0.1565 | 1.11E-01 | 0.6990 | 0.0127 | 0.0173 | 4.65E-01 | 0.6991 |
| rs10220751 | 15 | 47923520 | G | T | -0.0137 | 0.0022 | 2.50E-10 | 0.4048 | 0.0376 | 0.1487 | 8.01E-01 | 0.3600 | -0.0163 | 0.0165 | 3.23E-01 | 0.3598 |
| rs10788627 | 10 | 82203069 | C | T | 0.0120 | 0.0021 | 1.60E-08 | 0.4745 | 0.0019 | 0.1436 | 9.89E-01 | 0.5468 | 0.0192 | 0.0159 | 2.29E-01 | 0.5471 |
| rs10810636 | 9 | 16799109 | G | A | 0.0275 | 0.0025 | 2.80E-28 | 0.7603 | 0.0410 | 0.1628 | 8.01E-01 | 0.7365 | 0.0734 | 0.0181 | 4.80E-05 | 0.7373 |
| rs10873552 | 14 | 105433129 | G | A | 0.0124 | 0.0022 | 3.70E-08 | 0.6577 | -0.0740 | 0.1449 | 6.10E-01 | 0.5739 | -0.0121 | 0.0161 | 4.50E-01 | 0.5737 |
| rs10896139 | 11 | 66650060 | T | C | -0.0136 | 0.0024 | 1.40E-08 | 0.2709 | -0.1792 | 0.1594 | 2.61E-01 | 0.2780 | 0.0193 | 0.0177 | 2.76E-01 | 0.2783 |
| rs11070811 | 15 | 31394082 | T | C | -0.0176 | 0.0027 | 1.50E-10 | 0.1832 | -0.1218 | 0.2092 | 5.61E-01 | 0.1333 | -0.0527 | 0.0233 | 2.36E-02 | 0.1331 |
| rs11104733 | 12 | 88490119 | T | C | 0.0492 | 0.0089 | 2.80E-08 | 0.0147 | -0.3342 | 0.4822 | 4.88E-01 | 0.0235 | 0.0787 | 0.0526 | 1.35E-01 | 0.0236 |
| rs111391498 | 4 | 1341553 | G | A | -0.0474 | 0.0050 | 1.70E-21 | 0.0479 | -0.3016 | 0.4299 | 4.83E-01 | 0.0286 | 0.0516 | 0.0472 | 2.74E-01 | 0.0287 |
| rs111650620 | 20 | 34591725 | A | G | 0.6618 | 0.0601 | 3.20E-28 | 0.0022 | -0.2473 | 0.4434 | 5.77E-01 | 0.0273 | 0.0131 | 0.0483 | 7.87E-01 | 0.0273 |
| rs112089506 | 16 | 90149171 | T | C | -0.0581 | 0.0040 | 1.40E-47 | 0.0812 | -0.1878 | 0.2470 | 4.47E-01 | 0.0939 | -0.1320 | 0.0275 | 1.63E-06 | 0.0934 |
| rs11242899 | 6 | 460302 | A | G | -0.0245 | 0.0024 | 3.20E-24 | 0.2663 | 0.0553 | 0.1628 | 7.34E-01 | 0.2624 | 0.0211 | 0.0181 | 2.43E-01 | 0.2626 |
| rs1126809 | 11 | 89017961 | A | G | 0.0825 | 0.0023 | 1.00E-200 | 0.3028 | -0.0919 | 0.1868 | 6.23E-01 | 0.1765 | 0.1648 | 0.0208 | 2.37E-15 | 0.1779 |
| rs116125333 | 5 | 33930012 | G | T | -0.0397 | 0.0072 | 3.40E-08 | 0.0232 | -0.0195 | 0.2633 | 9.41E-01 | 0.0842 | -0.0268 | 0.0289 | 3.54E-01 | 0.0841 |
| rs11648436 | 16 | 14008674 | T | C | -0.0273 | 0.0022 | 9.90E-35 | 0.3564 | -0.0698 | 0.1556 | 6.54E-01 | 0.3077 | -0.0010 | 0.0172 | 9.55E-01 | 0.3077 |
| rs117132860 | 7 | 17134708 | A | G | 0.0593 | 0.0067 | 1.20E-18 | 0.0256 | 2.0444 | 1.1811 | 8.35E-02 | 0.0039 | 0.4590 | 0.1287 | 3.63E-04 | 0.0040 |
| rs11739906 | 5 | 59019359 | C | A | 0.0130 | 0.0023 | 8.40E-09 | 0.3275 | -0.0004 | 0.1669 | 9.98E-01 | 0.2401 | 0.0041 | 0.0186 | 8.24E-01 | 0.2402 |
| rs117462393 | 16 | 14277804 | T | C | 0.0815 | 0.0096 | 1.50E-17 | 0.0144 | -0.0191 | 0.2933 | 9.48E-01 | 0.0651 | -0.0244 | 0.0327 | 4.56E-01 | 0.0650 |
| rs12203592 | 6 | 396321 | T | C | 0.1531 | 0.0025 | 1.00E-200 | 0.2191 | 0.1575 | 0.4250 | 7.11E-01 | 0.0301 | 0.3746 | 0.0477 | 4.29E-15 | 0.0306 |
| rs1233578 | 6 | 28712247 | G | A | -0.0206 | 0.0028 | 1.50E-13 | 0.1764 | -0.7421 | 0.3115 | 1.72E-02 | 0.0604 | 0.0496 | 0.0341 | 1.45E-01 | 0.0606 |
| rs12350739 | 9 | 16885017 | A | G | 0.0355 | 0.0022 | 5.80E-59 | 0.6068 | -0.1895 | 0.1436 | 1.87E-01 | 0.5454 | 0.0769 | 0.0159 | 1.40E-06 | 0.5465 |
| rs1260326 | 2 | 27730940 | C | T | -0.0125 | 0.0022 | 8.10E-09 | 0.6040 | 0.0794 | 0.1500 | 5.96E-01 | 0.6491 | 0.0072 | 0.0166 | 6.65E-01 | 0.6492 |
| rs1267038 | 2 | 162099153 | C | A | -0.0179 | 0.0030 | 2.20E-09 | 0.8449 | -0.0314 | 0.1860 | 8.66E-01 | 0.8187 | 0.0361 | 0.0207 | 8.10E-02 | 0.8190 |
| rs1278766 | 13 | 113534382 | C | T | 0.0191 | 0.0021 | 3.90E-19 | 0.5457 | -0.1373 | 0.1434 | 3.38E-01 | 0.4873 | 0.0511 | 0.0159 | 1.30E-03 | 0.4880 |
| rs12913832 | 15 | 28365618 | G | A | 0.0605 | 0.0025 | 1.90E-126 | 0.7753 | -0.0358 | 0.2358 | 8.79E-01 | 0.8944 | -0.1461 | 0.0260 | 1.90E-08 | 0.8937 |
| rs1308048 | 1 | 66888542 | C | T | -0.0204 | 0.0022 | 5.30E-21 | 0.4195 | 0.1589 | 0.1447 | 2.72E-01 | 0.4299 | -0.0553 | 0.0160 | 5.60E-04 | 0.4291 |
| rs1326798 | 9 | 12722227 | G | C | 0.0121 | 0.0022 | 3.60E-08 | 0.6206 | 0.5467 | 0.1502 | 2.73E-04 | 0.6349 | 0.0714 | 0.0165 | 1.51E-05 | 0.6356 |
| rs13332673 | 16 | 89940386 | T | G | -0.1021 | 0.0105 | 3.30E-22 | 0.0102 | -1.0617 | 0.8631 | 2.19E-01 | 0.0073 | -0.0103 | 0.0951 | 9.14E-01 | 0.0073 |
| rs139414522 | 16 | 89821155 | C | T | -0.0784 | 0.0091 | 5.40E-18 | 0.0153 | 0.6775 | 0.7658 | 3.76E-01 | 0.0093 | 0.1176 | 0.0830 | 1.57E-01 | 0.0093 |
| rs141817469 | 16 | 89927151 | T | C | -0.0932 | 0.0058 | 1.00E-57 | 0.0351 | 0.1287 | 0.4109 | 7.54E-01 | 0.0319 | -0.2008 | 0.0462 | 1.36E-05 | 0.0316 |
| rs142314514 | 16 | 90130996 | G | A | -0.0816 | 0.0070 | 3.60E-31 | 0.0285 | 0.0646 | 0.4454 | 8.85E-01 | 0.0281 | -0.1830 | 0.0506 | 2.99E-04 | 0.0280 |
| rs1437635 | 11 | 16358722 | A | C | 0.0207 | 0.0029 | 5.20E-13 | 0.1660 | -0.0859 | 0.2240 | 7.01E-01 | 0.1157 | 0.0306 | 0.0249 | 2.18E-01 | 0.1158 |
| rs150527451 | 11 | 68817897 | A | G | 0.0262 | 0.0035 | 4.00E-14 | 0.1064 | 0.0277 | 0.1715 | 8.72E-01 | 0.2342 | 0.0444 | 0.0189 | 1.85E-02 | 0.2346 |
| rs151337382 | 20 | 34204925 | A | T | 0.0575 | 0.0093 | 5.20E-10 | 0.0136 | 0.0334 | 1.0367 | 9.74E-01 | 0.0046 | -0.0395 | 0.1192 | 7.41E-01 | 0.0046 |
| rs1548714 | 17 | 26280204 | C | A | 0.0162 | 0.0027 | 3.00E-09 | 0.8117 | 0.1183 | 0.1838 | 5.20E-01 | 0.8126 | 0.0011 | 0.0204 | 9.56E-01 | 0.8126 |
| rs16891982 | 5 | 33951693 | G | C | 0.2292 | 0.0062 | 1.00E-200 | 0.9709 | 1.0331 | 0.5523 | 6.14E-02 | 0.9821 | 0.3270 | 0.0642 | 3.57E-07 | 0.9824 |
| rs17232484 | 16 | 89861650 | A | G | 0.0610 | 0.0091 | 1.80E-11 | 0.0142 | -0.2844 | 0.5549 | 6.08E-01 | 0.0197 | -0.0404 | 0.0616 | 5.12E-01 | 0.0197 |
| rs1805007 | 16 | 89986117 | T | C | 0.1964 | 0.0035 | 1.00E-200 | 0.1009 | 0.7892 | 0.2954 | 7.54E-03 | 0.0659 | 0.4357 | 0.0324 | 2.57E-41 | 0.0674 |
| rs1805008 | 16 | 89986144 | T | C | 0.1195 | 0.0038 | 1.00E-200 | 0.0866 | 0.2050 | 0.2826 | 4.68E-01 | 0.0674 | 0.3355 | 0.0315 | 1.85E-26 | 0.0686 |
| rs1989483 | 7 | 16942661 | G | A | 0.0125 | 0.0022 | 1.20E-08 | 0.3891 | 0.2013 | 0.1483 | 1.75E-01 | 0.3680 | 0.0306 | 0.0165 | 6.29E-02 | 0.3683 |
| rs2299098 | 7 | 24756377 | C | G | 0.0148 | 0.0027 | 3.60E-08 | 0.1962 | -0.0357 | 0.1963 | 8.56E-01 | 0.1557 | 0.0161 | 0.0219 | 4.62E-01 | 0.1559 |
| rs251468 | 5 | 149194485 | T | C | -0.0282 | 0.0025 | 4.40E-30 | 0.2484 | -0.0386 | 0.1511 | 7.99E-01 | 0.3446 | -0.0525 | 0.0167 | 1.70E-03 | 0.3439 |
| rs2737217 | 8 | 116630311 | G | A | -0.0255 | 0.0022 | 4.10E-32 | 0.5633 | 0.0226 | 0.1526 | 8.82E-01 | 0.6672 | -0.0437 | 0.0169 | 9.88E-03 | 0.6666 |
| rs3213737 | 12 | 96379806 | A | G | -0.0319 | 0.0022 | 2.70E-49 | 0.5760 | -0.2156 | 0.1501 | 1.51E-01 | 0.6553 | -0.0142 | 0.0167 | 3.94E-01 | 0.6551 |
| rs35563099 | 10 | 119572403 | T | C | -0.0310 | 0.0029 | 1.80E-26 | 0.1640 | -0.0505 | 0.1861 | 7.86E-01 | 0.1861 | -0.0689 | 0.0204 | 7.39E-04 | 0.1855 |
| rs3759579 | 14 | 103851272 | G | A | 0.0124 | 0.0022 | 8.10E-09 | 0.5888 | 0.0410 | 0.1477 | 7.82E-01 | 0.6204 | 0.0175 | 0.0164 | 2.86E-01 | 0.6206 |
| rs41563 | 7 | 104852654 | A | G | 0.0135 | 0.0022 | 1.40E-09 | 0.3498 | -0.2247 | 0.1553 | 1.48E-01 | 0.3085 | -0.0087 | 0.0172 | 6.13E-01 | 0.3083 |
| rs4240559 | 6 | 98437775 | C | T | -0.0171 | 0.0021 | 1.40E-15 | 0.5579 | -0.1835 | 0.1499 | 2.21E-01 | 0.6516 | -0.0066 | 0.0166 | 6.92E-01 | 0.6515 |
| rs4272574 | 1 | 73661205 | T | C | 0.0152 | 0.0021 | 8.40E-13 | 0.4783 | -0.0946 | 0.1433 | 5.09E-01 | 0.5100 | 0.0301 | 0.0159 | 5.81E-02 | 0.5103 |
| rs4335021 | 6 | 32386619 | C | T | -0.0118 | 0.0022 | 4.90E-08 | 0.5997 | -0.3714 | 0.1757 | 3.46E-02 | 0.6435 | -0.0388 | 0.0195 | 4.64E-02 | 0.6428 |
| rs4438032 | 11 | 88786692 | G | C | 0.0232 | 0.0036 | 2.00E-10 | 0.9062 | -0.4184 | 0.2755 | 1.29E-01 | 0.9272 | 0.0098 | 0.0307 | 7.49E-01 | 0.9273 |
| rs4578351 | 11 | 16587580 | C | T | -0.0205 | 0.0026 | 1.70E-15 | 0.2220 | 0.1068 | 0.1664 | 5.21E-01 | 0.2504 | -0.0232 | 0.0184 | 2.07E-01 | 0.2501 |
| rs4670813 | 2 | 38317710 | A | G | -0.0127 | 0.0021 | 3.50E-09 | 0.4708 | -0.3045 | 0.1436 | 3.40E-02 | 0.5244 | -0.0492 | 0.0159 | 1.95E-03 | 0.5239 |
| rs4840542 | 8 | 10944809 | T | G | 0.0185 | 0.0021 | 4.00E-18 | 0.5058 | -0.2013 | 0.2742 | 4.63E-01 | 0.4248 | 0.0346 | 0.0307 | 2.59E-01 | 0.4252 |
| rs511515 | 6 | 33541507 | G | A | -0.0204 | 0.0023 | 1.60E-18 | 0.7009 | -0.1256 | 0.1736 | 4.69E-01 | 0.7820 | -0.0131 | 0.0192 | 4.96E-01 | 0.7818 |
| rs537894 | 3 | 138348595 | A | G | 0.0127 | 0.0022 | 5.00E-09 | 0.5490 | 0.2123 | 0.1545 | 1.69E-01 | 0.6725 | 0.0229 | 0.0171 | 1.82E-01 | 0.6727 |
| rs57994353 | 9 | 139356987 | C | T | 0.0127 | 0.0023 | 4.30E-08 | 0.2991 | -0.2158 | 0.1640 | 1.88E-01 | 0.2557 | 0.0241 | 0.0182 | 1.85E-01 | 0.2559 |
| rs6007506 | 22 | 45622014 | T | C | -0.0248 | 0.0023 | 4.10E-28 | 0.3374 | 0.0266 | 0.1488 | 8.58E-01 | 0.3561 | -0.0248 | 0.0165 | 1.34E-01 | 0.3558 |
| rs6059655 | 20 | 32665748 | G | A | -0.1273 | 0.0036 | 1.00E-200 | 0.8980 | -0.3235 | 0.4754 | 4.96E-01 | 0.9778 | -0.3415 | 0.0531 | 1.24E-10 | 0.9774 |
| rs61816766 | 1 | 152319572 | C | T | 0.0366 | 0.0062 | 3.40E-09 | 0.0323 | -0.4285 | 0.7861 | 5.86E-01 | 0.0079 | 0.0884 | 0.0893 | 3.22E-01 | 0.0079 |
| rs61981034 | 14 | 97377089 | A | G | 0.0133 | 0.0024 | 4.60E-08 | 0.2580 | -0.0415 | 0.1519 | 7.85E-01 | 0.3372 | 0.0056 | 0.0168 | 7.40E-01 | 0.3373 |
| rs6689641 | 1 | 110720400 | G | A | 0.0149 | 0.0021 | 2.60E-12 | 0.5426 | 0.2424 | 0.1439 | 9.21E-02 | 0.5581 | 0.0297 | 0.0160 | 6.27E-02 | 0.5583 |
| rs6882046 | 5 | 87968864 | G | A | 0.0144 | 0.0024 | 3.10E-09 | 0.2679 | -0.0421 | 0.1854 | 8.20E-01 | 0.1797 | 0.0275 | 0.0207 | 1.84E-01 | 0.1801 |
| rs699780 | 1 | 120455441 | G | A | -0.0184 | 0.0032 | 7.00E-09 | 0.1278 | 0.0695 | 0.1908 | 7.16E-01 | 0.1681 | 0.0126 | 0.0212 | 5.51E-01 | 0.1682 |
| rs72821630 | 2 | 63696212 | T | C | -0.0150 | 0.0025 | 9.00E-10 | 0.2514 | -0.0463 | 0.1497 | 7.57E-01 | 0.3631 | 0.0069 | 0.0165 | 6.75E-01 | 0.3632 |
| rs75300484 | 16 | 89489061 | T | C | -0.0342 | 0.0058 | 3.70E-09 | 0.0347 | 0.0133 | 0.4592 | 9.77E-01 | 0.0233 | -0.1372 | 0.0527 | 9.26E-03 | 0.0232 |
| rs75908072 | 6 | 290438 | C | T | -0.0318 | 0.0057 | 2.80E-08 | 0.0366 | 0.6854 | 0.5098 | 1.79E-01 | 0.0226 | 0.0143 | 0.0564 | 8.00E-01 | 0.0226 |
| rs7768317 | 6 | 41922220 | T | C | -0.0170 | 0.0025 | 4.90E-12 | 0.2489 | -0.0019 | 0.1630 | 9.91E-01 | 0.2629 | -0.0293 | 0.0180 | 1.04E-01 | 0.2626 |
| rs784235 | 18 | 53423144 | G | A | -0.0164 | 0.0028 | 3.00E-09 | 0.8191 | -0.1410 | 0.2032 | 4.88E-01 | 0.8553 | -0.0155 | 0.0225 | 4.92E-01 | 0.8551 |
| rs78444298 | 1 | 184672098 | A | G | -0.0467 | 0.0077 | 1.40E-09 | 0.0197 | 0.7884 | 0.5512 | 1.53E-01 | 0.0173 | 0.0223 | 0.0607 | 7.13E-01 | 0.0173 |
| rs849138 | 7 | 28177338 | A | G | 0.0126 | 0.0021 | 3.80E-09 | 0.5070 | 0.0482 | 0.1434 | 7.37E-01 | 0.4896 | 0.0431 | 0.0159 | 6.70E-03 | 0.4901 |
| rs9328259 | 6 | 508972 | A | C | 0.0267 | 0.0024 | 1.60E-29 | 0.7182 | 0.1109 | 0.1594 | 4.87E-01 | 0.7207 | -0.0007 | 0.0176 | 9.68E-01 | 0.7205 |
| rs964127 | 12 | 96290733 | T | A | 0.0123 | 0.0023 | 4.90E-08 | 0.3328 | -0.0038 | 0.1435 | 9.79E-01 | 0.4645 | -0.0098 | 0.0159 | 5.36E-01 | 0.4643 |
| rs9821675 | 3 | 49902544 | G | A | -0.0120 | 0.0021 | 1.50E-08 | 0.5053 | -0.1464 | 0.1463 | 3.17E-01 | 0.3953 | -0.0374 | 0.0162 | 2.13E-02 | 0.3947 |
| rs9832130 | 3 | 189194752 | A | G | -0.0128 | 0.0022 | 3.30E-09 | 0.5807 | -0.0373 | 0.1443 | 7.96E-01 | 0.5602 | 0.0421 | 0.0160 | 8.49E-03 | 0.5607 |
| rs9835772 | 3 | 85766025 | T | A | 0.0177 | 0.0025 | 8.60E-13 | 0.2437 | 0.0243 | 0.1697 | 8.86E-01 | 0.2291 | 0.0030 | 0.0189 | 8.74E-01 | 0.2291 |
| rs9858244 | 3 | 85787399 | A | G | 0.0189 | 0.0026 | 3.00E-13 | 0.2142 | -0.0162 | 0.1835 | 9.30E-01 | 0.1847 | -0.0123 | 0.0204 | 5.47E-01 | 0.1846 |
| rs9867857 | 3 | 156491160 | T | C | 0.0136 | 0.0021 | 1.80E-10 | 0.4892 | 0.1995 | 0.1449 | 1.69E-01 | 0.4292 | 0.0344 | 0.0160 | 3.17E-02 | 0.4297 |
| rs9904468 | 17 | 17573187 | G | C | 0.0137 | 0.0021 | 1.10E-10 | 0.5335 | -0.2248 | 0.1434 | 1.17E-01 | 0.5504 | -0.0136 | 0.0159 | 3.92E-01 | 0.5503 |

SNP: Single-nucleotide polymorphisms; Chr: Chromosome; EA: Effect allele; OA: Other allele; EAF: Effect allele frequency.

**Supplementary Table 7.** Information of identified SNPs in exposure (Childhood sunburn) and outcomes (overall Melanoma in situ and Melanoma in situ of face, controls excluding all cancers).

| SNP | | | | | Exposure (Childhood sunburn) | | | | Outcome (Malignant melanoma of skin, controls excluding all cancers) | | | | Outcome (Non-melanoma skin cancer, controls excluding all cancers) | | | |
| --- | --- | --- | --- | --- | --- | --- | --- | --- | --- | --- | --- | --- | --- | --- | --- | --- |
| RS ID | Chr | Position | EA | OA | Beta | Se | P-value | EAF | Beta | Se | P-value | EAF | Beta | Se | P-value | EAF |
| rs10168349 | 2 | 46360907 | C | G | 0.0173 | 0.0023 | 1.40E-14 | 0.3357 | -0.0002 | 0.1596 | 9.99E-01 | 0.2822 | 0.0219 | 0.0177 | 2.15E-01 | 0.2824 |
| rs10202908 | 2 | 169378292 | T | C | -0.0126 | 0.0023 | 2.90E-08 | 0.6749 | 0.2495 | 0.1565 | 1.11E-01 | 0.6990 | 0.0127 | 0.0173 | 4.65E-01 | 0.6991 |
| rs10220751 | 15 | 47923520 | G | T | -0.0137 | 0.0022 | 2.50E-10 | 0.4048 | 0.0376 | 0.1487 | 8.01E-01 | 0.3600 | -0.0163 | 0.0165 | 3.23E-01 | 0.3598 |
| rs10788627 | 10 | 82203069 | C | T | 0.0120 | 0.0021 | 1.60E-08 | 0.4745 | 0.0019 | 0.1436 | 9.89E-01 | 0.5468 | 0.0192 | 0.0159 | 2.29E-01 | 0.5471 |
| rs10810636 | 9 | 16799109 | G | A | 0.0275 | 0.0025 | 2.80E-28 | 0.7603 | 0.0410 | 0.1628 | 8.01E-01 | 0.7365 | 0.0734 | 0.0181 | 4.80E-05 | 0.7373 |
| rs10873552 | 14 | 105433129 | G | A | 0.0124 | 0.0022 | 3.70E-08 | 0.6577 | -0.0740 | 0.1449 | 6.10E-01 | 0.5739 | -0.0121 | 0.0161 | 4.50E-01 | 0.5737 |
| rs10896139 | 11 | 66650060 | T | C | -0.0136 | 0.0024 | 1.40E-08 | 0.2709 | -0.1792 | 0.1594 | 2.61E-01 | 0.2780 | 0.0193 | 0.0177 | 2.76E-01 | 0.2783 |
| rs11070811 | 15 | 31394082 | T | C | -0.0176 | 0.0027 | 1.50E-10 | 0.1832 | -0.1218 | 0.2092 | 5.61E-01 | 0.1333 | -0.0527 | 0.0233 | 2.36E-02 | 0.1331 |
| rs11104733 | 12 | 88490119 | T | C | 0.0492 | 0.0089 | 2.80E-08 | 0.0147 | -0.3342 | 0.4822 | 4.88E-01 | 0.0235 | 0.0787 | 0.0526 | 1.35E-01 | 0.0236 |
| rs111391498 | 4 | 1341553 | G | A | -0.0474 | 0.0050 | 1.70E-21 | 0.0479 | -0.3016 | 0.4299 | 4.83E-01 | 0.0286 | 0.0516 | 0.0472 | 2.74E-01 | 0.0287 |
| rs111650620 | 20 | 34591725 | A | G | 0.6618 | 0.0601 | 3.20E-28 | 0.0022 | -0.2473 | 0.4434 | 5.77E-01 | 0.0273 | 0.0131 | 0.0483 | 7.87E-01 | 0.0273 |
| rs112089506 | 16 | 90149171 | T | C | -0.0581 | 0.0040 | 1.40E-47 | 0.0812 | -0.1878 | 0.2470 | 4.47E-01 | 0.0939 | -0.1320 | 0.0275 | 1.63E-06 | 0.0934 |
| rs11242899 | 6 | 460302 | A | G | -0.0245 | 0.0024 | 3.20E-24 | 0.2663 | 0.0553 | 0.1628 | 7.34E-01 | 0.2624 | 0.0211 | 0.0181 | 2.43E-01 | 0.2626 |
| rs1126809 | 11 | 89017961 | A | G | 0.0825 | 0.0023 | 1.00E-200 | 0.3028 | -0.0919 | 0.1868 | 6.23E-01 | 0.1765 | 0.1648 | 0.0208 | 2.37E-15 | 0.1779 |
| rs116125333 | 5 | 33930012 | G | T | -0.0397 | 0.0072 | 3.40E-08 | 0.0232 | -0.0195 | 0.2633 | 9.41E-01 | 0.0842 | -0.0268 | 0.0289 | 3.54E-01 | 0.0841 |
| rs11648436 | 16 | 14008674 | T | C | -0.0273 | 0.0022 | 9.90E-35 | 0.3564 | -0.0698 | 0.1556 | 6.54E-01 | 0.3077 | -0.0010 | 0.0172 | 9.55E-01 | 0.3077 |
| rs117132860 | 7 | 17134708 | A | G | 0.0593 | 0.0067 | 1.20E-18 | 0.0256 | 2.0444 | 1.1811 | 8.35E-02 | 0.0039 | 0.4590 | 0.1287 | 3.63E-04 | 0.0040 |
| rs11739906 | 5 | 59019359 | C | A | 0.0130 | 0.0023 | 8.40E-09 | 0.3275 | -0.0004 | 0.1669 | 9.98E-01 | 0.2401 | 0.0041 | 0.0186 | 8.24E-01 | 0.2402 |
| rs117462393 | 16 | 14277804 | T | C | 0.0815 | 0.0096 | 1.50E-17 | 0.0144 | -0.0191 | 0.2933 | 9.48E-01 | 0.0651 | -0.0244 | 0.0327 | 4.56E-01 | 0.0650 |
| rs12203592 | 6 | 396321 | T | C | 0.1531 | 0.0025 | 1.00E-200 | 0.2191 | 0.1575 | 0.4250 | 7.11E-01 | 0.0301 | 0.3746 | 0.0477 | 4.29E-15 | 0.0306 |
| rs1233578 | 6 | 28712247 | G | A | -0.0206 | 0.0028 | 1.50E-13 | 0.1764 | -0.7421 | 0.3115 | 1.72E-02 | 0.0604 | 0.0496 | 0.0341 | 1.45E-01 | 0.0606 |
| rs12350739 | 9 | 16885017 | A | G | 0.0355 | 0.0022 | 5.80E-59 | 0.6068 | -0.1895 | 0.1436 | 1.87E-01 | 0.5454 | 0.0769 | 0.0159 | 1.40E-06 | 0.5465 |
| rs1260326 | 2 | 27730940 | C | T | -0.0125 | 0.0022 | 8.10E-09 | 0.6040 | 0.0794 | 0.1500 | 5.96E-01 | 0.6491 | 0.0072 | 0.0166 | 6.65E-01 | 0.6492 |
| rs1267038 | 2 | 162099153 | C | A | -0.0179 | 0.0030 | 2.20E-09 | 0.8449 | -0.0314 | 0.1860 | 8.66E-01 | 0.8187 | 0.0361 | 0.0207 | 8.10E-02 | 0.8190 |
| rs1278766 | 13 | 113534382 | C | T | 0.0191 | 0.0021 | 3.90E-19 | 0.5457 | -0.1373 | 0.1434 | 3.38E-01 | 0.4873 | 0.0511 | 0.0159 | 1.30E-03 | 0.4880 |
| rs12913832 | 15 | 28365618 | G | A | 0.0605 | 0.0025 | 1.90E-126 | 0.7753 | -0.0358 | 0.2358 | 8.79E-01 | 0.8944 | -0.1461 | 0.0260 | 1.90E-08 | 0.8937 |
| rs1308048 | 1 | 66888542 | C | T | -0.0204 | 0.0022 | 5.30E-21 | 0.4195 | 0.1589 | 0.1447 | 2.72E-01 | 0.4299 | -0.0553 | 0.0160 | 5.60E-04 | 0.4291 |
| rs1326798 | 9 | 12722227 | G | C | 0.0121 | 0.0022 | 3.60E-08 | 0.6206 | 0.5467 | 0.1502 | 2.73E-04 | 0.6349 | 0.0714 | 0.0165 | 1.51E-05 | 0.6356 |
| rs13332673 | 16 | 89940386 | T | G | -0.1021 | 0.0105 | 3.30E-22 | 0.0102 | -1.0617 | 0.8631 | 2.19E-01 | 0.0073 | -0.0103 | 0.0951 | 9.14E-01 | 0.0073 |
| rs139414522 | 16 | 89821155 | C | T | -0.0784 | 0.0091 | 5.40E-18 | 0.0153 | 0.6775 | 0.7658 | 3.76E-01 | 0.0093 | 0.1176 | 0.0830 | 1.57E-01 | 0.0093 |
| rs141817469 | 16 | 89927151 | T | C | -0.0932 | 0.0058 | 1.00E-57 | 0.0351 | 0.1287 | 0.4109 | 7.54E-01 | 0.0319 | -0.2008 | 0.0462 | 1.36E-05 | 0.0316 |
| rs142314514 | 16 | 90130996 | G | A | -0.0816 | 0.0070 | 3.60E-31 | 0.0285 | 0.0646 | 0.4454 | 8.85E-01 | 0.0281 | -0.1830 | 0.0506 | 2.99E-04 | 0.0280 |
| rs1437635 | 11 | 16358722 | A | C | 0.0207 | 0.0029 | 5.20E-13 | 0.1660 | -0.0859 | 0.2240 | 7.01E-01 | 0.1157 | 0.0306 | 0.0249 | 2.18E-01 | 0.1158 |
| rs150527451 | 11 | 68817897 | A | G | 0.0262 | 0.0035 | 4.00E-14 | 0.1064 | 0.0277 | 0.1715 | 8.72E-01 | 0.2342 | 0.0444 | 0.0189 | 1.85E-02 | 0.2346 |
| rs151337382 | 20 | 34204925 | A | T | 0.0575 | 0.0093 | 5.20E-10 | 0.0136 | 0.0334 | 1.0367 | 9.74E-01 | 0.0046 | -0.0395 | 0.1192 | 7.41E-01 | 0.0046 |
| rs1548714 | 17 | 26280204 | C | A | 0.0162 | 0.0027 | 3.00E-09 | 0.8117 | 0.1183 | 0.1838 | 5.20E-01 | 0.8126 | 0.0011 | 0.0204 | 9.56E-01 | 0.8126 |
| rs16891982 | 5 | 33951693 | G | C | 0.2292 | 0.0062 | 1.00E-200 | 0.9709 | 1.0331 | 0.5523 | 6.14E-02 | 0.9821 | 0.3270 | 0.0642 | 3.57E-07 | 0.9824 |
| rs17232484 | 16 | 89861650 | A | G | 0.0610 | 0.0091 | 1.80E-11 | 0.0142 | -0.2844 | 0.5549 | 6.08E-01 | 0.0197 | -0.0404 | 0.0616 | 5.12E-01 | 0.0197 |
| rs1805007 | 16 | 89986117 | T | C | 0.1964 | 0.0035 | 1.00E-200 | 0.1009 | 0.7892 | 0.2954 | 7.54E-03 | 0.0659 | 0.4357 | 0.0324 | 2.57E-41 | 0.0674 |
| rs1805008 | 16 | 89986144 | T | C | 0.1195 | 0.0038 | 1.00E-200 | 0.0866 | 0.2050 | 0.2826 | 4.68E-01 | 0.0674 | 0.3355 | 0.0315 | 1.85E-26 | 0.0686 |
| rs1989483 | 7 | 16942661 | G | A | 0.0125 | 0.0022 | 1.20E-08 | 0.3891 | 0.2013 | 0.1483 | 1.75E-01 | 0.3680 | 0.0306 | 0.0165 | 6.29E-02 | 0.3683 |
| rs2299098 | 7 | 24756377 | C | G | 0.0148 | 0.0027 | 3.60E-08 | 0.1962 | -0.0357 | 0.1963 | 8.56E-01 | 0.1557 | 0.0161 | 0.0219 | 4.62E-01 | 0.1559 |
| rs251468 | 5 | 149194485 | T | C | -0.0282 | 0.0025 | 4.40E-30 | 0.2484 | -0.0386 | 0.1511 | 7.99E-01 | 0.3446 | -0.0525 | 0.0167 | 1.70E-03 | 0.3439 |
| rs2737217 | 8 | 116630311 | G | A | -0.0255 | 0.0022 | 4.10E-32 | 0.5633 | 0.0226 | 0.1526 | 8.82E-01 | 0.6672 | -0.0437 | 0.0169 | 9.88E-03 | 0.6666 |
| rs3213737 | 12 | 96379806 | A | G | -0.0319 | 0.0022 | 2.70E-49 | 0.5760 | -0.2156 | 0.1501 | 1.51E-01 | 0.6553 | -0.0142 | 0.0167 | 3.94E-01 | 0.6551 |
| rs35563099 | 10 | 119572403 | T | C | -0.0310 | 0.0029 | 1.80E-26 | 0.1640 | -0.0505 | 0.1861 | 7.86E-01 | 0.1861 | -0.0689 | 0.0204 | 7.39E-04 | 0.1855 |
| rs3759579 | 14 | 103851272 | G | A | 0.0124 | 0.0022 | 8.10E-09 | 0.5888 | 0.0410 | 0.1477 | 7.82E-01 | 0.6204 | 0.0175 | 0.0164 | 2.86E-01 | 0.6206 |
| rs41563 | 7 | 104852654 | A | G | 0.0135 | 0.0022 | 1.40E-09 | 0.3498 | -0.2247 | 0.1553 | 1.48E-01 | 0.3085 | -0.0087 | 0.0172 | 6.13E-01 | 0.3083 |
| rs4240559 | 6 | 98437775 | C | T | -0.0171 | 0.0021 | 1.40E-15 | 0.5579 | -0.1835 | 0.1499 | 2.21E-01 | 0.6516 | -0.0066 | 0.0166 | 6.92E-01 | 0.6515 |
| rs4272574 | 1 | 73661205 | T | C | 0.0152 | 0.0021 | 8.40E-13 | 0.4783 | -0.0946 | 0.1433 | 5.09E-01 | 0.5100 | 0.0301 | 0.0159 | 5.81E-02 | 0.5103 |
| rs4335021 | 6 | 32386619 | C | T | -0.0118 | 0.0022 | 4.90E-08 | 0.5997 | -0.3714 | 0.1757 | 3.46E-02 | 0.6435 | -0.0388 | 0.0195 | 4.64E-02 | 0.6428 |
| rs4438032 | 11 | 88786692 | G | C | 0.0232 | 0.0036 | 2.00E-10 | 0.9062 | -0.4184 | 0.2755 | 1.29E-01 | 0.9272 | 0.0098 | 0.0307 | 7.49E-01 | 0.9273 |
| rs4578351 | 11 | 16587580 | C | T | -0.0205 | 0.0026 | 1.70E-15 | 0.2220 | 0.1068 | 0.1664 | 5.21E-01 | 0.2504 | -0.0232 | 0.0184 | 2.07E-01 | 0.2501 |
| rs4670813 | 2 | 38317710 | A | G | -0.0127 | 0.0021 | 3.50E-09 | 0.4708 | -0.3045 | 0.1436 | 3.40E-02 | 0.5244 | -0.0492 | 0.0159 | 1.95E-03 | 0.5239 |
| rs4840542 | 8 | 10944809 | T | G | 0.0185 | 0.0021 | 4.00E-18 | 0.5058 | -0.2013 | 0.2742 | 4.63E-01 | 0.4248 | 0.0346 | 0.0307 | 2.59E-01 | 0.4252 |
| rs511515 | 6 | 33541507 | G | A | -0.0204 | 0.0023 | 1.60E-18 | 0.7009 | -0.1256 | 0.1736 | 4.69E-01 | 0.7820 | -0.0131 | 0.0192 | 4.96E-01 | 0.7818 |
| rs537894 | 3 | 138348595 | A | G | 0.0127 | 0.0022 | 5.00E-09 | 0.5490 | 0.2123 | 0.1545 | 1.69E-01 | 0.6725 | 0.0229 | 0.0171 | 1.82E-01 | 0.6727 |
| rs57994353 | 9 | 139356987 | C | T | 0.0127 | 0.0023 | 4.30E-08 | 0.2991 | -0.2158 | 0.1640 | 1.88E-01 | 0.2557 | 0.0241 | 0.0182 | 1.85E-01 | 0.2559 |
| rs6007506 | 22 | 45622014 | T | C | -0.0248 | 0.0023 | 4.10E-28 | 0.3374 | 0.0266 | 0.1488 | 8.58E-01 | 0.3561 | -0.0248 | 0.0165 | 1.34E-01 | 0.3558 |
| rs6059655 | 20 | 32665748 | G | A | -0.1273 | 0.0036 | 1.00E-200 | 0.8980 | -0.3235 | 0.4754 | 4.96E-01 | 0.9778 | -0.3415 | 0.0531 | 1.24E-10 | 0.9774 |
| rs61816766 | 1 | 152319572 | C | T | 0.0366 | 0.0062 | 3.40E-09 | 0.0323 | -0.4285 | 0.7861 | 5.86E-01 | 0.0079 | 0.0884 | 0.0893 | 3.22E-01 | 0.0079 |
| rs61981034 | 14 | 97377089 | A | G | 0.0133 | 0.0024 | 4.60E-08 | 0.2580 | -0.0415 | 0.1519 | 7.85E-01 | 0.3372 | 0.0056 | 0.0168 | 7.40E-01 | 0.3373 |
| rs6689641 | 1 | 110720400 | G | A | 0.0149 | 0.0021 | 2.60E-12 | 0.5426 | 0.2424 | 0.1439 | 9.21E-02 | 0.5581 | 0.0297 | 0.0160 | 6.27E-02 | 0.5583 |
| rs6882046 | 5 | 87968864 | G | A | 0.0144 | 0.0024 | 3.10E-09 | 0.2679 | -0.0421 | 0.1854 | 8.20E-01 | 0.1797 | 0.0275 | 0.0207 | 1.84E-01 | 0.1801 |
| rs699780 | 1 | 120455441 | G | A | -0.0184 | 0.0032 | 7.00E-09 | 0.1278 | 0.0695 | 0.1908 | 7.16E-01 | 0.1681 | 0.0126 | 0.0212 | 5.51E-01 | 0.1682 |
| rs72821630 | 2 | 63696212 | T | C | -0.0150 | 0.0025 | 9.00E-10 | 0.2514 | -0.0463 | 0.1497 | 7.57E-01 | 0.3631 | 0.0069 | 0.0165 | 6.75E-01 | 0.3632 |
| rs75300484 | 16 | 89489061 | T | C | -0.0342 | 0.0058 | 3.70E-09 | 0.0347 | 0.0133 | 0.4592 | 9.77E-01 | 0.0233 | -0.1372 | 0.0527 | 9.26E-03 | 0.0232 |
| rs75908072 | 6 | 290438 | C | T | -0.0318 | 0.0057 | 2.80E-08 | 0.0366 | 0.6854 | 0.5098 | 1.79E-01 | 0.0226 | 0.0143 | 0.0564 | 8.00E-01 | 0.0226 |
| rs7768317 | 6 | 41922220 | T | C | -0.0170 | 0.0025 | 4.90E-12 | 0.2489 | -0.0019 | 0.1630 | 9.91E-01 | 0.2629 | -0.0293 | 0.0180 | 1.04E-01 | 0.2626 |
| rs784235 | 18 | 53423144 | G | A | -0.0164 | 0.0028 | 3.00E-09 | 0.8191 | -0.1410 | 0.2032 | 4.88E-01 | 0.8553 | -0.0155 | 0.0225 | 4.92E-01 | 0.8551 |
| rs78444298 | 1 | 184672098 | A | G | -0.0467 | 0.0077 | 1.40E-09 | 0.0197 | 0.7884 | 0.5512 | 1.53E-01 | 0.0173 | 0.0223 | 0.0607 | 7.13E-01 | 0.0173 |
| rs849138 | 7 | 28177338 | A | G | 0.0126 | 0.0021 | 3.80E-09 | 0.5070 | 0.0482 | 0.1434 | 7.37E-01 | 0.4896 | 0.0431 | 0.0159 | 6.70E-03 | 0.4901 |
| rs9328259 | 6 | 508972 | A | C | 0.0267 | 0.0024 | 1.60E-29 | 0.7182 | 0.1109 | 0.1594 | 4.87E-01 | 0.7207 | -0.0007 | 0.0176 | 9.68E-01 | 0.7205 |
| rs964127 | 12 | 96290733 | T | A | 0.0123 | 0.0023 | 4.90E-08 | 0.3328 | -0.0038 | 0.1435 | 9.79E-01 | 0.4645 | -0.0098 | 0.0159 | 5.36E-01 | 0.4643 |
| rs9821675 | 3 | 49902544 | G | A | -0.0120 | 0.0021 | 1.50E-08 | 0.5053 | -0.1464 | 0.1463 | 3.17E-01 | 0.3953 | -0.0374 | 0.0162 | 2.13E-02 | 0.3947 |
| rs9832130 | 3 | 189194752 | A | G | -0.0128 | 0.0022 | 3.30E-09 | 0.5807 | -0.0373 | 0.1443 | 7.96E-01 | 0.5602 | 0.0421 | 0.0160 | 8.49E-03 | 0.5607 |
| rs9835772 | 3 | 85766025 | T | A | 0.0177 | 0.0025 | 8.60E-13 | 0.2437 | 0.0243 | 0.1697 | 8.86E-01 | 0.2291 | 0.0030 | 0.0189 | 8.74E-01 | 0.2291 |
| rs9858244 | 3 | 85787399 | A | G | 0.0189 | 0.0026 | 3.00E-13 | 0.2142 | -0.0162 | 0.1835 | 9.30E-01 | 0.1847 | -0.0123 | 0.0204 | 5.47E-01 | 0.1846 |
| rs9867857 | 3 | 156491160 | T | C | 0.0136 | 0.0021 | 1.80E-10 | 0.4892 | 0.1995 | 0.1449 | 1.69E-01 | 0.4292 | 0.0344 | 0.0160 | 3.17E-02 | 0.4297 |
| rs9904468 | 17 | 17573187 | G | C | 0.0137 | 0.0021 | 1.10E-10 | 0.5335 | -0.2248 | 0.1434 | 1.17E-01 | 0.5504 | -0.0136 | 0.0159 | 3.92E-01 | 0.5503 |

SNP: Single-nucleotide polymorphisms; Chr: Chromosome; EA: Effect allele; OA: Other allele; EAF: Effect allele frequency.

**Supplementary Table 8.** Information of identified SNPs in exposure (Childhood sunburn) and outcomes (Malignant melanoma of skin and non-melanoma skin cancer, controls excluding all cancers).

| SNP | | | | | Exposure (Childhood sunburn) | | | | Outcome ( Melanoma in situ, controls excluding all cancers) | | | | Outcome (Melanoma in situ of face, controls excluding all cancers) | | | |
| --- | --- | --- | --- | --- | --- | --- | --- | --- | --- | --- | --- | --- | --- | --- | --- | --- |
| RS ID | Chr | Position | EA | OA | Beta | Se | P-value | EAF | Beta | Se | P-value | EAF | Beta | Se | P-value | EAF |
| rs10168349 | 2 | 46360907 | C | G | 0.0173 | 0.0023 | 1.40E-14 | 0.3357 | 0.1408 | 0.0806 | 8.05E-02 | 0.2824 | 0.1949 | 0.1464 | 1.83E-01 | 0.2824 |
| rs10202908 | 2 | 169378292 | T | C | -0.0126 | 0.0023 | 2.90E-08 | 0.6749 | -0.0238 | 0.0788 | 7.62E-01 | 0.6988 | 0.0782 | 0.1429 | 5.84E-01 | 0.6988 |
| rs10220751 | 15 | 47923520 | G | T | -0.0137 | 0.0022 | 2.50E-10 | 0.4048 | -0.0259 | 0.0750 | 7.29E-01 | 0.3601 | 0.0019 | 0.1362 | 9.89E-01 | 0.3601 |
| rs10788627 | 10 | 82203069 | C | T | 0.0120 | 0.0021 | 1.60E-08 | 0.4745 | -0.0626 | 0.0727 | 3.89E-01 | 0.5466 | -0.0768 | 0.1322 | 5.61E-01 | 0.5466 |
| rs10810636 | 9 | 16799109 | G | A | 0.0275 | 0.0025 | 2.80E-28 | 0.7603 | 0.0475 | 0.0822 | 5.63E-01 | 0.7367 | 0.1473 | 0.1488 | 3.22E-01 | 0.7367 |
| rs10873552 | 14 | 105433129 | G | A | 0.0124 | 0.0022 | 3.70E-08 | 0.6577 | 0.0188 | 0.0733 | 7.97E-01 | 0.5738 | -0.0766 | 0.1330 | 5.65E-01 | 0.5738 |
| rs10896139 | 11 | 66650060 | T | C | -0.0136 | 0.0024 | 1.40E-08 | 0.2709 | 0.0009 | 0.0810 | 9.92E-01 | 0.2783 | 0.0133 | 0.1468 | 9.28E-01 | 0.2782 |
| rs11070811 | 15 | 31394082 | T | C | -0.0176 | 0.0027 | 1.50E-10 | 0.1832 | -0.1498 | 0.1053 | 1.55E-01 | 0.1332 | -0.2509 | 0.1916 | 1.90E-01 | 0.1332 |
| rs11104733 | 12 | 88490119 | T | C | 0.0492 | 0.0089 | 2.80E-08 | 0.0147 | 0.6286 | 0.2418 | 9.33E-03 | 0.0236 | 1.0306 | 0.4341 | 1.76E-02 | 0.0236 |
| rs111391498 | 4 | 1341553 | G | A | -0.0474 | 0.0050 | 1.70E-21 | 0.0479 | -0.0363 | 0.2171 | 8.67E-01 | 0.0285 | 0.3253 | 0.3923 | 4.07E-01 | 0.0285 |
| rs111650620 | 20 | 34591725 | A | G | 0.6618 | 0.0601 | 3.20E-28 | 0.0022 | -0.0058 | 0.2245 | 9.79E-01 | 0.0273 | 0.1209 | 0.4086 | 7.67E-01 | 0.0272 |
| rs112089506 | 16 | 90149171 | T | C | -0.0581 | 0.0040 | 1.40E-47 | 0.0812 | -0.1866 | 0.1239 | 1.32E-01 | 0.0939 | -0.0585 | 0.2266 | 7.96E-01 | 0.0939 |
| rs11242899 | 6 | 460302 | A | G | -0.0245 | 0.0024 | 3.20E-24 | 0.2663 | 0.0302 | 0.0823 | 7.13E-01 | 0.2627 | -0.0266 | 0.1496 | 8.59E-01 | 0.2627 |
| rs1126809 | 11 | 89017961 | A | G | 0.0825 | 0.0023 | 1.00E-200 | 0.3028 | 0.2724 | 0.0942 | 3.84E-03 | 0.1768 | 0.4369 | 0.1714 | 1.08E-02 | 0.1768 |
| rs116125333 | 5 | 33930012 | G | T | -0.0397 | 0.0072 | 3.40E-08 | 0.0232 | -0.0948 | 0.1333 | 4.77E-01 | 0.0842 | -0.1747 | 0.2434 | 4.73E-01 | 0.0842 |
| rs11648436 | 16 | 14008674 | T | C | -0.0273 | 0.0022 | 9.90E-35 | 0.3564 | 0.0232 | 0.0784 | 7.68E-01 | 0.3077 | -0.0013 | 0.1425 | 9.93E-01 | 0.3077 |
| rs117132860 | 7 | 17134708 | A | G | 0.0593 | 0.0067 | 1.20E-18 | 0.0256 | 1.0140 | 0.5883 | 8.48E-02 | 0.0039 | 2.5304 | 1.1549 | 2.84E-02 | 0.0039 |
| rs11739906 | 5 | 59019359 | C | A | 0.0130 | 0.0023 | 8.40E-09 | 0.3275 | -0.0393 | 0.0842 | 6.41E-01 | 0.2402 | -0.2024 | 0.1535 | 1.87E-01 | 0.2402 |
| rs117462393 | 16 | 14277804 | T | C | 0.0815 | 0.0096 | 1.50E-17 | 0.0144 | 0.0803 | 0.1489 | 5.89E-01 | 0.0651 | 0.5412 | 0.2689 | 4.42E-02 | 0.0651 |
| rs12203592 | 6 | 396321 | T | C | 0.1531 | 0.0025 | 1.00E-200 | 0.2191 | 0.2520 | 0.2127 | 2.36E-01 | 0.0301 | 0.4557 | 0.3891 | 2.42E-01 | 0.0301 |
| rs1233578 | 6 | 28712247 | G | A | -0.0206 | 0.0028 | 1.50E-13 | 0.1764 | 0.0572 | 0.1486 | 7.00E-01 | 0.0605 | 0.4120 | 0.2738 | 1.32E-01 | 0.0605 |
| rs12350739 | 9 | 16885017 | A | G | 0.0355 | 0.0022 | 5.80E-59 | 0.6068 | 0.0137 | 0.0725 | 8.51E-01 | 0.5456 | 0.0468 | 0.1320 | 7.23E-01 | 0.5456 |
| rs1260326 | 2 | 27730940 | C | T | -0.0125 | 0.0022 | 8.10E-09 | 0.6040 | -0.0900 | 0.0760 | 2.36E-01 | 0.6490 | -0.1655 | 0.1380 | 2.30E-01 | 0.6490 |
| rs1267038 | 2 | 162099153 | C | A | -0.0179 | 0.0030 | 2.20E-09 | 0.8449 | 0.0699 | 0.0946 | 4.60E-01 | 0.8186 | -0.1761 | 0.1712 | 3.04E-01 | 0.8186 |
| rs1278766 | 13 | 113534382 | C | T | 0.0191 | 0.0021 | 3.90E-19 | 0.5457 | 0.0349 | 0.0725 | 6.31E-01 | 0.4872 | 0.0711 | 0.1317 | 5.89E-01 | 0.4872 |
| rs12913832 | 15 | 28365618 | G | A | 0.0605 | 0.0025 | 1.90E-126 | 0.7753 | -0.3093 | 0.1205 | 1.03E-02 | 0.8943 | 0.1083 | 0.2178 | 6.19E-01 | 0.8944 |
| rs1308048 | 1 | 66888542 | C | T | -0.0204 | 0.0022 | 5.30E-21 | 0.4195 | -0.0727 | 0.0731 | 3.20E-01 | 0.4301 | 0.2501 | 0.1326 | 5.93E-02 | 0.4301 |
| rs1326798 | 9 | 12722227 | G | C | 0.0121 | 0.0022 | 3.60E-08 | 0.6206 | 0.0712 | 0.0751 | 3.43E-01 | 0.6350 | 0.0313 | 0.1365 | 8.19E-01 | 0.6350 |
| rs13332673 | 16 | 89940386 | T | G | -0.1021 | 0.0105 | 3.30E-22 | 0.0102 | -0.4909 | 0.4226 | 2.45E-01 | 0.0073 | -1.0702 | 0.7833 | 1.72E-01 | 0.0073 |
| rs139414522 | 16 | 89821155 | C | T | -0.0784 | 0.0091 | 5.40E-18 | 0.0153 | -0.1573 | 0.3829 | 6.81E-01 | 0.0093 | -1.0389 | 0.7142 | 1.46E-01 | 0.0093 |
| rs141817469 | 16 | 89927151 | T | C | -0.0932 | 0.0058 | 1.00E-57 | 0.0351 | -0.0444 | 0.2098 | 8.33E-01 | 0.0318 | -0.1798 | 0.3832 | 6.39E-01 | 0.0318 |
| rs142314514 | 16 | 90130996 | G | A | -0.0816 | 0.0070 | 3.60E-31 | 0.0285 | -0.1115 | 0.2219 | 6.15E-01 | 0.0281 | 0.3344 | 0.4022 | 4.06E-01 | 0.0281 |
| rs1437635 | 11 | 16358722 | A | C | 0.0207 | 0.0029 | 5.20E-13 | 0.1660 | -0.1926 | 0.1140 | 9.14E-02 | 0.1157 | 0.1268 | 0.2072 | 5.41E-01 | 0.1158 |
| rs150527451 | 11 | 68817897 | A | G | 0.0262 | 0.0035 | 4.00E-14 | 0.1064 | -0.1113 | 0.0865 | 1.98E-01 | 0.2343 | -0.2074 | 0.1575 | 1.88E-01 | 0.2343 |
| rs151337382 | 20 | 34204925 | A | T | 0.0575 | 0.0093 | 5.20E-10 | 0.0136 | -0.5831 | 0.5401 | 2.80E-01 | 0.0046 | -0.0514 | 1.0065 | 9.59E-01 | 0.0046 |
| rs1548714 | 17 | 26280204 | C | A | 0.0162 | 0.0027 | 3.00E-09 | 0.8117 | -0.1408 | 0.0927 | 1.29E-01 | 0.8126 | -0.0243 | 0.1687 | 8.85E-01 | 0.8127 |
| rs16891982 | 5 | 33951693 | G | C | 0.2292 | 0.0062 | 1.00E-200 | 0.9709 | 0.2939 | 0.2778 | 2.90E-01 | 0.9821 | 0.3079 | 0.5040 | 5.41E-01 | 0.9821 |
| rs17232484 | 16 | 89861650 | A | G | 0.0610 | 0.0091 | 1.80E-11 | 0.0142 | 0.1548 | 0.2778 | 5.77E-01 | 0.0198 | 0.6582 | 0.5047 | 1.92E-01 | 0.0198 |
| rs1805007 | 16 | 89986117 | T | C | 0.1964 | 0.0035 | 1.00E-200 | 0.1009 | 0.3644 | 0.1475 | 1.35E-02 | 0.0661 | 0.2618 | 0.2689 | 3.30E-01 | 0.0661 |
| rs1805008 | 16 | 89986144 | T | C | 0.1195 | 0.0038 | 1.00E-200 | 0.0866 | 0.3779 | 0.1421 | 7.82E-03 | 0.0675 | 0.7451 | 0.2605 | 4.24E-03 | 0.0675 |
| rs1989483 | 7 | 16942661 | G | A | 0.0125 | 0.0022 | 1.20E-08 | 0.3891 | 0.1046 | 0.0748 | 1.62E-01 | 0.3681 | 0.1060 | 0.1358 | 4.35E-01 | 0.3680 |
| rs2299098 | 7 | 24756377 | C | G | 0.0148 | 0.0027 | 3.60E-08 | 0.1962 | 0.1442 | 0.0995 | 1.47E-01 | 0.1555 | 0.3743 | 0.1796 | 3.71E-02 | 0.1555 |
| rs251468 | 5 | 149194485 | T | C | -0.0282 | 0.0025 | 4.40E-30 | 0.2484 | 0.0138 | 0.0762 | 8.56E-01 | 0.3447 | -0.2152 | 0.1384 | 1.20E-01 | 0.3446 |
| rs2737217 | 8 | 116630311 | G | A | -0.0255 | 0.0022 | 4.10E-32 | 0.5633 | 0.0495 | 0.0769 | 5.20E-01 | 0.6672 | 0.0622 | 0.1394 | 6.56E-01 | 0.6672 |
| rs3213737 | 12 | 96379806 | A | G | -0.0319 | 0.0022 | 2.70E-49 | 0.5760 | 0.0601 | 0.0760 | 4.29E-01 | 0.6553 | 0.0606 | 0.1384 | 6.61E-01 | 0.6553 |
| rs35563099 | 10 | 119572403 | T | C | -0.0310 | 0.0029 | 1.80E-26 | 0.1640 | -0.0580 | 0.0936 | 5.36E-01 | 0.1860 | -0.1415 | 0.1698 | 4.05E-01 | 0.1860 |
| rs3759579 | 14 | 103851272 | G | A | 0.0124 | 0.0022 | 8.10E-09 | 0.5888 | -0.0316 | 0.0747 | 6.73E-01 | 0.6208 | -0.0986 | 0.1355 | 4.67E-01 | 0.6208 |
| rs41563 | 7 | 104852654 | A | G | 0.0135 | 0.0022 | 1.40E-09 | 0.3498 | 0.0335 | 0.0785 | 6.69E-01 | 0.3083 | -0.0833 | 0.1428 | 5.60E-01 | 0.3083 |
| rs4240559 | 6 | 98437775 | C | T | -0.0171 | 0.0021 | 1.40E-15 | 0.5579 | -0.0876 | 0.0757 | 2.47E-01 | 0.6515 | -0.2041 | 0.1379 | 1.39E-01 | 0.6515 |
| rs4272574 | 1 | 73661205 | T | C | 0.0152 | 0.0021 | 8.40E-13 | 0.4783 | -0.0033 | 0.0724 | 9.63E-01 | 0.5101 | 0.2617 | 0.1317 | 4.69E-02 | 0.5101 |
| rs4335021 | 6 | 32386619 | C | T | -0.0118 | 0.0022 | 4.90E-08 | 0.5997 | -0.0824 | 0.0883 | 3.51E-01 | 0.6432 | -0.0072 | 0.1613 | 9.64E-01 | 0.6432 |
| rs4438032 | 11 | 88786692 | G | C | 0.0232 | 0.0036 | 2.00E-10 | 0.9062 | 0.3051 | 0.1413 | 3.08E-02 | 0.9274 | 0.5199 | 0.2567 | 4.28E-02 | 0.9274 |
| rs4578351 | 11 | 16587580 | C | T | -0.0205 | 0.0026 | 1.70E-15 | 0.2220 | -0.0264 | 0.0844 | 7.54E-01 | 0.2502 | -0.0485 | 0.1536 | 7.52E-01 | 0.2502 |
| rs4670813 | 2 | 38317710 | A | G | -0.0127 | 0.0021 | 3.50E-09 | 0.4708 | -0.0527 | 0.0725 | 4.67E-01 | 0.5241 | 0.0802 | 0.1318 | 5.43E-01 | 0.5241 |
| rs4840542 | 8 | 10944809 | T | G | 0.0185 | 0.0021 | 4.00E-18 | 0.5058 | -0.0865 | 0.1407 | 5.39E-01 | 0.4248 | -0.0644 | 0.2553 | 8.01E-01 | 0.4248 |
| rs511515 | 6 | 33541507 | G | A | -0.0204 | 0.0023 | 1.60E-18 | 0.7009 | 0.1981 | 0.0871 | 2.30E-02 | 0.7820 | 0.1114 | 0.1585 | 4.82E-01 | 0.7820 |
| rs537894 | 3 | 138348595 | A | G | 0.0127 | 0.0022 | 5.00E-09 | 0.5490 | 0.0793 | 0.0780 | 3.09E-01 | 0.6725 | 0.3100 | 0.1416 | 2.86E-02 | 0.6725 |
| rs57994353 | 9 | 139356987 | C | T | 0.0127 | 0.0023 | 4.30E-08 | 0.2991 | 0.0484 | 0.0829 | 5.59E-01 | 0.2558 | 0.0768 | 0.1507 | 6.10E-01 | 0.2558 |
| rs6007506 | 22 | 45622014 | T | C | -0.0248 | 0.0023 | 4.10E-28 | 0.3374 | -0.1090 | 0.0753 | 1.48E-01 | 0.3560 | -0.1559 | 0.1367 | 2.54E-01 | 0.3561 |
| rs6059655 | 20 | 32665748 | G | A | -0.1273 | 0.0036 | 1.00E-200 | 0.8980 | -0.4427 | 0.2400 | 6.51E-02 | 0.9778 | -0.9949 | 0.4294 | 2.05E-02 | 0.9778 |
| rs61816766 | 1 | 152319572 | C | T | 0.0366 | 0.0062 | 3.40E-09 | 0.0323 | -0.3262 | 0.3850 | 3.97E-01 | 0.0079 | -0.1013 | 0.6936 | 8.84E-01 | 0.0079 |
| rs61981034 | 14 | 97377089 | A | G | 0.0133 | 0.0024 | 4.60E-08 | 0.2580 | -0.0820 | 0.0766 | 2.84E-01 | 0.3372 | -0.2337 | 0.1386 | 9.18E-02 | 0.3372 |
| rs6689641 | 1 | 110720400 | G | A | 0.0149 | 0.0021 | 2.60E-12 | 0.5426 | 0.0830 | 0.0728 | 2.54E-01 | 0.5585 | 0.0837 | 0.1322 | 5.27E-01 | 0.5585 |
| rs6882046 | 5 | 87968864 | G | A | 0.0144 | 0.0024 | 3.10E-09 | 0.2679 | -0.0858 | 0.0935 | 3.59E-01 | 0.1797 | -0.1850 | 0.1693 | 2.75E-01 | 0.1797 |
| rs699780 | 1 | 120455441 | G | A | -0.0184 | 0.0032 | 7.00E-09 | 0.1278 | 0.0058 | 0.0965 | 9.52E-01 | 0.1680 | 0.0390 | 0.1751 | 8.24E-01 | 0.1680 |
| rs72821630 | 2 | 63696212 | T | C | -0.0150 | 0.0025 | 9.00E-10 | 0.2514 | 0.0445 | 0.0755 | 5.56E-01 | 0.3630 | -0.0172 | 0.1369 | 9.00E-01 | 0.3630 |
| rs75300484 | 16 | 89489061 | T | C | -0.0342 | 0.0058 | 3.70E-09 | 0.0347 | -0.0588 | 0.2303 | 7.98E-01 | 0.0233 | -0.5252 | 0.4202 | 2.11E-01 | 0.0233 |
| rs75908072 | 6 | 290438 | C | T | -0.0318 | 0.0057 | 2.80E-08 | 0.0366 | -0.1844 | 0.2553 | 4.70E-01 | 0.0226 | -0.3135 | 0.4650 | 5.00E-01 | 0.0226 |
| rs7768317 | 6 | 41922220 | T | C | -0.0170 | 0.0025 | 4.90E-12 | 0.2489 | -0.0230 | 0.0824 | 7.80E-01 | 0.2628 | -0.0644 | 0.1495 | 6.67E-01 | 0.2628 |
| rs784235 | 18 | 53423144 | G | A | -0.0164 | 0.0028 | 3.00E-09 | 0.8191 | 0.2163 | 0.1025 | 3.49E-02 | 0.8555 | 0.1848 | 0.1865 | 3.22E-01 | 0.8555 |
| rs78444298 | 1 | 184672098 | A | G | -0.0467 | 0.0077 | 1.40E-09 | 0.0197 | -0.0593 | 0.2768 | 8.31E-01 | 0.0172 | -0.7777 | 0.5119 | 1.29E-01 | 0.0172 |
| rs849138 | 7 | 28177338 | A | G | 0.0126 | 0.0021 | 3.80E-09 | 0.5070 | 0.1585 | 0.0723 | 2.85E-02 | 0.4898 | 0.2635 | 0.1313 | 4.47E-02 | 0.4898 |
| rs9328259 | 6 | 508972 | A | C | 0.0267 | 0.0024 | 1.60E-29 | 0.7182 | -0.0785 | 0.0804 | 3.28E-01 | 0.7206 | 0.0324 | 0.1464 | 8.25E-01 | 0.7207 |
| rs964127 | 12 | 96290733 | T | A | 0.0123 | 0.0023 | 4.90E-08 | 0.3328 | 0.0109 | 0.0726 | 8.80E-01 | 0.4645 | -0.1744 | 0.1316 | 1.85E-01 | 0.4645 |
| rs9821675 | 3 | 49902544 | G | A | -0.0120 | 0.0021 | 1.50E-08 | 0.5053 | -0.0592 | 0.0741 | 4.25E-01 | 0.3955 | -0.2194 | 0.1346 | 1.03E-01 | 0.3955 |
| rs9832130 | 3 | 189194752 | A | G | -0.0128 | 0.0022 | 3.30E-09 | 0.5807 | 0.0518 | 0.0730 | 4.79E-01 | 0.5602 | -0.1483 | 0.1326 | 2.63E-01 | 0.5602 |
| rs9835772 | 3 | 85766025 | T | A | 0.0177 | 0.0025 | 8.60E-13 | 0.2437 | 0.1556 | 0.0859 | 7.01E-02 | 0.2289 | 0.2134 | 0.1558 | 1.71E-01 | 0.2289 |
| rs9858244 | 3 | 85787399 | A | G | 0.0189 | 0.0026 | 3.00E-13 | 0.2142 | 0.1633 | 0.0927 | 7.82E-02 | 0.1846 | 0.0871 | 0.1681 | 6.04E-01 | 0.1845 |
| rs9867857 | 3 | 156491160 | T | C | 0.0136 | 0.0021 | 1.80E-10 | 0.4892 | 0.0896 | 0.0732 | 2.21E-01 | 0.4292 | -0.0014 | 0.1331 | 9.92E-01 | 0.4291 |
| rs9904468 | 17 | 17573187 | G | C | 0.0137 | 0.0021 | 1.10E-10 | 0.5335 | -0.0191 | 0.0724 | 7.92E-01 | 0.5503 | 0.0497 | 0.1316 | 7.06E-01 | 0.5504 |

SNP: Single-nucleotide polymorphisms; Chr: Chromosome; EA: Effect allele; OA: Other allele; EAF: Effect allele frequency.

**Supplementary Table 9.** Information of identified SNPs in exposure (Childhood sunburn) and outcomes (Malignant melanoma of skin and non-melanoma skin cancer, controls excluding all cancers).

| SNP | | | | | Exposure (Childhood sunburn) | | | | Outcome (Melanoma in situ of trunk, controls excluding all cancers) | | | | Outcome (Melanoma in situ of lowerlimb, controls excluding all cancers) | | | | Outcome (Melanoma in situ of upperlimb, controls excluding all cancers) | | | |
| --- | --- | --- | --- | --- | --- | --- | --- | --- | --- | --- | --- | --- | --- | --- | --- | --- | --- | --- | --- | --- |
| RS ID | Chr | Position | EA | OA | Beta | Se | P-  value | EAF | Beta | Se | P-  value | EAF | Beta | Se | P-  value | EAF | Beta | Se | P-  value | EAF |
| rs10168349 | 2 | 46360907 | C | G | 0.0173 | 0.0023 | 1.40E-14 | 0.3357 | -0.1341 | 0.1545 | 3.85E-01 | 0.2823 | 0.1200 | 0.1582 | 4.48E-01 | 0.2821 | 0.0205 | 0.1678 | 9.03E-01 | 0.2821 |
| rs10202908 | 2 | 169378292 | T | C | -0.0126 | 0.0023 | 2.90E-08 | 0.6749 | -0.0265 | 0.1508 | 8.60E-01 | 0.6988 | -0.1035 | 0.1560 | 5.07E-01 | 0.6991 | -0.3794 | 0.1642 | 2.08E-02 | 0.6991 |
| rs10220751 | 15 | 47923520 | G | T | -0.0137 | 0.0022 | 2.50E-10 | 0.4048 | -0.2021 | 0.1440 | 1.60E-01 | 0.3601 | 0.0307 | 0.1473 | 8.35E-01 | 0.3601 | 0.1050 | 0.1564 | 5.02E-01 | 0.3601 |
| rs10788627 | 10 | 82203069 | C | T | 0.0120 | 0.0021 | 1.60E-08 | 0.4745 | -0.1191 | 0.1393 | 3.93E-01 | 0.5466 | 0.0015 | 0.1435 | 9.92E-01 | 0.5469 | 0.0261 | 0.1503 | 8.62E-01 | 0.5469 |
| rs10810636 | 9 | 16799109 | G | A | 0.0275 | 0.0025 | 2.80E-28 | 0.7603 | -0.0157 | 0.1575 | 9.21E-01 | 0.7366 | 0.2526 | 0.1629 | 1.21E-01 | 0.7362 | 0.0021 | 0.1710 | 9.90E-01 | 0.7362 |
| rs10873552 | 14 | 105433129 | G | A | 0.0124 | 0.0022 | 3.70E-08 | 0.6577 | 0.0406 | 0.1404 | 7.72E-01 | 0.5738 | 0.2272 | 0.1447 | 1.16E-01 | 0.5730 | -0.0389 | 0.1526 | 7.99E-01 | 0.5730 |
| rs10896139 | 11 | 66650060 | T | C | -0.0136 | 0.0024 | 1.40E-08 | 0.2709 | 0.0289 | 0.1546 | 8.52E-01 | 0.2783 | -0.0120 | 0.1598 | 9.40E-01 | 0.2785 | -0.0927 | 0.1685 | 5.82E-01 | 0.2784 |
| rs11070811 | 15 | 31394082 | T | C | -0.0176 | 0.0027 | 1.50E-10 | 0.1832 | -0.3794 | 0.2011 | 5.92E-02 | 0.1332 | -0.1989 | 0.2055 | 3.33E-01 | 0.1339 | -0.0608 | 0.2200 | 7.82E-01 | 0.1339 |
| rs11104733 | 12 | 88490119 | T | C | 0.0492 | 0.0089 | 2.80E-08 | 0.0147 | 0.5245 | 0.4690 | 2.64E-01 | 0.0236 | 0.0710 | 0.4700 | 8.80E-01 | 0.0236 | -0.7619 | 0.5086 | 1.34E-01 | 0.0236 |
| rs111391498 | 4 | 1341553 | G | A | -0.0474 | 0.0050 | 1.70E-21 | 0.0479 | -0.3297 | 0.4179 | 4.30E-01 | 0.0285 | -0.1061 | 0.4282 | 8.04E-01 | 0.0286 | -0.0009 | 0.4574 | 9.99E-01 | 0.0286 |
| rs111650620 | 20 | 34591725 | A | G | 0.6618 | 0.0601 | 3.20E-28 | 0.0022 | 0.1023 | 0.4323 | 8.13E-01 | 0.0272 | -0.4534 | 0.4452 | 3.08E-01 | 0.0272 | 0.0052 | 0.4581 | 9.91E-01 | 0.0273 |
| rs112089506 | 16 | 90149171 | T | C | -0.0581 | 0.0040 | 1.40E-47 | 0.0812 | -0.4082 | 0.2390 | 8.76E-02 | 0.0939 | -0.1933 | 0.2435 | 4.27E-01 | 0.0943 | -0.1280 | 0.2581 | 6.20E-01 | 0.0943 |
| rs11242899 | 6 | 460302 | A | G | -0.0245 | 0.0024 | 3.20E-24 | 0.2663 | -0.1589 | 0.1575 | 3.13E-01 | 0.2627 | 0.1472 | 0.1627 | 3.65E-01 | 0.2612 | -0.0652 | 0.1723 | 7.05E-01 | 0.2612 |
| rs1126809 | 11 | 89017961 | A | G | 0.0825 | 0.0023 | 1.00E-200 | 0.3028 | 0.1683 | 0.1800 | 3.50E-01 | 0.1768 | 0.1647 | 0.1860 | 3.76E-01 | 0.1771 | 0.2197 | 0.1962 | 2.63E-01 | 0.1771 |
| rs116125333 | 5 | 33930012 | G | T | -0.0397 | 0.0072 | 3.40E-08 | 0.0232 | -0.2263 | 0.2589 | 3.82E-01 | 0.0842 | -0.3190 | 0.2663 | 2.31E-01 | 0.0848 | 0.1881 | 0.2786 | 5.00E-01 | 0.0848 |
| rs11648436 | 16 | 14008674 | T | C | -0.0273 | 0.0022 | 9.90E-35 | 0.3564 | 0.0417 | 0.1508 | 7.82E-01 | 0.3077 | -0.1300 | 0.1549 | 4.01E-01 | 0.3068 | 0.1425 | 0.1635 | 3.83E-01 | 0.3068 |
| rs117132860 | 7 | 17134708 | A | G | 0.0593 | 0.0067 | 1.20E-18 | 0.0256 | 0.2244 | 1.1238 | 8.42E-01 | 0.0039 | -1.1080 | 1.2273 | 3.67E-01 | 0.0039 | 3.1096 | 1.2599 | 1.36E-02 | 0.0039 |
| rs11739906 | 5 | 59019359 | C | A | 0.0130 | 0.0023 | 8.40E-09 | 0.3275 | 0.0489 | 0.1614 | 7.62E-01 | 0.2403 | 0.0805 | 0.1650 | 6.26E-01 | 0.2403 | 0.0567 | 0.1750 | 7.46E-01 | 0.2403 |
| rs117462393 | 16 | 14277804 | T | C | 0.0815 | 0.0096 | 1.50E-17 | 0.0144 | 0.0473 | 0.2889 | 8.70E-01 | 0.0651 | -0.2305 | 0.2937 | 4.33E-01 | 0.0654 | -0.1662 | 0.3089 | 5.91E-01 | 0.0654 |
| rs12203592 | 6 | 396321 | T | C | 0.1531 | 0.0025 | 1.00E-200 | 0.2191 | 0.4148 | 0.4037 | 3.04E-01 | 0.0301 | 0.2522 | 0.4149 | 5.43E-01 | 0.0304 | -0.3460 | 0.4454 | 4.37E-01 | 0.0304 |
| rs1233578 | 6 | 28712247 | G | A | -0.0206 | 0.0028 | 1.50E-13 | 0.1764 | -0.4337 | 0.2691 | 1.07E-01 | 0.0604 | -0.8331 | 0.3285 | 1.12E-02 | 0.0609 | 0.4543 | 0.3014 | 1.32E-01 | 0.0609 |
| rs12350739 | 9 | 16885017 | A | G | 0.0355 | 0.0022 | 5.80E-59 | 0.6068 | -0.1953 | 0.1390 | 1.60E-01 | 0.5456 | 0.0068 | 0.1427 | 9.62E-01 | 0.5448 | 0.2948 | 0.1507 | 5.04E-02 | 0.5449 |
| rs1260326 | 2 | 27730940 | C | T | -0.0125 | 0.0022 | 8.10E-09 | 0.6040 | 0.0204 | 0.1456 | 8.88E-01 | 0.6490 | -0.2704 | 0.1498 | 7.11E-02 | 0.6498 | -0.1361 | 0.1579 | 3.89E-01 | 0.6498 |
| rs1267038 | 2 | 162099153 | C | A | -0.0179 | 0.0030 | 2.20E-09 | 0.8449 | 0.1434 | 0.1811 | 4.29E-01 | 0.8186 | 0.3558 | 0.1877 | 5.80E-02 | 0.8187 | -0.0824 | 0.1965 | 6.75E-01 | 0.8187 |
| rs1278766 | 13 | 113534382 | C | T | 0.0191 | 0.0021 | 3.90E-19 | 0.5457 | 0.1672 | 0.1390 | 2.29E-01 | 0.4872 | 0.0002 | 0.1425 | 9.99E-01 | 0.4861 | -0.1903 | 0.1515 | 2.09E-01 | 0.4861 |
| rs12913832 | 15 | 28365618 | G | A | 0.0605 | 0.0025 | 1.90E-126 | 0.7753 | -0.4245 | 0.2305 | 6.55E-02 | 0.8944 | -0.1949 | 0.2421 | 4.21E-01 | 0.8959 | -0.6406 | 0.2537 | 1.16E-02 | 0.8958 |
| rs1308048 | 1 | 66888542 | C | T | -0.0204 | 0.0022 | 5.30E-21 | 0.4195 | -0.3227 | 0.1405 | 2.17E-02 | 0.4301 | -0.1329 | 0.1439 | 3.55E-01 | 0.4303 | 0.0743 | 0.1523 | 6.26E-01 | 0.4304 |
| rs1326798 | 9 | 12722227 | G | C | 0.0121 | 0.0022 | 3.60E-08 | 0.6206 | -0.1688 | 0.1443 | 2.42E-01 | 0.6350 | 0.3480 | 0.1478 | 1.86E-02 | 0.6354 | -0.0164 | 0.1562 | 9.16E-01 | 0.6354 |
| rs13332673 | 16 | 89940386 | T | G | -0.1021 | 0.0105 | 3.30E-22 | 0.0102 | 0.3157 | 0.8256 | 7.02E-01 | 0.0073 | -0.1978 | 0.9242 | 8.31E-01 | 0.0066 | -0.0869 | 0.9429 | 9.27E-01 | 0.0066 |
| rs139414522 | 16 | 89821155 | C | T | -0.0784 | 0.0091 | 5.40E-18 | 0.0153 | 0.0373 | 0.7265 | 9.59E-01 | 0.0093 | 0.7191 | 0.7636 | 3.46E-01 | 0.0093 | -0.4223 | 0.7624 | 5.80E-01 | 0.0093 |
| rs141817469 | 16 | 89927151 | T | C | -0.0932 | 0.0058 | 1.00E-57 | 0.0351 | -0.5321 | 0.4056 | 1.90E-01 | 0.0318 | -0.2533 | 0.4142 | 5.41E-01 | 0.0321 | 0.2878 | 0.4368 | 5.10E-01 | 0.0321 |
| rs142314514 | 16 | 90130996 | G | A | -0.0816 | 0.0070 | 3.60E-31 | 0.0285 | -0.0294 | 0.4313 | 9.46E-01 | 0.0281 | 0.0105 | 0.4446 | 9.81E-01 | 0.0279 | -0.0157 | 0.4607 | 9.73E-01 | 0.0279 |
| rs1437635 | 11 | 16358722 | A | C | 0.0207 | 0.0029 | 5.20E-13 | 0.1660 | -0.2120 | 0.2187 | 3.32E-01 | 0.1158 | -0.1374 | 0.2223 | 5.37E-01 | 0.1157 | -0.2695 | 0.2367 | 2.55E-01 | 0.1157 |
| rs150527451 | 11 | 68817897 | A | G | 0.0262 | 0.0035 | 4.00E-14 | 0.1064 | 0.2416 | 0.1668 | 1.47E-01 | 0.2343 | -0.1193 | 0.1713 | 4.86E-01 | 0.2339 | -0.0440 | 0.1787 | 8.05E-01 | 0.2339 |
| rs151337382 | 20 | 34204925 | A | T | 0.0575 | 0.0093 | 5.20E-10 | 0.0136 | -0.9554 | 0.9986 | 3.39E-01 | 0.0046 | -1.0467 | 1.1215 | 3.51E-01 | 0.0045 | -0.1435 | 1.2066 | 9.05E-01 | 0.0045 |
| rs1548714 | 17 | 26280204 | C | A | 0.0162 | 0.0027 | 3.00E-09 | 0.8117 | -0.1033 | 0.1772 | 5.60E-01 | 0.8127 | -0.3086 | 0.1831 | 9.20E-02 | 0.8127 | -0.2525 | 0.1935 | 1.92E-01 | 0.8127 |
| rs16891982 | 5 | 33951693 | G | C | 0.2292 | 0.0062 | 1.00E-200 | 0.9709 | 0.1380 | 0.5331 | 7.96E-01 | 0.9821 | 0.3966 | 0.6024 | 5.10E-01 | 0.9833 | 0.5479 | 0.6382 | 3.91E-01 | 0.9833 |
| rs17232484 | 16 | 89861650 | A | G | 0.0610 | 0.0091 | 1.80E-11 | 0.0142 | -0.2571 | 0.5364 | 6.32E-01 | 0.0198 | 0.2439 | 0.5569 | 6.61E-01 | 0.0200 | 0.0883 | 0.5924 | 8.82E-01 | 0.0200 |
| rs1805007 | 16 | 89986117 | T | C | 0.1964 | 0.0035 | 1.00E-200 | 0.1009 | 0.4783 | 0.2837 | 9.18E-02 | 0.0661 | 0.3831 | 0.2967 | 1.97E-01 | 0.0654 | 0.5796 | 0.3141 | 6.50E-02 | 0.0654 |
| rs1805008 | 16 | 89986144 | T | C | 0.1195 | 0.0038 | 1.00E-200 | 0.0866 | 0.3974 | 0.2706 | 1.42E-01 | 0.0675 | 0.0122 | 0.2790 | 9.65E-01 | 0.0677 | -0.1134 | 0.2973 | 7.03E-01 | 0.0677 |
| rs1989483 | 7 | 16942661 | G | A | 0.0125 | 0.0022 | 1.20E-08 | 0.3891 | -0.0456 | 0.1437 | 7.51E-01 | 0.3680 | 0.0896 | 0.1473 | 5.43E-01 | 0.3687 | 0.1816 | 0.1560 | 2.44E-01 | 0.3687 |
| rs2299098 | 7 | 24756377 | C | G | 0.0148 | 0.0027 | 3.60E-08 | 0.1962 | 0.2787 | 0.1906 | 1.44E-01 | 0.1554 | -0.2787 | 0.1954 | 1.54E-01 | 0.1552 | -0.4531 | 0.2070 | 2.86E-02 | 0.1552 |
| rs251468 | 5 | 149194485 | T | C | -0.0282 | 0.0025 | 4.40E-30 | 0.2484 | 0.0456 | 0.1468 | 7.56E-01 | 0.3446 | -0.2224 | 0.1506 | 1.40E-01 | 0.3443 | -0.0304 | 0.1589 | 8.49E-01 | 0.3443 |
| rs2737217 | 8 | 116630311 | G | A | -0.0255 | 0.0022 | 4.10E-32 | 0.5633 | -0.0807 | 0.1471 | 5.83E-01 | 0.6671 | 0.2964 | 0.1518 | 5.08E-02 | 0.6662 | 0.0817 | 0.1595 | 6.09E-01 | 0.6662 |
| rs3213737 | 12 | 96379806 | A | G | -0.0319 | 0.0022 | 2.70E-49 | 0.5760 | 0.0042 | 0.1458 | 9.77E-01 | 0.6553 | 0.0654 | 0.1500 | 6.63E-01 | 0.6556 | -0.0680 | 0.1583 | 6.68E-01 | 0.6556 |
| rs35563099 | 10 | 119572403 | T | C | -0.0310 | 0.0029 | 1.80E-26 | 0.1640 | 0.0203 | 0.1799 | 9.10E-01 | 0.1860 | -0.3550 | 0.1861 | 5.64E-02 | 0.1845 | -0.4298 | 0.1943 | 2.70E-02 | 0.1845 |
| rs3759579 | 14 | 103851272 | G | A | 0.0124 | 0.0022 | 8.10E-09 | 0.5888 | -0.0824 | 0.1428 | 5.64E-01 | 0.6208 | 0.0043 | 0.1473 | 9.77E-01 | 0.6196 | -0.1306 | 0.1547 | 3.99E-01 | 0.6196 |
| rs41563 | 7 | 104852654 | A | G | 0.0135 | 0.0022 | 1.40E-09 | 0.3498 | 0.0738 | 0.1510 | 6.25E-01 | 0.3083 | -0.0482 | 0.1546 | 7.55E-01 | 0.3076 | -0.1911 | 0.1628 | 2.40E-01 | 0.3076 |
| rs4240559 | 6 | 98437775 | C | T | -0.0171 | 0.0021 | 1.40E-15 | 0.5579 | -0.1612 | 0.1452 | 2.67E-01 | 0.6515 | 0.1071 | 0.1496 | 4.74E-01 | 0.6522 | 0.0745 | 0.1578 | 6.37E-01 | 0.6522 |
| rs4272574 | 1 | 73661205 | T | C | 0.0152 | 0.0021 | 8.40E-13 | 0.4783 | -0.1355 | 0.1389 | 3.29E-01 | 0.5101 | -0.1237 | 0.1427 | 3.86E-01 | 0.5097 | 0.0486 | 0.1510 | 7.48E-01 | 0.5097 |
| rs4335021 | 6 | 32386619 | C | T | -0.0118 | 0.0022 | 4.90E-08 | 0.5997 | -0.3914 | 0.1691 | 2.06E-02 | 0.6432 | 0.0230 | 0.1476 | 8.76E-01 | 0.6427 | -0.2454 | 0.1560 | 1.16E-01 | 0.6426 |
| rs4438032 | 11 | 88786692 | G | C | 0.0232 | 0.0036 | 2.00E-10 | 0.9062 | 0.3424 | 0.2692 | 2.04E-01 | 0.9274 | 0.1929 | 0.2770 | 4.86E-01 | 0.9273 | -0.6706 | 0.2922 | 2.17E-02 | 0.9273 |
| rs4578351 | 11 | 16587580 | C | T | -0.0205 | 0.0026 | 1.70E-15 | 0.2220 | -0.1164 | 0.1621 | 4.73E-01 | 0.2502 | 0.0263 | 0.1688 | 8.76E-01 | 0.2482 | -0.1337 | 0.1764 | 4.48E-01 | 0.2482 |
| rs4670813 | 2 | 38317710 | A | G | -0.0127 | 0.0021 | 3.50E-09 | 0.4708 | -0.1173 | 0.1386 | 3.97E-01 | 0.5241 | -0.2123 | 0.1436 | 1.39E-01 | 0.5247 | 0.0884 | 0.1504 | 5.57E-01 | 0.5247 |
| rs4840542 | 8 | 10944809 | T | G | 0.0185 | 0.0021 | 4.00E-18 | 0.5058 | 0.0866 | 0.2649 | 7.44E-01 | 0.4248 | -0.3165 | 0.1440 | 2.80E-02 | 0.4256 | -0.0004 | 0.1520 | 9.98E-01 | 0.4256 |
| rs511515 | 6 | 33541507 | G | A | -0.0204 | 0.0023 | 1.60E-18 | 0.7009 | 0.2475 | 0.1652 | 1.34E-01 | 0.7820 | 0.3095 | 0.1713 | 7.07E-02 | 0.7814 | 0.0375 | 0.1818 | 8.36E-01 | 0.7814 |
| rs537894 | 3 | 138348595 | A | G | 0.0127 | 0.0022 | 5.00E-09 | 0.5490 | -0.1433 | 0.1498 | 3.39E-01 | 0.6725 | 0.1024 | 0.1537 | 5.05E-01 | 0.6733 | 0.3790 | 0.1625 | 1.97E-02 | 0.6733 |
| rs57994353 | 9 | 139356987 | C | T | 0.0127 | 0.0023 | 4.30E-08 | 0.2991 | -0.0743 | 0.1586 | 6.40E-01 | 0.2558 | -0.0898 | 0.1635 | 5.83E-01 | 0.2548 | -0.0154 | 0.1720 | 9.29E-01 | 0.2548 |
| rs6007506 | 22 | 45622014 | T | C | -0.0248 | 0.0023 | 4.10E-28 | 0.3374 | -0.2801 | 0.1448 | 5.31E-02 | 0.3560 | 0.0181 | 0.1482 | 9.03E-01 | 0.3557 | -0.1755 | 0.1571 | 2.64E-01 | 0.3557 |
| rs6059655 | 20 | 32665748 | G | A | -0.1273 | 0.0036 | 1.00E-200 | 0.8980 | -0.6386 | 0.4553 | 1.61E-01 | 0.9778 | 0.8065 | 0.4739 | 8.88E-02 | 0.9779 | -1.0700 | 0.5132 | 3.71E-02 | 0.9779 |
| rs61816766 | 1 | 152319572 | C | T | 0.0366 | 0.0062 | 3.40E-09 | 0.0323 | -1.0171 | 0.7295 | 1.63E-01 | 0.0079 | 0.1627 | 0.7779 | 8.34E-01 | 0.0079 | -1.0341 | 0.8263 | 2.11E-01 | 0.0079 |
| rs61981034 | 14 | 97377089 | A | G | 0.0133 | 0.0024 | 4.60E-08 | 0.2580 | -0.0743 | 0.1466 | 6.12E-01 | 0.3372 | 0.3453 | 0.1510 | 2.22E-02 | 0.3381 | -0.0042 | 0.1590 | 9.79E-01 | 0.3381 |
| rs6689641 | 1 | 110720400 | G | A | 0.0149 | 0.0021 | 2.60E-12 | 0.5426 | 0.2160 | 0.1396 | 1.22E-01 | 0.5585 | 0.0602 | 0.1432 | 6.74E-01 | 0.5583 | 0.1432 | 0.1515 | 3.45E-01 | 0.5583 |
| rs6882046 | 5 | 87968864 | G | A | 0.0144 | 0.0024 | 3.10E-09 | 0.2679 | -0.0903 | 0.1780 | 6.12E-01 | 0.1797 | 0.1261 | 0.1851 | 4.96E-01 | 0.1799 | 0.0460 | 0.1956 | 8.14E-01 | 0.1799 |
| rs699780 | 1 | 120455441 | G | A | -0.0184 | 0.0032 | 7.00E-09 | 0.1278 | -0.1617 | 0.1854 | 3.83E-01 | 0.1680 | -0.2524 | 0.1904 | 1.85E-01 | 0.1683 | -0.2211 | 0.2005 | 2.70E-01 | 0.1683 |
| rs72821630 | 2 | 63696212 | T | C | -0.0150 | 0.0025 | 9.00E-10 | 0.2514 | 0.1037 | 0.1452 | 4.75E-01 | 0.3630 | 0.0511 | 0.1498 | 7.33E-01 | 0.3630 | -0.1020 | 0.1569 | 5.16E-01 | 0.3630 |
| rs75300484 | 16 | 89489061 | T | C | -0.0342 | 0.0058 | 3.70E-09 | 0.0347 | -0.6402 | 0.4391 | 1.45E-01 | 0.0233 | -0.0699 | 0.4405 | 8.74E-01 | 0.0234 | 0.6695 | 0.4801 | 1.63E-01 | 0.0234 |
| rs75908072 | 6 | 290438 | C | T | -0.0318 | 0.0057 | 2.80E-08 | 0.0366 | 0.1518 | 0.4896 | 7.57E-01 | 0.0226 | 0.0155 | 0.4968 | 9.75E-01 | 0.0231 | -0.5776 | 0.5522 | 2.96E-01 | 0.0231 |
| rs7768317 | 6 | 41922220 | T | C | -0.0170 | 0.0025 | 4.90E-12 | 0.2489 | -0.0763 | 0.1579 | 6.29E-01 | 0.2629 | 0.1983 | 0.1624 | 2.22E-01 | 0.2627 | 0.0510 | 0.1723 | 7.67E-01 | 0.2627 |
| rs784235 | 18 | 53423144 | G | A | -0.0164 | 0.0028 | 3.00E-09 | 0.8191 | 0.1630 | 0.1962 | 4.06E-01 | 0.8555 | 0.0489 | 0.2018 | 8.09E-01 | 0.8560 | 0.4872 | 0.2144 | 2.30E-02 | 0.8561 |
| rs78444298 | 1 | 184672098 | A | G | -0.0467 | 0.0077 | 1.40E-09 | 0.0197 | -0.7266 | 0.5370 | 1.76E-01 | 0.0172 | 0.4162 | 0.5354 | 4.37E-01 | 0.0173 | 0.6794 | 0.5868 | 2.47E-01 | 0.0173 |
| rs849138 | 7 | 28177338 | A | G | 0.0126 | 0.0021 | 3.80E-09 | 0.5070 | 0.1451 | 0.1390 | 2.97E-01 | 0.4898 | 0.0830 | 0.1425 | 5.60E-01 | 0.4898 | 0.1256 | 0.1506 | 4.04E-01 | 0.4898 |
| rs9328259 | 6 | 508972 | A | C | 0.0267 | 0.0024 | 1.60E-29 | 0.7182 | -0.1846 | 0.1537 | 2.30E-01 | 0.7206 | 0.2801 | 0.1585 | 7.72E-02 | 0.7209 | 0.0888 | 0.1671 | 5.95E-01 | 0.7209 |
| rs964127 | 12 | 96290733 | T | A | 0.0123 | 0.0023 | 4.90E-08 | 0.3328 | -0.0155 | 0.1392 | 9.11E-01 | 0.4645 | 0.0255 | 0.1434 | 8.59E-01 | 0.4645 | 0.1545 | 0.1514 | 3.08E-01 | 0.4645 |
| rs9821675 | 3 | 49902544 | G | A | -0.0120 | 0.0021 | 1.50E-08 | 0.5053 | -0.0326 | 0.1416 | 8.18E-01 | 0.3955 | -0.1069 | 0.1458 | 4.64E-01 | 0.3957 | 0.0195 | 0.1537 | 8.99E-01 | 0.3957 |
| rs9832130 | 3 | 189194752 | A | G | -0.0128 | 0.0022 | 3.30E-09 | 0.5807 | -0.0541 | 0.1397 | 6.99E-01 | 0.5602 | 0.0953 | 0.1450 | 5.11E-01 | 0.5628 | 0.1786 | 0.1531 | 2.43E-01 | 0.5628 |
| rs9835772 | 3 | 85766025 | T | A | 0.0177 | 0.0025 | 8.60E-13 | 0.2437 | 0.1948 | 0.1649 | 2.37E-01 | 0.2289 | 0.2395 | 0.1692 | 1.57E-01 | 0.2292 | 0.3115 | 0.1792 | 8.22E-02 | 0.2292 |
| rs9858244 | 3 | 85787399 | A | G | 0.0189 | 0.0026 | 3.00E-13 | 0.2142 | 0.1904 | 0.1781 | 2.85E-01 | 0.1846 | 0.2236 | 0.1820 | 2.19E-01 | 0.1848 | 0.2834 | 0.1929 | 1.42E-01 | 0.1848 |
| rs9867857 | 3 | 156491160 | T | C | 0.0136 | 0.0021 | 1.80E-10 | 0.4892 | 0.2448 | 0.1403 | 8.10E-02 | 0.4292 | -0.0892 | 0.1441 | 5.36E-01 | 0.4282 | -0.0987 | 0.1518 | 5.15E-01 | 0.4282 |
| rs9904468 | 17 | 17573187 | G | C | 0.0137 | 0.0021 | 1.10E-10 | 0.5335 | -0.0098 | 0.1390 | 9.44E-01 | 0.5504 | 0.0493 | 0.1431 | 7.31E-01 | 0.5498 | 0.0836 | 0.1505 | 5.79E-01 | 0.5498 |

SNP: Single-nucleotide polymorphisms; Chr: Chromosome; EA: Effect allele; OA: Other allele; EAF: Effect allele frequency.
